# Ring Finger Protein 1, a Novel Ubiquitin E3 Ligase Targeting Cancerous Inhibitor of Protein Phosphatase 2A to Suppress Smoking‐Induced Lung Tumorigenesis

**DOI:** 10.1002/mco2.70522

**Published:** 2025-12-07

**Authors:** In‐ho Jeong, Jae Kwang Yun, Jun‐O. Jin, Geun Dong Lee, Peter Chang‐Whan Lee

**Affiliations:** ^1^ Department of Biochemistry and Molecular Biology Asan Medical Center University of Ulsan College of Medicine Seoul South Korea; ^2^ Lung Cancer Research Center Asan Medical Center University of Ulsan College of Medicine Seoul South Korea; ^3^ Department of Thoracic and Cardiovascular Surgery Asan Medical Center University of Ulsan College of Medicine Seoul South Korea; ^4^ Department of Microbiology Asan Medical Center University of Ulsan College of Medicine Seoul South Korea

**Keywords:** cancerous inhibitor of protein phosphatase 2A, DNA methyltransferase 1, lung cancer, ring finger protein 1, ubiquitin–proteasome system

## Abstract

Cancerous inhibitor of protein phosphatase 2A (CIP2A) is an oncoprotein that promotes cancer cell proliferation, invasion, and drug resistance. In this study, CIP2A expression was found to be higher in lung cancer tissues compared to adjacent normal tissues. Knocking down CIP2A in lung cancer cells reduced cell proliferation and migration. Ring finger protein 1 (RING1), a member of the RING family in the ubiquitin–proteasome system, was identified as a potential E3 ligase for CIP2A. The interaction between RING1 and CIP2A was confirmed, with RING1 regulating CIP2A ubiquitination. Knockdown of RING1 increased lung cancer cell proliferation and migration in vitro and in vivo, linked to the upregulation of CIP2A and its downstream molecules, c‐MYC and Cyclin B1. Smoking's impact on RING1 expression was examined using cigarette smoke extract (CSE), which decreased RING1 mRNA and protein levels. This led to CIP2A and c‐MYC upregulation. The carcinogen 4‐(methylnitrosamino)‐1‐(3‐pyridyl)‐1‐butanone (NNK), a constituent of CSE, downregulated RING1 expression through DNA methyltransferase 1 (DNMT1) activation, whereas inhibition of DNMT1 restored RING1 levels. These findings highlight the DNMT1–RING1–CIP2A axis in lung cancer progression due to smoking and suggest potential therapeutic and diagnostic targets.

## Graphical abstract

Schematic illustration of the roles of CIP2A and RING1 in lung tumorigenesis

Accumulation of DNMT1 due to NNK exposure leads to a reduction in RING1 mRNA levels. Decreased RING1 results in an accumulation of CIP2A protein levels and upregulated CIP2A suppresses PP2A activity, consequently increasing the stability of c‐MYC. Image was created with BioRender

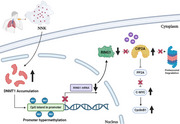

## 1 Introduction

Lung cancer is a leading cause of cancer‐related deaths globally, with an estimated 1.79 million deaths in 2022 [1]. Lung cancer is a heterogeneous disease with two major subtypes: non‐small cell (NSCLC) and small cell lung cancer (SCLC). NSCLC is the most common subtype, comprising approximately 83% of all lung cancer cases, while SCLC accounts for approximately 13% [2]. Tobacco smoking is the primary risk factor for lung cancer, causing approximately 80% of all cases [3]. Other risk factors include exposure to environmental pollutants, including radon, asbestos, and air pollution, as well as genetic predisposition and family health history [4, 5, 6, 7, 8].

Cancerous inhibitor of protein phosphatase 2A (CIP2A) is a newly identified oncoprotein and a promising therapeutic target for cancer [9]. CIP2A was first identified as a protein that inhibits the tumor suppressor protein phosphatase 2A (PP2A), promoting cell proliferation and survival [10]. Since then, studies have shown that CIP2A plays a role in the development and progression of various cancer types, including breast, lung, gastric, colorectal, pancreatic, and hepatocellular carcinomas [11, 12, 13, 14, 15, 16, 17]. CIP2A is a protein predominantly located in the cytoplasm. It interacts with PP2A [18], inhibiting its ability to dephosphorylate various targets involved in cell proliferation and survival, including c‐MYC [19], AKT [20, 21, 22], and E2f1 [23, 24, 25]. By inhibiting PP2A, CIP2A activates oncogenic signaling pathways, thereby enhancing tumor growth and progression. Therefore, CIP2A is emerging as a key regulator of cell survival and death, with its dysregulation contributing to cancer development and progression.

The ubiquitin–proteasome system (UPS) is a highly conserved and tightly regulated pathway essential for degrading intracellular proteins [26]. It selectively degrades proteins that are no longer required, damaged, or misfolded and regulates protein turnover in diverse cellular processes [27]. One of the E3 ligases, Ring finger protein 1 (RING1), is a member of the polycomb group (PcG) of transcriptional regulators that is crucial in regulating gene expression during development and differentiation [28]. Recent studies identify RING1 as a crucial regulator of cellular processes, including cell proliferation [29], differentiation [30, 31], and apoptosis [32]. Dysregulation of RING1 expression or activity is involved in the development and progression of various diseases, including cancer [33], neurological disorders [31], and immune disorders [30]. In cancer, RING1 plays a critical role in regulating oncogenic signaling pathways, such as the Wnt/β‐catenin [34] and Notch [35], and promotes cancer cell survival and growth [36]. For instance, in hepatocellular carcinoma, RING1 promotes cancer stem cell self‐renewal and metastasis through the Wnt/β‐catenin pathway activation. Similarly, in breast cancer, RING1 expression correlates with poor prognosis and chemotherapy resistance [37].

Cigarette smoking is the major risk factor accounting for 80%–90% of all lung cancer diagnoses. 4‐(Methylnitrosamino)‐1‐(3‐pyridyl)‐1‐butanone (NNK) is a potent carcinogen present in tobacco smoke and smokeless tobacco products [38, 39]. NNK causes lung [39, 40, 41, 42, 43, 44], pancreatic [45, 46, 47], bladder [48, 49, 50, 51, 52], and other types of cancer in humans and animals [53, 54]. NNK exerts its carcinogenic effects by inducing DNA damage [55], oxidative stress [56, 57], inflammation [58, 59, 60], and cellular signaling alterations, which activate oncogenic pathways and suppress tumor suppressor pathways [61, 62]. NNK is metabolized in the body into reactive intermediates that bind to DNA, creating DNA adducts and leading to mutations and chromosomal aberrations [63, 64]. NNK also accumulates DNA methyltransferase 1 (DNMT1) in the nucleus through the AKT‐GSK3β‐βTrCP pathway. Accumulated DNMT1 methylates CpG islands in the promoters of numerous tumor suppressor genes (TSGs), thereby promoting tumorigenesis [65, 66].

In this study, we showed that CIP2A functions as an oncogene in lung cancer that upregulated the c‐MYC and Cyclin B1. Also, it is discovered that RING1 interacts with and regulate CIP2A expression by UPS. Moreover, cigarette smoke extract (CSE) treatment caused reduction of RING1 gene expression. These effects came from NNK, a carcinogen in CSE, and its target protein DNMT1. By summarizing the current understanding of the molecular mechanisms of lung cancer development and progression, we aim to propose CIP2A and RING1 as novel therapeutic targets for treating lung cancer.

## 2 Results

### 2.1 CIP2A is Highly Expressed in Lung Cancer Tissues

**FIGURE 1:**
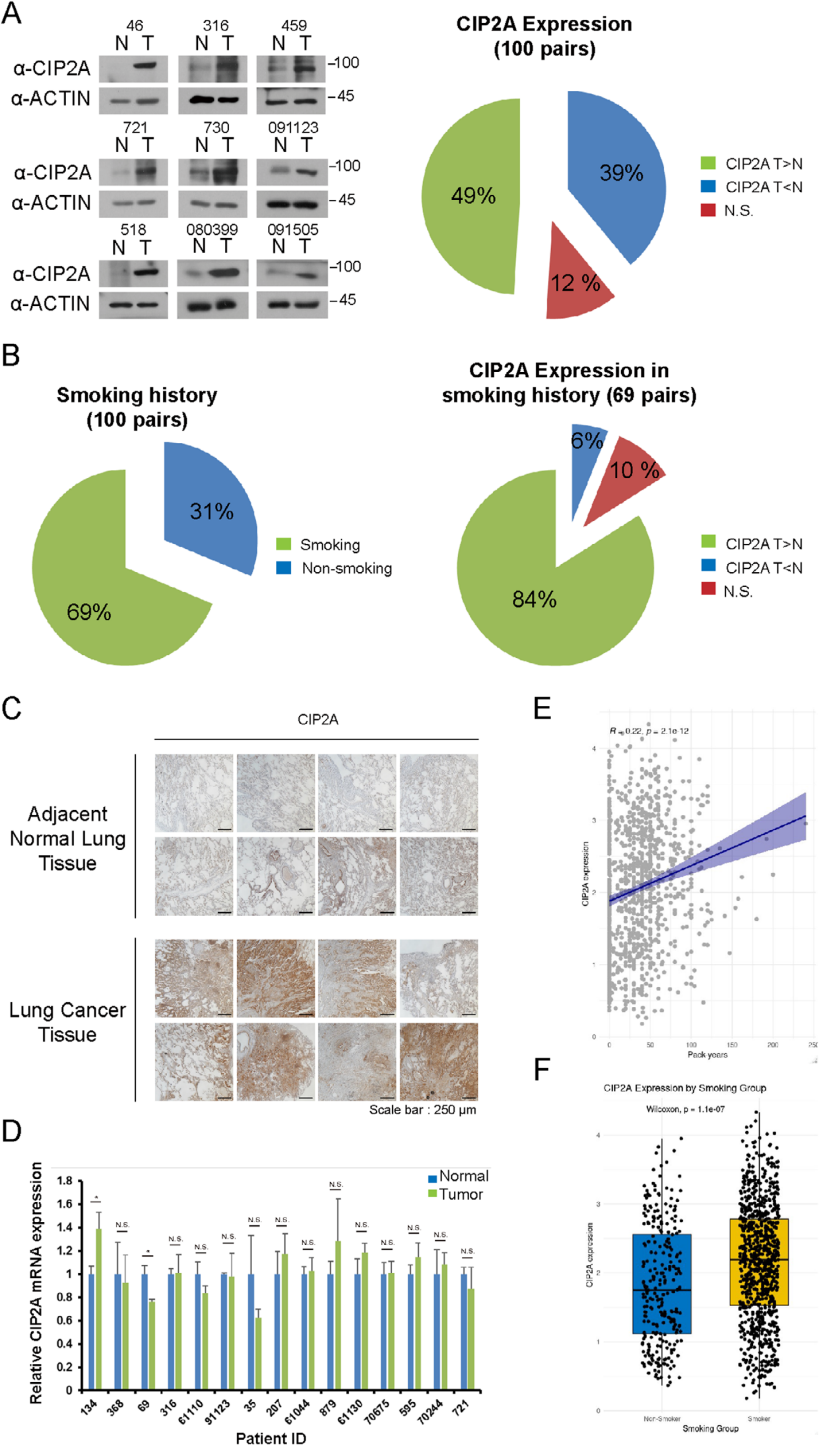
CIP2A is highly expressed in lung cancer tissues. (A) Representative western blot results in 100 paired adjacent normal lung tissues (*N*) and their lung cancer tissues (T). Each tissue was lysed by PRO‐PREP solutions and immunoblotted with the indicated antibodies. (B) The left chart shows the ratio of patients with or without a history of smoking. The right chart shows the proportion according to CIP2A expression pattern among patient samples with smoking history (69 pairs). (C) Representative IHC images on lung cancer tissues by CIP2A antibody were obtained by a light microscope at 40× magnification. (D) CIP2A mRNA levels in lung cancer tissues and adjacent normal tissues. (E) CIP2A expression was positively correlated with cumulative smoking exposure, measured in pack‐years (*R* = 0.22, *p* < 0.01). (F) CIP2A expression was significantly higher in smokers than in non‐smokers (Wilcoxon, *p* < 0.01).

To identify novel biomarkers for lung cancer, we analyzed the protein profiles of lung cancer tissues and adjacent normal tissues from patients using SILAC quantitative proteomics [67]. In this analysis, CIP2A showed a significant difference in abundance between lung cancer tissues and adjacent normal tissues. To investigate the expression and significance of CIP2A protein in lung cancer, we evaluated CIP2A protein levels between lung cancer and adjacent normal tissues from patients. Immunoblot assays were conducted on 100 pairs of lung cancer tissues, revealing high CIP2A expression in 49% of the lung cancer samples (Figure 1A and Figure S1) and the numbers and percentages according to other clinical parameters are summarized in Table S2. Since smoking is the main cause of lung cancer, we re‐analyzed the expression levels of CIP2A only in samples having smoking history. Among all patient sample pairs, 69% had a smoking history. In 84% of samples with a smoking history, CIP2A was highly expressed in tumor tissues (Figure 1B). Increased CIP2A protein levels were also confirmed through immunohistochemistry (Figure 1C). RT‐PCR analysis revealed no difference in CIP2A mRNA levels between the lung cancer tissues from patients and adjacent normal tissues (Figure 1D). Furthermore, when we performed an additional cohort analysis of The Cancer Genome Atlas (TCGA) lung cancer datasets, CIP2A expression demonstrated a significant positive correlation with cumulative pack‐years (Figure 1E), and was significantly elevated in patients with a smoking history compared with those without (Figure 1F). These findings suggest a relationship between CIP2A protein levels and lung cancer progression, with elevated CIP2A protein levels potentially due to post‐translational modification.

### 2.2 Knockdown of CIP2A Suppresses Proliferation, Migration, and Invasion Capacity of Lung Cancer Cells

**FIGURE 2:**
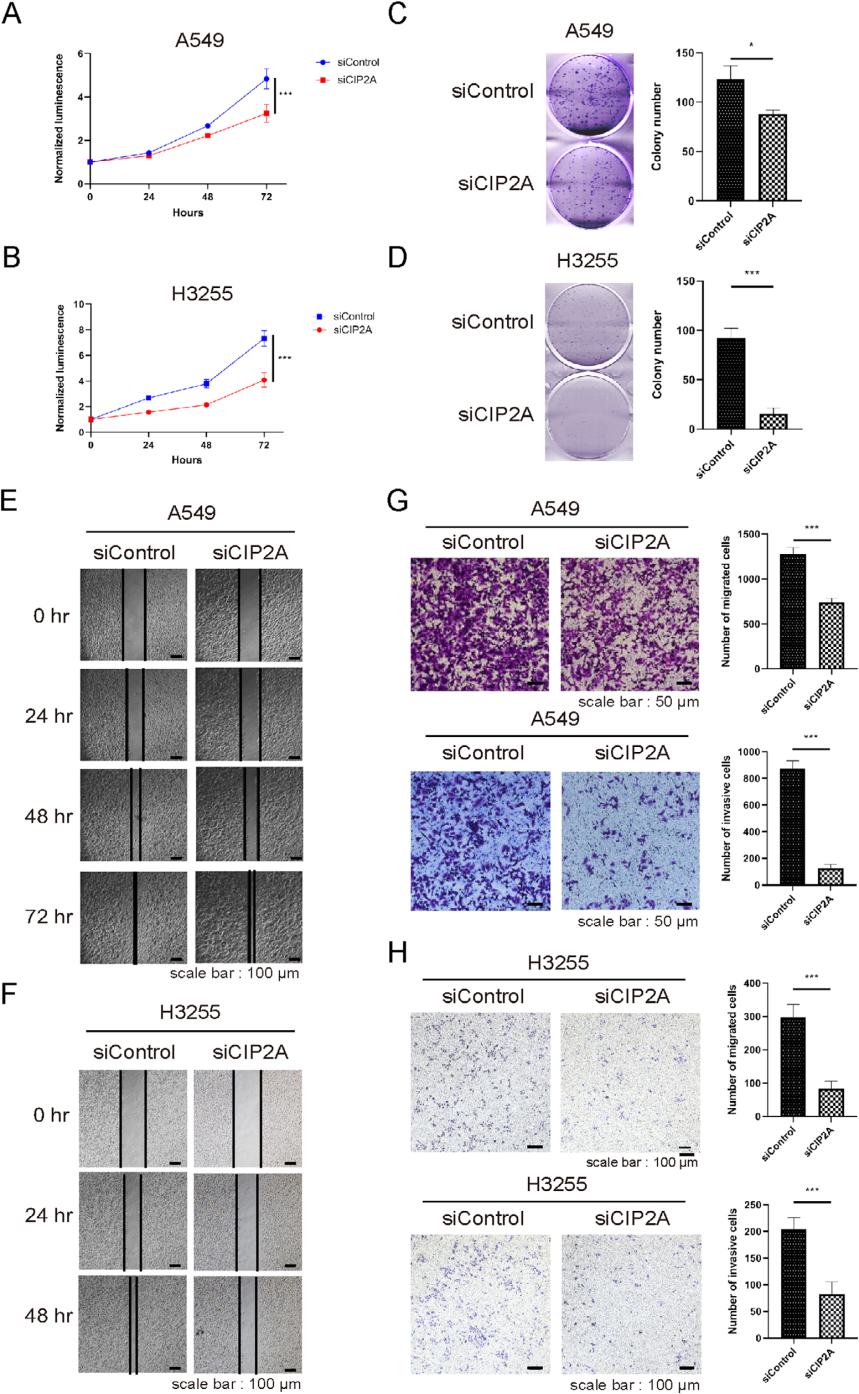
Knockdown of CIP2A suppresses proliferation, migration, and invasion capacity of lung cancer cells. (A,B) A549 cells and H3255 were seeded in 96‐well white‐bottom dishes and monitored over 3 days using a CellTiter‐Glo luminescence assay (*n* = 5, with normalization at Day 0). (C,D) A549 and H3255 cells were seeded in 6‐well plates and incubated for 7 days. Colonies were stained with crystal violet and counted. Data are expressed as mean ± SD from three independent experiments. (E,F) A549 and H3255 cells transfected with siRNAs were incubated until cells fully covered the bottom of 6‐well plates. Subsequently, the cells were wounded on Day 0 and cultured for indicated periods. (G,H) Migration (upper) and invasion (lower) assay of A549 cells and H3255 transfected with siCIP2A. Data are expressed as mean ± SD from five independent experiments. Statistical significance was determined using a two‐sided Student's *t*‐test. Images were obtained using a light microscope at 100× magnification. ***p < 0.001.

To investigate the role of CIP2A in lung tumorigenesis, several cell assays were conducted on CIP2A knockdown (KD) condition. For assessing cell growth, cell proliferation and colony‐forming assay were conducted in lung cancer cell line, A549 and H3255. CIP2A KD significantly reduced cell proliferation than the control group (Figure 2A–D). Furthermore, wound healing and transwell assay were implemented to estimate cell motility. The wound closure rate of CIP2A KD in A549 and H3255 cells was decreased than that of the control group (Figure 2E,F). Besides, CIP2A KD reduced the migration and invasion capacity of the two cell lines (Figure 2G,H). Collectively, these results demonstrated that high expression of CIP2A can promote cell growth and migration of lung cancer cells.

### 2.3 RING1 Interacts With CIP2A and Regulates Protein Expression by UPS

**FIGURE 3:**
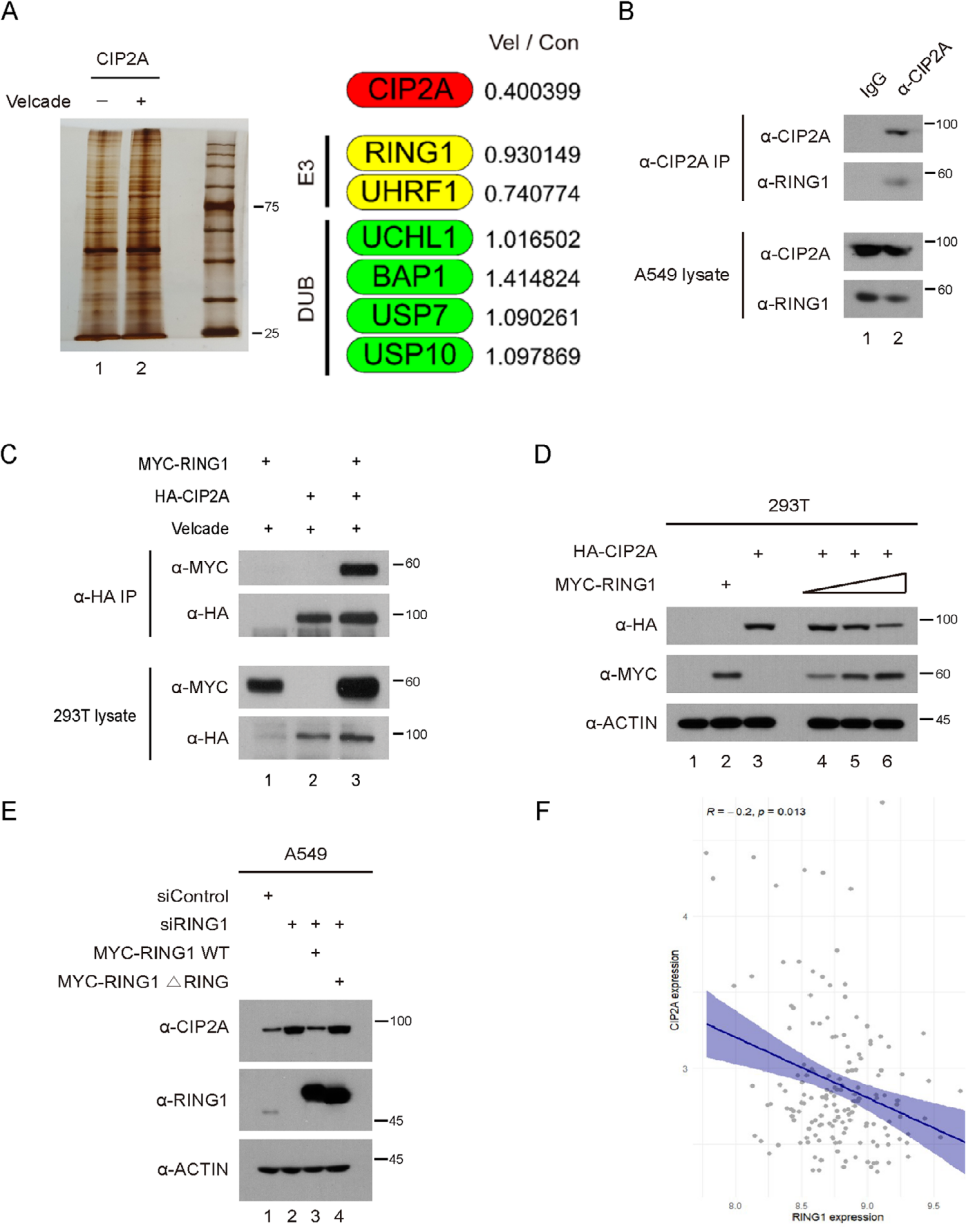
RING1 interacts with and regulates CIP2A. (A) HA‐tagged CIP2A‐overexpressed A549 cells were lysed and lysates were immunoprecipitated with anti‐HA beads. Immunoprecipitated complexes were separated in SDS‐PAGE and stained with silver staining kit. The stained bands were subjected to LC‐MS/MS analysis. The numbers in the right panel reflect the combined scores of all observed mass spectra that can be matched to amino acid sequences within that protein. M means protein marker lane. (B) A549 cell lysates were immunoprecipitated with either control IgG or CIP2A antibodies and A/G agarose beads. Total cell lysates and immune complexes were utilized for Western blot analysis. (C) HEK 293T cells were transfected with HA‐CIP2A and MYC‐RING1 plasmids. The cells were then lysed and immunoprecipitated with HA‐antibody conjugated beads. (D) HEK 293T cells were transfected with a fixed amount (2 µg) of HA‐CIP2A and a gradient (from 0.3 to 3 µg) of MYC‐RING1 constructs. After 48 h incubation, the cells were lysed and immunoblotted with the indicated antibodies. (E) A549 cells were transfected with siRNA against RING1 for 24 h, and then plasmids expressing RING1 wild‐type or RING domain (47–90 amino acid) truncated mutant form was transfected. After incubation, the cells were lysed and immunoblotted with the indicated antibodies. (F) The graph represents the correlation between CIP2A and RING1 expression among lung cancer cases in the TCGA database. CIP2A expression was negatively correlated with RING1 expression (*R* = −0.20, *p* = 0.013).

Based on the previous experiments, we hypothesized that CIP2A protein expression is regulated by post‐translational modification. Hence, immunoprecipitation (IP) mass spectrometry was performed to identify proteins related to UPS that interact with CIP2A, using A549 cell stably overexpressing CIP2A protein (Figure 3A and Table S3). Among the proteins immunoprecipitated with CIP2A antibodies, the E3 ligase RING1 was identified as a binding partner. Binding assays revealed that endogenous CIP2A and RING1 expression physically interacted (Figure 3B). Also, co‐IP experiments revealed a strong interaction between CIP2A and RING1 (Figure 3C). Next, HA‐CIP2A plasmid and increasing amounts of MYC‐RING1 plasmids were transfected into 293T cells. As the protein level of RING1 increased, CIP2A protein levels gradually decreased (Figure 3D). Since the RING domain is crucial for the E3 ligase activity of RING1, mutant RING1 which has no RING domain was generated and subjected to IP. Wild‐type (WT) RING1 effectively reduced CIP2A protein levels, whereas the RING1 domain‐deleted mutant RING did not (Figure 3E). In addition, analysis of the TCGA database revealed a negative correlation between RING1 and CIP2A expression, with higher RING1 levels associated with lower CIP2A expression (Figure 3F). These findings confirm the interaction between CIP2A and RING1 protein and suggest that RING1 may act as an E3 ligase for CIP2A.

**FIGURE 4:**
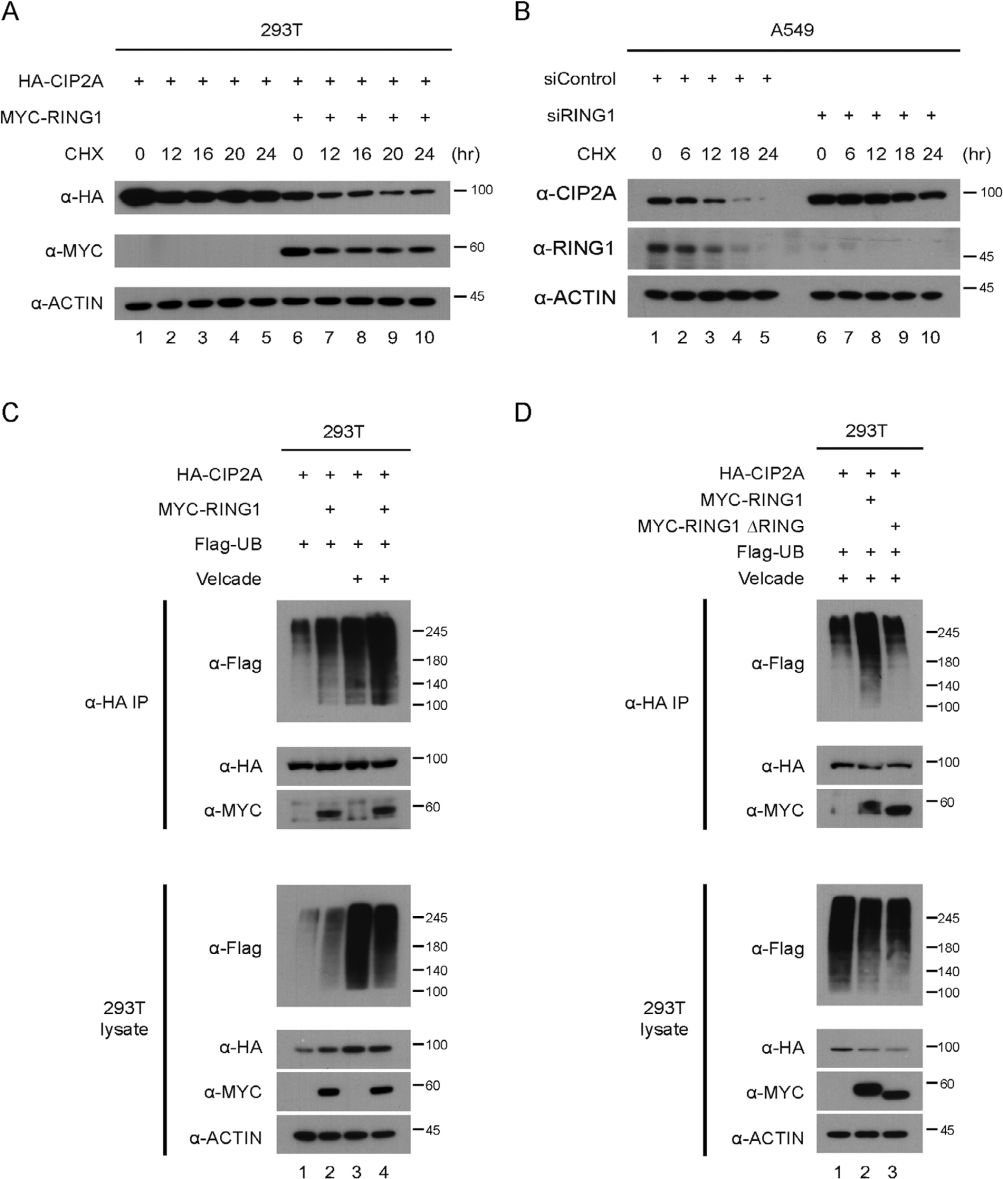
RING1 regulates CIP2A via ubiquitin proteasome system. (A,B) A549 cells were transfected with mixtures of plasmids or siRNA and incubated for 48 h followed by cycloheximide treatment. Cells were harvested at indicated period, and then subjected to the Western blot analysis. (C,D) HEK 293T cells were transfected with mixtures of plasmids expressing HA‐CIP2A, MYC‐RING1 WT, or RING domain truncated mutant and FLAG‐Ubiquitin for 48 h. The cells were treated with bortezomib for 6 h prior to harvesting. Cell lysates were immunoprecipitated with HA antibodies, and immune complexes were immunoblotted with the indicated antibodies.

Furthermore, the half‐life of CIP2A protein was reduced with RING1 overexpression (OE) (Figure 4A). Conversely, when RING1 levels were decreased using siRNA, the half‐life of CIP2A was increased (Figure 4B). Contrary to RING1, UHRF1, another E3 ligase candidate for CIP2A, did not affect CIP2A protein half‐life (Figure S2). To validate the E3 ligase function of RING1 on CIP2A protein, we also tested whether RING1 promotes CIP2A ubiquitination. Ubiquitin bands on CIP2A were increased in WT RING1‐trsnsfected cells (Figure 4C). In contrast, the mutant form of RING1 lacking the RING domain did not induce ubiquitination of CIP2A compared to that of the WT RING1 (Figure 4D). Overall, these findings demonstrate that RING1 regulates CIP2A protein levels through the UPS and its E3 ligase activity.

### 2.4 RING1 KD Increases the Proliferation and Migration of Lung Cancer Cells

**FIGURE 5:**
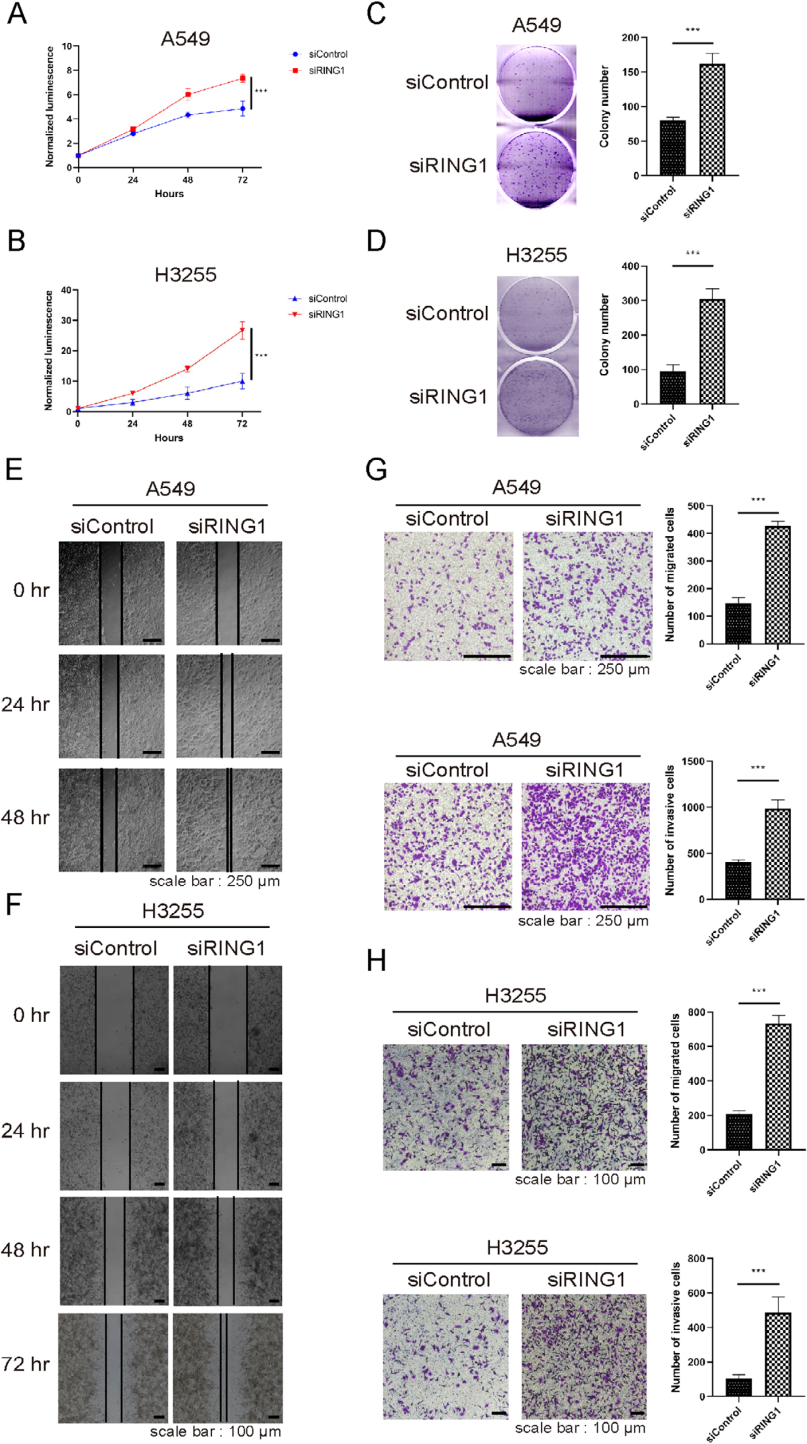
RING1 knockdown increases the proliferation and migration of lung cancer cells. (A,B) A549 and H3255 cells were seeded in 96 well‐white bottom dishes and monitored over 3 days using the CellTiter‐Glo luminescence assay (*n* = 5, with normalization at Day 0). (C,D) A549 and H3255 cells were seeded in 6‐well plates and incubated for 10 days. The cells were then stained with crystal violet dye and counted. Data are expressed as mean ± SD from three independent experiments. (E,F) A549 and H3255 cells transfected with siControl and siRING1 were seeded in 6‐well plates and incubated until cells fully covered the bottom. The cells were then wounded on Day 0 and cultured for the indicated period. (G,H) Migration (upper) and invasion (below) assay of A549 cells and H3255 transfected with siRING1. Data are expressed as mean ± SD from five independent experiments. Statistical significance was determined using a two‐sided Student's *t*‐test. Images were captured with a light microscope at 100× magnification. ***p < 0.001.

CIP2A is a recognized oncogene that promotes cell proliferation, evasion of senescence, and malignancy in various tumors by inhibiting PP2A activity. RING1 was hypothesized to potentially influence lung cancer progression through the regulation of CIP2A protein levels. RING1 KD promoted A549 and H3255 cell survival (Figure 5A,B), a finding further confirmed via the colony‐forming assay (Figure 5C,D). Moreover, wound‐healing and transwell assay revealed that RING1 KD significantly promoted the migration and invasion capacity of A549 and H3255 cells (Figure 5E–H).

To confirm that these effects resulted from increased CIP2A protein levels, A549 cells were transfected with siRNA targeting RING1 or/and CIP2A. While RING1 KD induced CIP2A protein levels, double KD of RING1 and CIP2A lowered both proteins expression (Figure S3A). In the cell proliferation and colony forming assay, RING1 depletion increased cell growth compared to that of siControl. However, when both CIP2A and RING1 siRNAs were treated, cell growth returned to normal levels (Figure S3B,C).

### 2.5 RING1 KD Leads to Increase of c‐MYC and Cyclin B1 Expression

**FIGURE 6:**
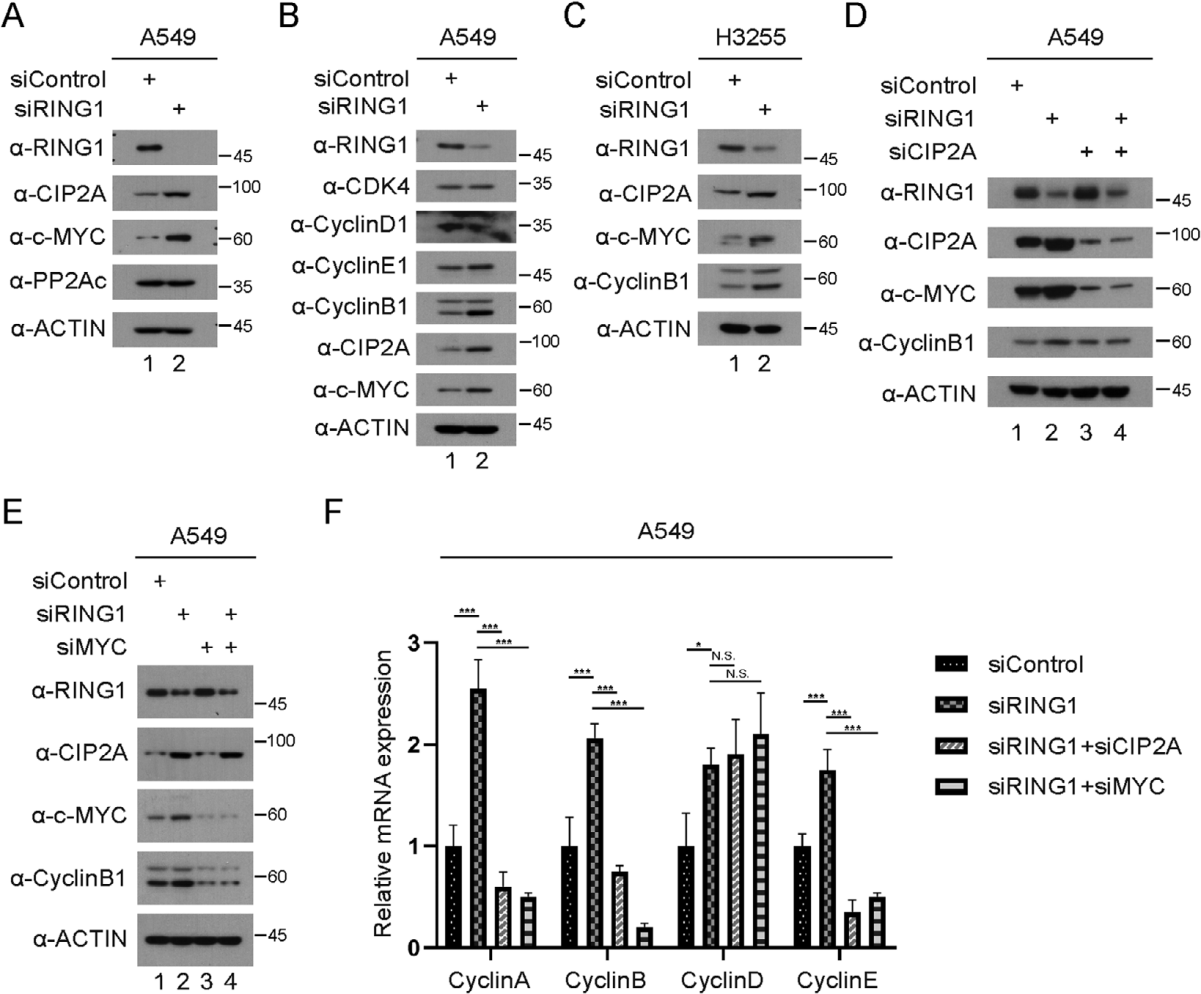
RING1 knockdown leads to increase of c‐MYC and Cyclin B1 expression. (A) A549 cells were seeded in 6‐well plates and transfected with siRING1. After incubation, cells were harvested and immunoblotted for the c‐Myc and PP2Ac. (B) A549 cells were seeded in 6‐well plates and transfected with siRING1. Cells were then harvested and immunoblotted for the cyclin proteins and CDK4 levels. (C) H3255 cells were seeded in 6‐well plates and transfected with siRING1. After incubation, cells were harvested and immunoblotted for the indicated antibodies. (D) A549 cells were seeded in 6‐well plates and transfected with indicated siRNAs. Cells were then lysed and immunoblotted by the indicated antibodies. (E) A549 cells were seeded in 6‐well plates and transfected with indicated siRNAs. Cells were then harvested and immunoblotted by the indicated antibodies. (F) A549 cells were seeded in 6‐well plates and transfected with siRNAs for RING1, CIP2A, and c‐Myc. RT‐PCR was conducted using primers for indicated human cyclin proteins. Ct value of each sample was calculated as a relative value based on that of the siControl sample. Error bars display SD value.

In several previous studies, CIP2A function as a oncogene through upregulation or activation of PP2A target proteins by suppressing PP2A activity in various cancer types. Consequently, PP2A inactivation by CIP2A predominantly occur through CIP2A binding on B56‐α subunit included in B subunit family of PP2A that regulate the stability of well‐known oncogenes like c‐MYC and E2F1. To verify whether these effects are occurred by RING1 KD, immunoblot assay was conducted for validating protein level of c‐MYC following siRING1 transfection in A549 cells. Interestingly, RING1 KD led to increase of c‐MYC, including CIP2A independently with PP2Ac expression (Figure 6A).

c‐MYC has been studied frequently as an oncogenic transcription factor that is contributed to unregulated cell cycle progression caused by elevated level of cyclin and cyclin‐dependent kinase proteins. A549 cells transfected with siRNA for RING1 were used for immunoblot assay to validate cyclin proteins. Among the several cyclin proteins, Cyclin B1 was significantly increased in RING1 knockdowned A549 cell lysates (Figure 6B) and similar result was gained from H3255 cells (Figure 6C). To verify that the elevated level of Cyclin B1 was resulted from CIP2A and c‐MYC upregulation, both proteins and RING1 were depleted by siRNA treatment simultaneously. As expected, siCIP2A successfully reduced Cyclin B1 level that was increased by RING1 KD (Figure 6D), and siRNA for c‐MYC showed similar effect as siCIP2A (Figure 6E). Nocodazole, reagent for G2/M phase arrest, was used to validate effect of changes in Cyclin B1 level on cell cycle progression. When the arrested cells were released, cells treated with siRING1 demonstrated that CyclinB1 protein level was higher than siControl group (Figure S4). Besides, mRNA expression of Cyclin A, B, and E, were also noticeably increased with RING1 KD, but when treated with siCIP2A or siMYC at once, the mRNA levels of the cyclin proteins were decreased again (Figure 6F).

Because CIP2A has been reported to regulate stability of c‐MYC protein by inhibiting PP2A activity, it had to be verified that downregulated level of c‐MYC and Cyclin B1 resulted from reduced CIP2A can be rescued by PP2A inhibition or KD. Therefore, okadaic acid, well‐known inhibitor of serine‐threonine phosphatase like protein phosphatase 1 (PP1) and PP2A, was utilized to evaluate the effect of PP2A on c‐MYC and Cyclin B1 expression. In conclusion, okadaic acid rescued the protein expression level of c‐MYC and Cyclin B1 downregulated by CIP2A KD (Figure S5A). Similarly, KD of catalytic subunit of PP2A successfully recovered the effect from decreased CIP2A (Figure S5B). Collectively, higher CIP2A expression followed by RING1 reduction in lung cancer cells increases cell proliferation by c‐MYC and Cyclin B1 upregulation through inhibition of PP2A activity.

### 2.6 NNK Reduces RING1 Expression Level Through DNMT1

**FIGURE 7:**
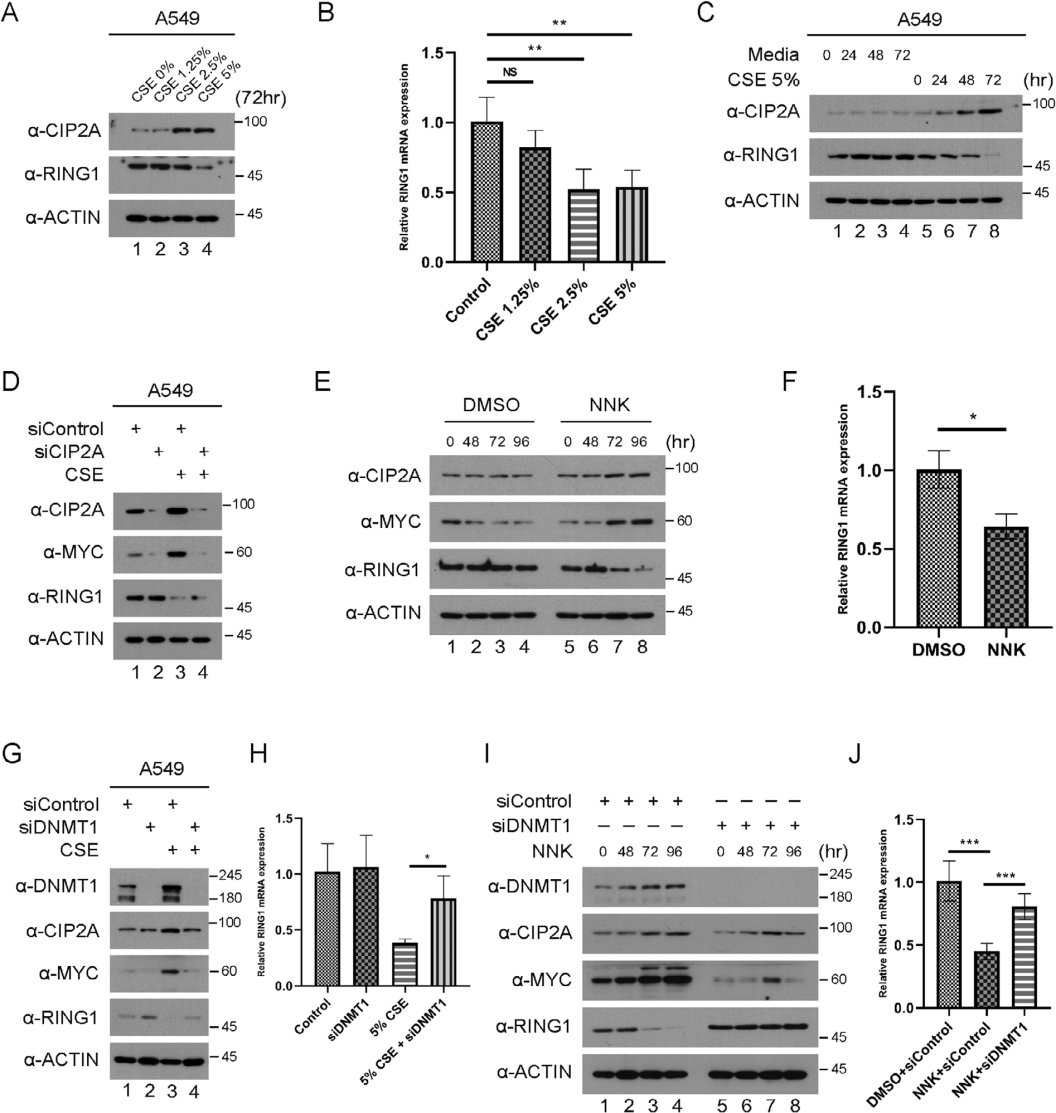
NNK reduces RING1 expression level through DNMT1. (A) A549 cells were treated with media (CSE 0%) or 1.25%, 2.5%, 5% of CSE and incubated for 72 h. Then cells were harvested and subjected to immunoblot using indicated antibodies. (B) mRNA expression of RING1 was evaluated by RT‐PCR and β‐actin, a house keeping gene, was used for an internal control. Ct values were calculated as a relative value based on the value of Control sample. (C) A549 cells were treated with media (CSE 0%) or 5% CSE and then incubated for indicated periods. The treated cells were harvested and subjected to immunoblot. (D) A549 cells were treated with CSE and siRNA for CIP2A. Then cells were harvested and subjected to immunoblot using indicated antibodies. (E) A549 cells were treated with DMSO or NNK and incubated for indicated periods (0, 48, 72, and 96 h). Then cells were harvested and subjected to immunoblot using indicated antibodies. (F) mRNA expression of RING1 was evaluated by RT‐PCR and β‐actin, a house keeping gene, was used for an internal control. Ct values were calculated as a relative value based on the value of DMSO‐treated sample. (G) A549 cells were treated with CSE and siRNA for DNMT1. Then cells were harvested and subjected to immunoblot using indicated antibodies. (H) mRNA expression of RING1 was evaluated by RT‐PCR. Ct values were calculated as a relative value based on the value of Control sample. (I) A549 cells were treated with siRNA for DNMT1 and NNK, and then incubated for indicated periods. The treated cells were harvested and subjected to immunoblot for the RING1, CIP2A, and c‐Myc levels. (J) mRNA expression of RING1 was evaluated by RT‐PCR. Ct values were calculated as a relative value based on the value of “DMSO + siControl” sample. Error bars display SD value. Statistical significance was determined using a two‐sided Student's *t*‐test, ***p < 0.001.

Data from previous experiments showed the roles of RING1 and CIP2A in lung cancer development. However, it could be hypothesized that, like other factors, such as genetic mutations that change normal cells into cancerous cells, there may be a trigger for RING1 downregulation to cause lung cancer. Cigarette smoking is a major contributor to its development, so we tried the CSE for validating this hypothesis. When we treated CSE from 0% to 5% of concentration to A549 cells, RING1 protein level was depleted in 5% CSE (Figure 7A). To figure out the reason for RING1 KD, RT‐PCR assay was conducted using CSE‐treated A549 cells. RING1 mRNA level downregulated by treatment of CSE 2.5% and 5% not 1.25% (Figure 7B). This effect of CSE on RING1 reduction and CIP2A induction also appeared time‐dependently (Figure 7C). Because CIP2A level was increased in CSE‐treated condition, we analyzed the c‐MYC level on the same condition. Along with CIP2A elevation, c‐MYC level was also upregulated in CSE‐treated condition. Moreover, CIP2A KD led to decrease of c‐MYC protein level increased by CSE treatment (Figure 7D).

NNK has been reported to play a key role in induction of lung cancer through DNA damage, activating α7 nicotine acetylcholine receptor and several signaling pathways, regulation of gene expression through DNA‐methyltransferase 1 (DNMT1), and immune suppression. Among these mechanisms, DNMT1 causes region‐specific hypermethylation of promoter CpG islands associated with diverse TSGs. So it is hypothesized that NNK downregulate RING1, as a TSG, in lung cancer progression due to cigarette smoking. At first, 20 µM NNK was treated in a time‐dependent manner and immunoblot assay was conducted to verify related protein levels. After 72 h of incubation with NNK, RING1 protein level was significantly reduced, in accordance with that, CIP2A and c‐MYC were upregulated (Figure 7E). To verify whether reduced RING1 protein level was resulted from suppression of mRNA transcription, RT‐PCR assay was conducted for RING1 mRNA. In fact, NNK treatment noticeably depleted RING1 mRNA level (Figure 7F).

Based on previous study, it was discovered that DNMT1 accumulation is modulated by NNK. NNK induces AKT activation, and promotes GSK3β phosphorylation at Ser9 to form inactive GSK3β. It leads to attenuating the ability of βTrCP, a specific DNMT1 E3 ligase; hence, DNMT1 expression in nucleus is upregulated. Moreover, AKT activation by NNK induce interaction between phosphorylated hnRNP‐U and βTrCP, which disrupts βTrCP/DNMT1 interaction. hnRNP‐U/βTrCP complex translocate to the cytoplasm, and it also leads to DNMT1 accumulation in the nucleus. These findings led to the hypothesis that DNMT1 may play an important role of CSE or NNK on regulation of RING1 expression.

To further investigate the role of the DNMT1 in the effect of CSE or NNK on RING1 depletion, siRNA for DNMT1 was used simultaneously with CSE treatment. Interestingly, protein level of RING1 downregulated by CSE was recovered by DNMT1 KD (Figure 7G). mRNA level of RING1 was rescued by siDNMT1, too (Figure 7H). These changes in RING1, CIP2A, and c‐MYC proteins were observed under NNK treatment and DNMT1 KD conditions (Figure 7I). Moreover, the diminished mRNA expression of RING1 following NNK treatment was restored by siDNMT1, aligning with the protein levels (Figure 7J). These results demonstrated that RING1 reduction is caused by DNMT1 in the development of lung cancer caused by NNK.

### 2.7 Relationship Between RING1 Protein, Smoking Exposure, and Lung Cancer Growth in Xenograft Models

**FIGURE 8:**
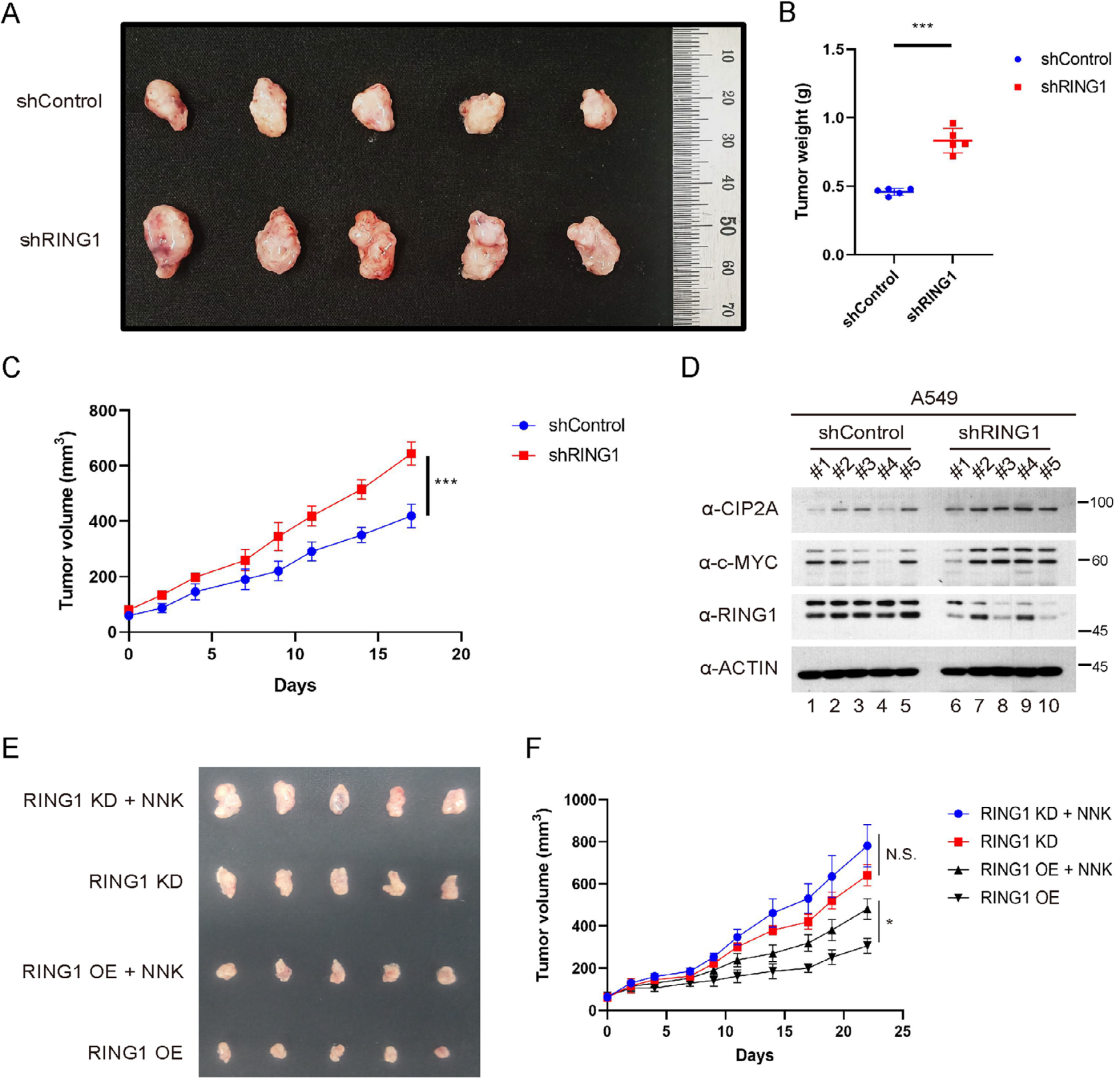
Relationship between RING1 protein, smoking exposure, and lung cancer growth in xenograft models. (A–C) Effect of RING1 knockdown in xenograft mice. Tumor size was estimated using caliper at an indicated time point. After surgical excision, each tumor mass was weighed on an electronic scale. (D) Western blot result of tumor masses of xenograft mice using stable RING1‐knockdowned A549 cell lines. (E,F) Xenograft tumors generated from A549 cells with stable RING1 knockdown (KD) and overexpression (OE) were compared between mice treated with NNK for 2 weeks and untreated controls. Tumor size was estimated using caliper at an indicated time point. Statistical significance was determined using a two‐sided Student's *t*‐test. ***p < 0.001.

Next, we sought to validate in vivo the effects of RING1 and the smoke carcinogen that were observed in vitro. In order to validate these effects on mouse model, we established A549 stable cell lines, which express shRNA for RING1. Along with the in vitro results, stable RING1 KD significantly promoted the tumor growth in xenograft model (Figure 8A) and also tumor volume and tumor weight were increased than shControl groups. (Figure 8B,C). In addition, western blot was performed on tumor mass extracted from a xenograft model using A549 cells expressing shRING1, and similar to the results at the cell level, a tendency for CIP2A and c‐MYC to increase due to RING1 KD was confirmed (Figure 8D).

To evaluate the potential impact of smoking exposure in the context of animal model, we performed xenografts generated with stably RING1 knockdowned or overexpressed A549 cells, and mice were treated with NNK for 2 weeks. As shown in Figure 8E,F, there was no significant difference between the RING1 KD + NNK group and the RING1 KD group. In contrast, a significant difference was observed between the RING1 OE + NNK group and the RING1 OE group. These results are consistent with the notion that the tumor‐promoting effect of NNK in lung cancer progression is mediated, at least in part, through suppression of RING1 expression.

## 3 Discussion

Lung cancer is a deadly disease affecting millions of people globally. The development and progression of lung cancer involve the dysregulation of several molecular pathways, including those involving CIP2A, RING1, and DNMT1 proteins. This study aimed to investigate the roles of these proteins in lung cancer and identify potential therapeutic targets for treatment. CIP2A is an oncogenic protein that plays a critical role in the development and progression of several types of cancer, including lung cancer. High expression of CIP2A is associated with a high incidence of cancer and a poor prognosis. Results in this study revealed that CIP2A expression is frequently upregulated in lung cancer tissues. It was also found that CIP2A promotes lung cancer cell proliferation by inhibiting the activity of PP2A, a tumor suppressor that regulates multiple signaling pathways involved in cell growth and apoptosis. Moreover, we discovered that the E3 ubiquitin ligase, RING1, plays an important role in regulating the protein stability of CIP2A in lung cancer formation. CIP2A expression was regulated by RING1 and its activity affected the oncogenic capacity of lung cancer cells. Consequently, these results were coming through PP2A, c‐MYC, and Cyclin B1 axis.

RING1, an E3 ligase in UPS, is a member of the polycomb repressive complex 1 (PRC1), which plays a critical role in the regulation of gene expression during development and differentiation. PRC1 is a complex that regulates chromatin structure through histone ubiquitination and is consists of Polycomb (PRC), polyhometic (PH), Bmi1, RING1 (RING1a), and RING1b/RNF2. Especially, RING1 works together with RING1b/RNF2 to provide the E3 ligase activity of PRC1, its mono‐ubiquitination activity on histone. While RING1b/RNF2 has been reported to be involved in cancer development and autophagy through poly‐ubiquitination of p53 and AMBRA1 in addition to its mono‐ubiquitination function, there have been no studies on the poly‐ubiquitination function of RING1 [68]. This study revealed that RING1 regulates CIP2A protein level through poly‐ubiquitination. It was also found that RING1 KD promotes lung cancer cell proliferation by upregulating CIP2A expression followed by increase of c‐MYC, and Cyclin B1. In this study, we demonstrated for the first time that RING1 is involved in the poly‐ubiquitination of CIP2A, in addition to its role as a component of PRC1 for histone mono‐ubiquitination.

In Figure 1, 69% of the 100 patient groups had a smoking history. CIP2A expression was confirmed to be higher in 84% of cancer tissues than in surrounding normal tissues among 69 paired patient tissues. This finding confirmed the strong association between smoking and lung cancer, as established in numerous studies. Furthermore, a critical mechanism or molecule related to smoking may be responsible for elevating CIP2A expression levels. For this purpose, we showed that the expression of RING1, an E3 ligase that regulates the protein stability of CIP2A, is affected by CSE, particularly NNK. Moreover, we demonstrated that this NNK‐mediated expression of RING1 is regulated through DNMT1. In case of chronic obstructive pulmonary disease (COPD), which is often considered a precursor to lung cancer, smoking, which can be influenced by substances such as reactive oxygen species (ROS), lipopolysaccharide (LPS), and NNK is a primary cause. This study revealed that NNK exposure enhances DNMT1 activity and consequently suppresses the transcription of RING1 in lung cancer cells. The findings from this study has provided a molecular mechanism for how cigarette smoking might influence lung cancer development and offered a new direction for investigating the association between smoking and lung cancer.

In addition to E3 ligases to control CIP2A protein level, our LC‐MS/MS experiments also revealed potential deubiquitinase enzymes (DUBs) candidate proteins that may interact with CIP2A. From the results presented in Figure 3, various DUBs were identified in addition to the E3 ligases RING1 and UHRF1. UCHL1 and BAP1 belong to the UCHL family, while USP7 and USP10 are classified under the USP family, both of which are well‐studied groups of DUBs. Notably, BAP1 exhibits deubiquitinating activity toward a range of substrates, including histones, and is recognized as a multifunctional protein in various cancers such as melanoma [69]. USP10 also demonstrates a dual role depending on the cancer type; it has been known to act as a tumor suppressor in gastric carcinoma, hepatocellular carcinoma, and intestinal adenocarcinoma, but function as oncogenic function in prostate cancer, breast cancer, and acute myeloid leukemia [70]. Therefore, further investigation into the role of DUB proteins in relation to CIP2A could provide deeper insights into the function of CIP2A in the pathogenesis of lung cancer and identifying DUB could significantly contribute to the development of CIP2A inhibitors as a therapeutic target in cancer.

In conclusion, this study highlights the critical roles of CIP2A, RING1, and DNMT1 in the development and progression of lung cancer. Targeting these proteins may represent promising therapeutic strategies for treating lung cancer and optimizing predictive biomarkers for patient selection. In addition, future studies should focus on elucidating the molecular mechanisms underlying the dysregulation of these proteins in lung cancer. Public health interventions aimed at preventing tobacco use and promoting smoking cessation should continue to be a priority to reduce the incidence of lung cancer.

## 4 Materials and Methods

### 4.1 Antibodies and Reagents

The primary antibodies were purchased as follows: anti‐CIP2A (#SC‐80659, Santa Cruz Biotechnology), anti‐RING1 (#13069, Cell Signaling Technologies), anti‐β‐Actin (#SC‐47778, Santa Cruz Biotechnology), anti‐CDK4 (#12790, Cell Signaling Technologies), anti‐CyclinD (#2978, Cell Signaling Technologies), anti‐CyclinE (#4129, Cell Signaling Technologies), anti‐CyclinB1 (#SC‐752, Santa Cruz Biotechnology), anti‐c‐MYC (#SC‐42, Santa Cruz Biotechnology), anti‐PP2Ac (#2038, Cell Signaling Technologies), anti‐MYC tag (#SC‐40, Santa Cruz Biotechnology), anti‐HA tag (#MMS‐101P, Covance), anti‐FLAG tag (#2368, Cell Signaling Technologies), anti‐E2F1 (#SC‐56661, Santa Cruz Biotechnology), anti‐DNMT1 (#5032, Cell Signaling Technologies). Sequences of siRNAs, shRNAs, and primers are described in the Table S1.

Cycloheximide and Velcade were purchased from Sigma‐Aldrich. Lipofectamine RNAiMAX was purchased from Invitrogen. EASY‐BLUE and PRO‐PREP solutions were purchased from iNtRON biotechnology. VECTASTAIN universal Elite ABC kit (#PK‐6200, Vector Laboratories) and VECTASTAIN mounting medium were purchased from Vector Lab.

### 4.2 Cigarette Smoke Extract

A standing tube containing DMEM was placed in an Erlenmeyer flask with a branch. The flask was then sealed with a rubber stopper, which had a sterilized Pasteur pipette inserted into it. A rubber hose was connected to the Pasteur pipette, and a Research‐grade cigarette (3R4F), without a filter, was inserted into the rubber hose. After connecting another hose to the motor and branch of the Erlenmeyer flask, the cigarette was lit. DMEM was removed from the Erlenmeyer flask, filtered once through a syringe filter, mixed with FBS, and treated to cells. One cigarette per 10 mL of DMEM was considered 100% cigarette smoker extract.

### 4.3 Statistical Analysis

The data are expressed as mean and SD to indicate variability. Statistical analyses were conducted using an unpaired Student's *t*‐test for cell assays. The Student's *t*‐test was used to analyze data in the graphs for comparisons between two groups to determine statistical significance. *p* value < 0.05 was considered significant.

## Author Contributions

I.‐h. J. and P.C.‐W.L. designed and performed the experiments and prepared the manuscript. J.K.Y., J.‐O.J. and G.D.L. assisted the experiments and data analysis. L.P.C.‐W.L. conducted and analyzed the LC–MS/MS‐based experiments and consulted the data analysis. J.‐O.J. and G.D.L. advised the project and revised the manuscript. All authors read and approved the final manuscript.

## Funding

This study was supported by the National Research Foundation of Korea (NRF) grant funded by the Korean government (MIST) (RS‐2023‐00208173, RS‐2023‐00207868) and by a grant of the Korea Health Technology R&D Project through the Korea Health Industry Development Institute (KHIDI), funded by the Ministry of Health & Welfare (RS‐2024‐00437312). This research was also supported by the Asan Institute for Life Sciences, Seoul, Republic of Korea (2023IP0114).

## Ethics Statement

*Tissue samples from patients*: Western blot and immunohistochemistry data on tissue samples were obtained from patients with lung cancer who participated in this study. All experimental protocols were approved by the Institutional Review Board of Asan Medical Center and University of Ulsan College of Medicine (2014‐0960). Human lung tissues were obtained from the Asan Bio Resource Center (2014‐2089).

*Animal studies*: All mice were bred at the animal facilities of the Asan Institute for Life Sciences, University of Ulsan College of Medicine. All animal procedures were approved by the Institutional Ethics Committee and Institutional Animal Care Committee of University of Ulsan College of Medicine (2022‐12‐296).

## Conflicts of Interest

The authors declare no conflicts of interest.

## Supporting information




**Table S1**: siRNA, shRNA, and RT‐PCR primer sequence
**Supplementary Table 2**: CIP2A expression pattern analysis in several parameters. Analysis of CIP2A protein expression in 100 paired lung cancer tissues, summarized by clinical parameters with the number of cases and percentages.
**Supplementary Table 3**: Mass proteomic analysis_CIP2A.
**Supplementary Figure 1**: The protein levels of CIP2A in lung cancer patients’ normal and tumor tissues. (A) Western blot results in 100 paired adjacent normal lung tissues (N) and tumor tissues (T). The numbers on each blot are the serial number for patient identification.
**Supplementary Figure 2**: The effect of UHRF1 knockdown on the half‐life of CIP2A protein. A549 cells were transfected with mixtures of plasmids or siRNA and incubated for 72 h followed by cycloheximide treatment. Cells were harvested at indicated period, and then subjected to the Western blot analysis.
**Supplementary Figure 3**: The effect of RING1 and CIP2A knockdown on lung cancer cell growth. **(A)** A549 cells were seeded in 6‐well plates and transfected with siRING1 and siCIP2A. After incubation, cells were harvested and immunoblotted for the indicated antibodies. **(B)** A549 cells were seeded in 96 well‐white bottom dishes and monitored over 3 days using the CellTiter‐Glo luminescence assay (n = 5, with normalization at day 0). **(C)** A549 cells were seeded in 6‐well plates and incubated for 7 days. The cells were then stained with crystal violet dye and counted. Data are expressed as mean ± SD from three independent experiments.
**Supplementary Figure 4**: The effect of RING1 knockdown on the cell cycle progression **(A)** A549 cells were transfected with siRNA against RING1 and then nocodazole was treated. After incubation, the media was replaced with fresh complete media for releasing cell cycle progression. Then cells were incubated for indicated periods and harvested for following Western blot analysis.
**Supplementary Figure 5**: The effect of CIP2A knockdown and PP2A actitvity on c‐Myc protein level. **(A)** A549 cells were transfected with siRNA for CIP2A and then 10 nM of okadaic acid was treated and incubated for 24 h. Cells were harvested and subjected to immunoblotting for the c‐Myc and cyclin B1 levels. **(B)** A549 cells were transfected with siRNA for CIP2A and PP2Ac. Cells were harvested and subjected to immunoblotting for the c‐Myc and cyclin B1 levels.
**Supplementary Figure 6**: DNMT1 and RING1 protein level in tissues of lung cancer patients. **(A‐B)** Western blot results in 91 paired adjacent normal lung tissues (N) and tumor tissues (T). Each tissue was lysed by PRO‐PREPTM solutions and immunoblotted with the indicated antibodies. **(C‐D)** Chart illustrates the percentage distribution of DNMT1 and RING1 protein expression levels between tumor tissues and adjacent normal tissues.
**Supplementary Figure 7**: Transcriptomic profiling of RING1‐overexpressing (OeR) and RING1‐knockdown **(KdR) A549 cells**. we performed transcriptomic analysis using Affymetrix Human Gene ST 2.0 arrays in A549 cells with RING1 overexpression (OeR) and siRNA‐mediated knockdown (KdR). **(A)** Volcano plot of OeR vs Control showing robust induction of interferon/antiviral genes, with RING1 highlighted. **(B)** Scatter plot of comparing OeR vs KdR. RING1 fold changes; overexpression showed significant effects, whereas knockdown showed more subtle changes. **(C)** Heatmap of the top 25 significant genes in OeR (FDR ≤ 0.05), revealing strong enrichment of immune/antiviral pathways.

## Data Availability

Data are available upon reasonable request and raw data of MS result are provided in Supporting Information.
