# Supplementary material for: Ring Finger Protein 1, a Novel Ubiquitin E3 Ligase Targeting Cancerous Inhibitor of Protein Phosphatase 2A to Suppress Smoking‐Induced Lung Tumorigenesis
Source: MedComm (2020). 2025 Dec 7;6(12):e70522. doi: 10.1002/mco2.70522 (PMC12681960; doi:10.1002/mco2.70522)
Supplement: Supplementary file 1 — Table S1: siRNA, shRNA, and RT‐PCR primer sequence Supplementary Table 2: CIP2A expression pattern analysis in several parameters. Analysis of CIP2A protein expression in 100 paired lung cancer tissues, summarized by clinical parameters with the number of cases and percentages. Supplementary Table 3: Mass proteomic analysis_CIP2A. Supplementary Figure 1: The protein levels of CIP2A in lung cancer patients’ normal and tumor tissues. (A) Western blot results in 100 paired adjacent normal lung tissues (N) and tumor tissues (T). The numbers on each blot are the serial number for patient identification. Supplementary Figure 2: The effect of UHRF1 knockdown on the half‐life of CIP2A protein. A549 cells were transfected with mixtures of plasmids or siRNA and incubated for 72 h followed by cycloheximide treatment. Cells were harvested at indicated period, and then subjected to the Western blot analysis. Supplementary Figure 3: The effect of RING1 and CIP2A knockdown on lung cancer cell growth. (A) A549 cells were seeded in 6‐well plates and transfected with siRING1 and siCIP2A. After incubation, cells were harvested and immunoblotted for the indicated antibodies. (B) A549 cells were seeded in 96 well‐white bottom dishes and monitored over 3 days using the CellTiter‐Glo luminescence assay (n = 5, with normalization at day 0). (C) A549 cells were seeded in 6‐well plates and incubated for 7 days. The cells were then stained with crystal violet dye and counted. Data are expressed as mean ± SD from three independent experiments. Supplementary Figure 4: The effect of RING1 knockdown on the cell cycle progression (A) A549 cells were transfected with siRNA against RING1 and then nocodazole was treated. After incubation, the media was replaced with fresh complete media for releasing cell cycle progression. Then cells were incubated for indicated periods and harvested for following Western blot analysis. Supplementary Figure 5: The effect of CIP2A knockdown and PP2A actitvity on c‐My [file MCO2-6-e70522-s001.docx]

**Supplementary Information**

Ring finger protein 1 (RING1), a novel Ubiquitin E3 ligase targeting Cancerous inhibitor of protein phosphatase 2A (CIP2A) to suppress smoking-induced lung tumorigenesis

**In-ho Jeong, Jae Kwang Yun, Jun-O Jin, Geun Dong Lee, and Peter Chang-Whan Lee**

# Material and Methods

#### *Cell culture*

A549, H3255, and HEK 293T cells were obtained from American Type Culture Collection (ATCC). A549 and HEK 293T cells were maintained in Dulbecco’s Modified Eagle’s Medium (DMEM) supplemented with 10% fetal bovine serum (FBS) and 1% penicillin/streptomycin (P/S). H3255 cells were cultured in Roswell Park Memorial Institute (RPMI) 1640 medium supplemented with 10% FBS and 1% P/S. All cells were maintained in a humidified atmosphere of 5% CO2 incubator at 37°C.

#### *Plasmid and siRNA transfection*

Plasmids (HA-CIP2A, MYC-RING1 WT / ∆RING, FLAG-UB) were utilized for transient protein overexpression, and iNfect reagents were mixed with each plasmid for transfection. Briefly, cells were seeded at 1 × 105 per 6-well culture plate and incubated for 6 h. Plasmid DNAs were mixed with the iNfect reagent in OptiMEM and incubated for 15 min. After treatment, culture media was replaced by complete media. After 48 h incubation, cells were lysed and then subjected to

western blot analysis with indicated antibodies.

For transient knockdown of the target proteins, LipofectamineTM RNAiMAX was used according to the manufacturer's protocol. Small interfering RNAs (siRNAs) were synthesized from Genolution (Seoul, South Korea). Briefly, cells were seeded at 1 × 105 per 6-well culture plates and then incubated for 6 h. siRNA was mixed with RNAiMAX reagent in OptiMEM and incubated for 15 min, and then the lipoplex was added to cell culture plates. At 72 h post-transfection, cells were harvested and lysed and then the cell lysates were subjected to western blot analysis for knockdown control with indicated antibodies.

#### *Mass spectrometry (MS)*

For MS proteomic analysis, retroviral vectors were utilized to express HA tagged CIP2A in A549 cells. Briefly, A549 cells were incubated in 150 mm cell culture dishes and treated with 125 mM di-tert-butyl peroxide (DTBP) for 10 min and neutralized by 2.5M glycine for 5 min before being lysed in 4 ml of lysis buffer with protease inhibitors. Cleared lysates were filtered through 0.45 μm spin filters and immunoprecipitated with 30 μl of anti-HA antibody-conjugated agarose beads. Complexes were washed with lysis buffer, separated in SDS-PAGE, and stained with a silver staining kit. The complexes were exchanged into PBS, eluted with HA peptide, and precipitated with 10% trichloroacetic acid. The complexes were analyzed on a Thermo Orbitrap-XL mass spectrometer.

#### *Immunoblotting*

Human tissues were homogenized and lysed using PRO-PREPTM. Samples were centrifugated at 17,000 × g for 30 min at 4°C, then the supernatants were analyzed by BCA kit and heat-incubated with sample buffer for the following procedure. 30 μg of protein extract was separated by SDS-PAGE and analyzed by western blot using primary antibodies and corresponding IgG HRP-conjugated secondary antibodies. Enhanced chemiluminescence was used to detect targeted proteins. Cultured cells were washed with cold PBS and then lysed with 1% SDS-lysis buffer (50 mM Tris pH 7.4, 150 mM NaCl, 1% SDS, 1 mM EDTA). Protein concentration was measured by BCA assay kit, and the lysates were heat-incubated with 5× sample buffer (250 mM Tris-Cl pH 6.8, 25% glycerol, 2% SDS, 0.1% bromophenol blue, 5% β-mercaptoethanol). 20 μg of protein

was resolved by SDS-PAGE and the detection procedure was the same as human tissue samples.

#### *Immunoprecipitation*

For co-immunoprecipitation (Co-IP), plasmids-transfected HEK 293T cells were harvested at 2,400 rpm for 5 min at 4°C and washed by cold PBS. Then cell pellets were suspended in 500 µl of MCLB-lysis buffer (50 mM Tris pH 7.4, 150 mM NaCl, 0.5% Nonidet P40, 1 mM EDTA) supplemented with protease inhibitors. The cell resuspensions were homogenized by 21G syringe 25 times and centrifuged at 17,000 × g for 30 min at 4°C. The supernatant was incubated with the anti-HA

antibody-conjugated agarose beads at 4°C overnight. After incubation, the suspensions were washed 5 times with 1 ml

MCLB-lysis buffer and then subjected to immunoblotting with the indicated antibodies. For endogenous immunoprecipitation, A549 cells were harvested at 2,400 rpm for 5 min at 4°C, washed with cold PBS, and resuspended in 500 µl of MCLB-lysis buffer supplemented with protease inhibitors. The cell resuspensions were homogenized by syringe and incubated for 30 min at 4°C using a tube rotator. After centrifuging at 17,000 × g for 30 min at 4°C, the supernatant was incubated with the anti-CIP2A antibody and pulled down with Protein A/G agarose beads. The immune complexes were washed 5 times with 1 ml of lysis buffer and then subjected to immunoblotting with the indicated antibodies.

#### *Quantitative reverse transcription PCR (qRT-PCR)*

For analysis of mRNA expression, RNA was isolated by EASY-BLUETM solution according to the manufacturer’s instructions. cDNA was synthesized using Maxime RT PreMix using oligo dT primer from 1 μg of RNA. qRT-PCR was conducted using SYBR Green I Master Mix on a CFX Connect Real-time PCR system (Bio-Rad). Each Ct value of target genes was normalized to the Ct value of human β-actin.

#### *Cell proliferation assay*

Cell proliferation was measured by the CellTiter-Glo Luminescent Cell Viability Assay Kit. Briefly, cells were seeded with

1 × 104 cells per 96-well white bottom culture plates. Plates and CellTiter-Glo reagent were equilibrated at room temperature for 30 min before treatment. 100 µl of reagent was added and mixed for 5 min on an orbital shaker. Subsequently, the plate was incubated at room temperature for 10 min. Analysis was conducted every 24 h from day 0 to day 3 and luminescence intensity at 420 nm was determined using a GloMax® 96 Microplate Luminometer (Promega).

#### *Colony formation assay*

Transfected cells were seeded at 1 × 103 cells per well in 6-well culture plates and incubated in each media for 7 ~ 10 days. The cells were then fixed with 4% paraformaldehyde and stained with 0.5% crystal violet resolved in methanol for 30 min.

#### *Wound healing assay*

Transfected cells were seeded at 5 × 105 cells per well in 6-well culture plates and incubated for 1 or 2 days. Cell monolayers were scratched with a sterile 200 μl pipette tip and washed with medium to remove detached cells. The wounded area was observed through a light microscope after washing once a day.

#### *Transwell migration / invasion assays*

For the migration assay, cells were seeded at 5 × 103 cells per well in the upper chamber of a transwell insert placed in a 24-well plate and filled with DMEM without FBS. The lower chamber was filled with DMEM supplemented with 10% FBS and incubated for 12 h. Migrated cells were fixed with 4% paraformaldehyde/sucrose and stained with 0.5% crystal violet for 5 min. Cells remaining at the upper surface of the insert were removed with a wet cotton swab, and the migrated cells were counted in five random fields under light microscopy. For the invasion assay, cells were seeded at 1 × 104 cells per well in

the upper chamber of a transwell insert coated with 1 mg of Matrigel® Matrix. The lower chamber was filled with 800 μl DMEM supplemented with 10% FBS and incubated for 24 h. Invaded cells were fixed and stained by the same method mentioned above. Cells remaining at the upper surface of the insert were removed, and the invaded cells were counted in five random fields under light microscopy. Cell counts are expressed as mean ± standard deviation (SD).

#### *Cell synchronization*

A549 cells were treated with 0.1 µg/ml nocodazole for 18 h. A549 cells arrested in the G2/M phase were washed with PBS

three times, and then released by exchanging to a complete medium. Cells were harvested at 0, 6, 12, 18 and 24 hr after releasing and harvested for Western blot analysis.

#### *Immunohistochemistry (IHC) staining*

Paraffin-embedded sections of human lung tumor and adjacent normal tissues were deparaffinized in xylene two times and

rehydrated in a gradient ethanol concentration. Antigen retrieval was subsequently performed by boiling the samples for 15

min in sodium citrate buffer (pH 6.0). According to the manufacturer's protocol, the section was stained using the

VECTASTAIN universal Elite® ABC kit. Briefly, the sections were blocked for 1 h and then incubated with anti-CIP2A

antibody (1:100) at 4°C overnight followed by 15 min incubation with 3% H_2_O_2_ solution to inactivate endogenous peroxidase

After washing three times with TBS-T, the sections were incubated with biotinylated secondary antibody solution for 1 h and

then incubated with ABC reagent. Detection was developed with 3,3' diaminobenzidine (DAB), and the nuclei were

counterstained with Mayer's hematoxylin solution. The specimen was mounted using a Vectashield® mounting medium, and

then images were obtained using a light microscope.

#### *Manufacture of short-hairpin RNAs (shRNAs)*

To prepare the constructs of shRNAs, the target-specific oligomers were designed and purchased from Macrogen (Seoul

South Korea). Oligos for annealing were incubated for 10 min at 70°C and then slowly cooled to room temperature. The

annealed oligos were cloned into pLKO.1 vector (digested by AgeΙ and EcoRΙ) using T4 DNA ligase. shRNA clones were

verified by nucleotide sequencing and digestion products.

#### *Generation of stable cell lines*

To generate the stable knockdown cell lines, lentivirus was produced by transfection with shRNA constructs (pLKO.1) and

helper plasmids, psPAX2, and pMD2.G, into HEK 293T cells. Cell-free media containing the virus were filtered using a 0.45

μm syringe filter and transduced with polybrene (8 μg/ml) into A549 cells. Infected cells were cultured and selected with

puromycin (2 μg/ml) for 7 days. The efficiency of knockdown was verified by immunoblotting analysis.

#### *Xenograft study*

Male BALB/c nude mice (6 weeks old) were randomly divided into experimental groups. Mice were subcutaneously injected

with 1 × 107 shControl or shRING1-expressed A549 cells as indicated in the figures in a 100 μl volume using a 27-gauge

needle. After 10 days, the tumor size was monitored every other day. To determine tumor mass, the longest diameter (length and the shortest diameter (width) were measured using a caliper. The tumor mass was calculated by the modified ellipsoida formula: V = (L·W2)/2, where V = volume (in mm3), L = length (in mm), and W = width (in mm).

## Table S1. siRNA, shRNA, and RT-PCR primer sequence

**siRNA**

**Target gene Sequence (5'-3')**

**RING1** siRING1-1 : GAG UGU CCU ACC UGC CGU U

siRING1-2 : CUG CAU UGU CAC AGC CCU ACG GAU U

**CIP2A** siCIP2A-1 : CUG UGG UUG UGU UUG CAC UUU siCIP2A-2 : ACC AUU GAU AUC CUU AGA AUU

**c-MYC** siC-MYC-1 : AAG GAC UAU CCU GCU GCC AAG UU siC-MYC-2 : AAG GUC AGA GUC UGG AUC ACC UU

**PP2Ac** siPP2Ac-1 : CGU GCA AGA GGU UCG AUG UUU siPP2Ac-2 : GGC AGA UCU UCU GUC UAC AUU

**DNMT1** siDNMT1-1 : GGA GAA CGG UGC UCA UGC UUU U siDNMT1-2 : GCC CAA UGA GAC UGA CAU CAA UU

shRING1-1 : CCGGAA GCC CTG ATC TCT AAG ATC TAT * CTCAGA ATA GAT CTT AGA GAT CAG GGC TTTTTG

**shRNA RING1**

**CIP2A RING1**

**Cyclin A**

shRING1-2 : CCGGAA CTG GAG CTG GTG AAT GAG AAA * CTCAGA TTT CTC ATT CAC CAG CTC CAG TTTTTG

Forward : TGC GGC ACT TGG AGG TAA TTT C Reverse : AGC TCT ACA AGG CAA CTC AAG C Forward : CTC CAA GTA CTT GGC CCT GC Reverse : ATC AGG TCC TCC GGT GTC AGA Forward : GAG GTC CCG ATG CTT GTC AG Reverse : GTT AGC AGC CCT AGC ACT GTC

**RT-PCR**

**Primer**

Forward : AAT AAG GCG AAG ATC AAC ATG GC

**Cyclin B**

Reverse : TTT GTT ACC AAT GTC CCC AAG AG

Forward : GCT GCG AAG TGG AAA CCA TC

**Cyclin D Cyclin E β-Actin**

Reverse : CCT CCT TCT GCA CAC ATT TGA A Forward : AAG GAG CGG GAC ACC ATG A Reverse : ACG GTC ACG TTT GCC TTC C Forward : ACT CTT CCA GCC TTC CTT C Reverse : GAT GTC CAC GTC ACA CTT C

## Table S2


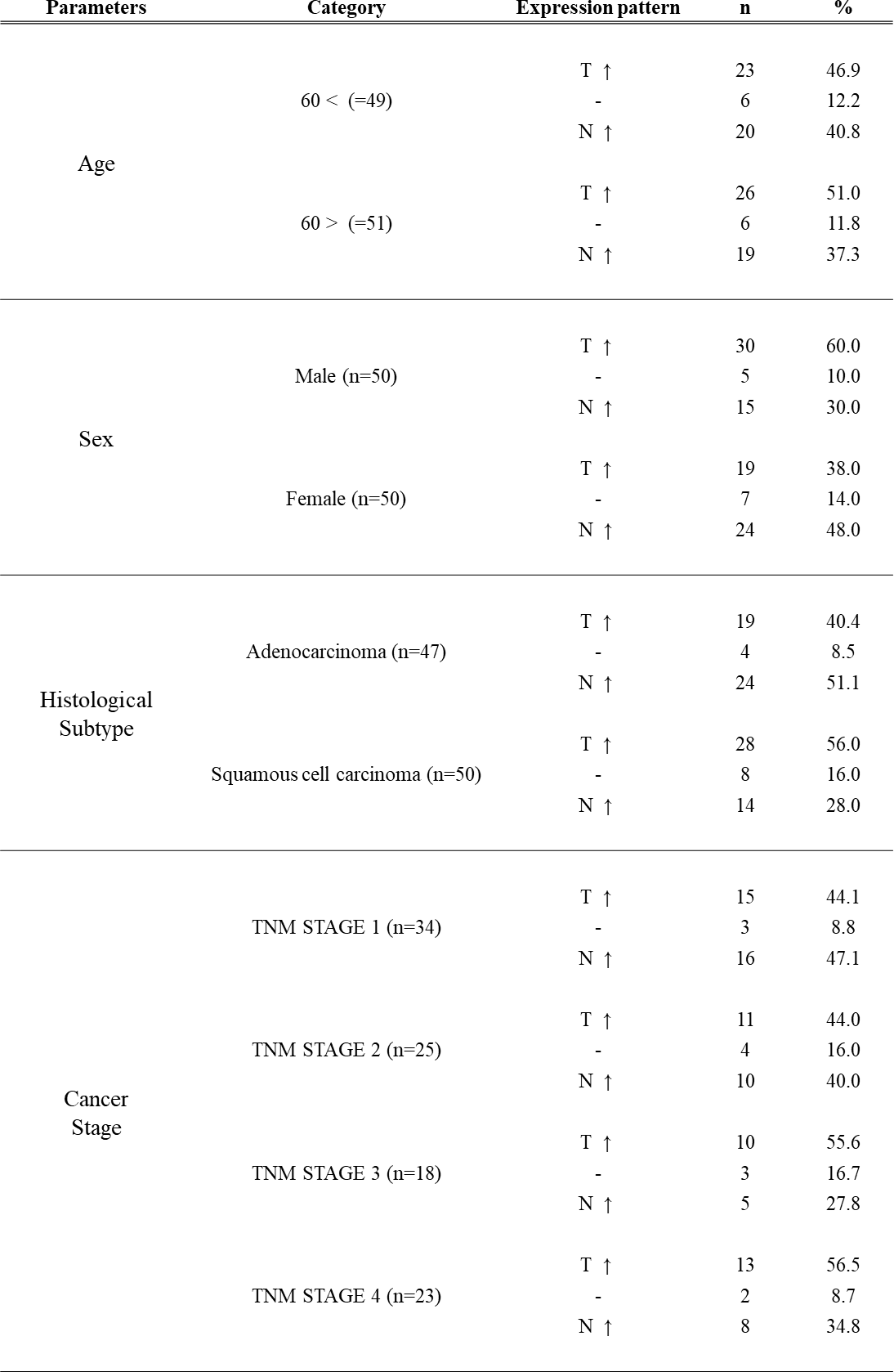


### Supplementary Table 2. CIP2A expression pattern analysis in several parameters.

Analysis of CIP2A protein expression in 100 paired lung cancer tissues, summarized by clinical parameters with

the number of cases and percentages.

# Table S3

**Supplementary Table 3. Mass proteomic analysis_CIP2A**

| **Protein Id** | **Gene Symbol** | **Description** | **peptides** |
| --- | --- | --- | --- |
| **sp\|Q8TCG1\|CIP2A_H** | **KIAA1524** | **CIP2A_HUMAN Protein CIP2A** | **187** |
| sp\|P11142\|HSP7C_HU | HSPA8 | HSP7C_HUMAN Heat shock cognate 71 kDa protein | 67 |
| **sp\|Q92560\|BAP1_HU** | **BAP1** | **BAP1_HUMAN Ubiquitin carboxyl-terminal hydrolase BAP1** | **44** |
| sp\|P16989\|YBOX3_HU | YBX3 | YBOX3_HUMAN Y-box-binding protein 3 | 47 |
| sp\|P09874\|PARP1_HU | PARP1 | PARP1_HUMAN Poly [ADP-ribose] polymerase 1 | 43 |
| sp\|Q4G0J3-3\|LARP7_H | LARP7 | LARP7_HUMAN Isoform 3 of La-related protein 7 | 43 |
| **sp\|Q06587\|RING1_H** | **RING1** | **RING1_HUMAN E3 ubiquitin-protein ligase RING1** | **42** |
| sp\|P38646\|GRP75_HU | HSPA9 | GRP75_HUMAN Stress-70 protein, mitochondrial | 41 |
| sp\|O43390\|HNRPR_HU | HNRNPR | HNRPR_HUMAN Heterogeneous nuclear ribonucleoprotein R | 39 |
| sp\|Q99729-3\|ROAA_H | HNRNPAB | ROAA_HUMAN Isoform 3 of Heterogeneous nuclear ribonucleopro | 37 |
| sp\|O60506\|HNRPQ_H | SYNCRIP | HNRPQ_HUMAN Heterogeneous nuclear ribonucleoprotein Q | 35 |
| sp\|P09012\|SNRPA_HU | SNRPA | SNRPA_HUMAN U1 small nuclear ribonucleoprotein A | 33 |
| sp\|Q6PKG0\|LARP1_HU | LARP1 | LARP1_HUMAN La-related protein 1 | 30 |
| sp\|P09651\|ROA1_HUM | HNRNPA1 | ROA1_HUMAN Heterogeneous nuclear ribonucleoprotein A1 | 29 |
| sp\|P67809\|YBOX1_HU | YBX1 | YBOX1_HUMAN Nuclease-sensitive element-binding protein 1 | 29 |
| sp\|P07910-2\|HNRPC_ | HNRNPC | HNRPC_HUMAN Isoform C1 of Heterogeneous nuclear ribonucleop | 28 |
| sp\|Q00839\|HNRPU_H | HNRNPU | HNRPU_HUMAN Heterogeneous nuclear ribonucleoprotein U | 27 |
| sp\|Q07021\|C1QBP_HU | C1QBP | C1QBP_HUMAN Complement component 1 Q subcomponent-bind | 27 |
| sp\|Q8NC51\|PAIRB_HU | SERBP1 | PAIRB_HUMAN Plasminogen activator inhibitor 1 RNA-binding pro | 27 |
| sp\|P22626\|ROA2_HUM | HNRNPA2B1 | ROA2_HUMAN Heterogeneous nuclear ribonucleoproteins A2/B1 | 25 |
| sp\|P00761\|TRYP_PIG_contaminant | | TRYP_PIG_contaminant Trypsin | 23 |
| sp\|Q08170\|SRSF4_HU | SRSF4 | SRSF4_HUMAN Serine/arginine-rich splicing factor 4 | 23 |
| sp\|P07900-2\|HS90A_H | HSP90AA1 | HS90A_HUMAN Isoform 2 of Heat shock protein HSP 90-alpha | 23 |
| sp\|Q13310-3\|PABP4_H | PABPC4 | PABP4_HUMAN Isoform 3 of Polyadenylate-binding protein 4 | 21 |
| sp\|P11940\|PABP1_HU | PABPC1 | PABP1_HUMAN Polyadenylate-binding protein 1 | 21 |
| sp\|Q69YQ0\|CYTSA_HU | SPECC1L | CYTSA_HUMAN Cytospin-A | 21 |
| sp\|P08670\|VIME_HUM | VIM | VIME_HUMAN Vimentin | 21 |
| sp\|P09661\|RU2A_HUM | SNRPA1 | RU2A_HUMAN U2 small nuclear ribonucleoprotein A' | 21 |
| sp\|Q7KZF4\|SND1_HU | SND1 | SND1_HUMAN Staphylococcal nuclease domain-containing protein | 20 |

| sp\|P61978-2\|HNRPK_ | HNRNPK | HNRPK_HUMAN Isoform 2 of Heterogeneous nuclear ribonucleopr | 19 |
| --- | --- | --- | --- |
| sp\|Q9BRJ6\|CG050_HU | C7orf50 | CG050_HUMAN Uncharacterized protein C7orf50 | 19 |
| sp\|Q07955\|SRSF1_HU | SRSF1 | SRSF1_HUMAN Serine/arginine-rich splicing factor 1 | 19 |
| sp\|Q08211\|DHX9_HU | DHX9 | DHX9_HUMAN ATP-dependent RNA helicase A | 18 |
| sp\|P62258\|1433E_HU | YWHAE | 1433E_HUMAN 14-3-3 protein epsilon | 18 |
| sp\|Q7L2J0\|MEPCE_HU | MEPCE | MEPCE_HUMAN 7SK snRNA methylphosphate capping enzyme | 18 |
| sp\|Q14444\|CAPR1_HU | CAPRIN1 | CAPR1_HUMAN Caprin-1 | 18 |
| sp\|Q9NR30\|DDX21_H | DDX21 | DDX21_HUMAN Nucleolar RNA helicase 2 | 17 |
| sp\|P46783\|RS10_HUM | RPS10 | RS10_HUMAN 40S ribosomal protein S10 | 17 |
| sp\|Q99714\|HCD2_HU | HSD17B10 | HCD2_HUMAN 3-hydroxyacyl-CoA dehydrogenase type-2 | 17 |
| sp\|P63244\|GBLP_HUM | GNB2L1 | GBLP_HUMAN Guanine nucleotide-binding protein subunit beta-2- | 17 |
| sp\|P39023\|RL3_HUMA | RPL3 | RL3_HUMAN 60S ribosomal protein L3 | 16 |
| sp\|P14618\|KPYM_HUM | PKM | KPYM_HUMAN Pyruvate kinase PKM | 16 |
| sp\|Q7L2E3-2\|DHX30_H | DHX30 | DHX30_HUMAN Isoform 2 of Putative ATP-dependent RNA helicas | 16 |
| sp\|P61254\|RL26_HUM | RPL26 | RL26_HUMAN 60S ribosomal protein L26 | 16 |
| sp\|P08621\|RU17_HUM | SNRNP70 | RU17_HUMAN U1 small nuclear ribonucleoprotein 70 kDa | 16 |
| sp\|Q6PCB5\|RSBNL_HU | RSBN1L | RSBNL_HUMAN Round spermatid basic protein 1-like protein | 16 |
| sp\|P62899\|RL31_HUM | RPL31 | RL31_HUMAN 60S ribosomal protein L31 | 15 |
| sp\|P51991\|ROA3_HUM | HNRNPA3 | ROA3_HUMAN Heterogeneous nuclear ribonucleoprotein A3 | 15 |
| sp\|P62736\|ACTA_HUM | ACTA2 | ACTA_HUMAN Actin, aortic smooth muscle | 15 |
| sp\|Q00577\|PURA_HU | PURA | PURA_HUMAN Transcriptional activator protein Pur-alpha | 15 |
| sp\|Q9H6F5\|CCD86_HU | CCDC86 | CCD86_HUMAN Coiled-coil domain-containing protein 86 | 15 |
| sp\|P12532\|KCRU_HUM | CKMT1A | KCRU_HUMAN Creatine kinase U-type, mitochondrial | 15 |
| sp\|Q9Y520-7\|PRC2C_H | PRRC2C | PRC2C_HUMAN Isoform 7 of Protein PRRC2C | 15 |
| sp\|P62316\|SMD2_HU | SNRPD2 | SMD2_HUMAN Small nuclear ribonucleoprotein Sm D2 | 15 |
| sp\|Q13283\|G3BP1_HU | G3BP1 | G3BP1_HUMAN Ras GTPase-activating protein-binding protein 1 | 15 |
| sp\|P17844\|DDX5_HUM | DDX5 | DDX5_HUMAN Probable ATP-dependent RNA helicase DDX5 | 14 |
| sp\|P26373\|RL13_HUM | RPL13 | RL13_HUMAN 60S ribosomal protein L13 | 14 |
| sp\|P62753\|RS6_HUMA | RPS6 | RS6_HUMAN 40S ribosomal protein S6 | 14 |
| sp\|O95793\|STAU1_HU | STAU1 | STAU1_HUMAN Double-stranded RNA-binding protein Staufen ho | 14 |

| sp\|Q13838-2\|DX39B_H | DDX39B | DX39B_HUMAN Isoform 2 of Spliceosome RNA helicase DDX39B | 14 |
| --- | --- | --- | --- |
| sp\|P07237\|PDIA1_HU | P4HB | PDIA1_HUMAN Protein disulfide-isomerase | 14 |
| sp\|P82650\|RT22_HUM | MRPS22 | RT22_HUMAN 28S ribosomal protein S22, mitochondrial | 14 |
| sp\|P62913\|RL11_HUM | RPL11 | RL11_HUMAN 60S ribosomal protein L11 | 14 |
| sp\|P36578\|RL4_HUMA | RPL4 | RL4_HUMAN 60S ribosomal protein L4 | 13 |
| sp\|Q12906-7\|ILF3_HU | ILF3 | ILF3_HUMAN Isoform 7 of Interleukin enhancer-binding factor 3 | 13 |
| sp\|P46781\|RS9_HUMA | RPS9 | RS9_HUMAN 40S ribosomal protein S9 | 13 |
| sp\|O95831\|AIFM1_HU | AIFM1 | AIFM1_HUMAN Apoptosis-inducing factor 1, mitochondrial | 13 |
| sp\|P16402\|H13_HUMA | HIST1H1D | H13_HUMAN Histone H1.3 | 13 |
| sp\|Q14103-4\|HNRPD_ | HNRNPD | HNRPD_HUMAN Isoform 4 of Heterogeneous nuclear ribonucleopr | 13 |
| sp\|P23246\|SFPQ_HUM | SFPQ | SFPQ_HUMAN Splicing factor, proline- and glutamine-rich | 13 |
| sp\|P14625\|ENPL_HUM | HSP90B1 | ENPL_HUMAN Endoplasmin | 13 |
| sp\|Q86V81\|THOC4_HU | ALYREF | THOC4_HUMAN THO complex subunit 4 | 13 |
| sp\|Q02878\|RL6_HUMA | RPL6 | RL6_HUMAN 60S ribosomal protein L6 | 12 |
| sp\|P68363\|TBA1B_HU | TUBA1B | TBA1B_HUMAN Tubulin alpha-1B chain | 12 |
| sp\|Q92841\|DDX17_HU | DDX17 | DDX17_HUMAN Probable ATP-dependent RNA helicase DDX17 | 12 |
| sp\|P08779\|K1C16_HU | KRT16 | K1C16_HUMAN_contaminant Keratin, type I cytoskeletal 16 | 12 |
| sp\|Q5VTE0\|EF1A3_HU | EEF1A1P5 | EF1A3_HUMAN Putative elongation factor 1-alpha-like 3 | 12 |
| sp\|Q13435\|SF3B2_HU | SF3B2 | SF3B2_HUMAN Splicing factor 3B subunit 2 | 12 |
| sp\|P13639\|EF2_HUMA | EEF2 | EF2_HUMAN Elongation factor 2 | 12 |
| sp\|P55072\|TERA_HUM | VCP | TERA_HUMAN Transitional endoplasmic reticulum ATPase | 12 |
| sp\|Q9UKV3\|ACINU_HU | ACIN1 | ACINU_HUMAN Apoptotic chromatin condensation inducer in the | 11 |
| sp\|Q7L0Y3\|MRRP1_HU | TRMT10C | MRRP1_HUMAN Mitochondrial ribonuclease P protein 1 | 11 |
| sp\|Q92522\|H1X_HUM | H1FX | H1X_HUMAN Histone H1x | 11 |
| sp\|P43243\|MATR3_HU | MATR3 | MATR3_HUMAN Matrin-3 | 11 |
| sp\|P42696\|RBM34_HU | RBM34 | RBM34_HUMAN RNA-binding protein 34 | 11 |
| sp\|P14678-3\|RSMB_H | SNRPB | RSMB_HUMAN Isoform SM-B1 of Small nuclear ribonucleoprotein- | 11 |
| sp\|Q9Y3B9\|RRP15_HU | RRP15 | RRP15_HUMAN RRP15-like protein | 11 |
| sp\|Q8NHQ9\|DDX55_H | DDX55 | DDX55_HUMAN ATP-dependent RNA helicase DDX55 | 11 |
| sp\|Q9UN86\|G3BP2_H | G3BP2 | G3BP2_HUMAN Ras GTPase-activating protein-binding protein 2 | 11 |

| sp\|P68371\|TBB4B_HU | TUBB4B | TBB4B_HUMAN Tubulin beta-4B chain | 11 |
| --- | --- | --- | --- |
| sp\|P23588\|IF4B_HUMA | EIF4B | IF4B_HUMAN Eukaryotic translation initiation factor 4B | 11 |
| sp\|Q53F19\|CQ085_HU | C17orf85 | CQ085_HUMAN Uncharacterized protein C17orf85 | 11 |
| sp\|O14979\|HNRDL_HU | HNRNPDL | HNRDL_HUMAN Heterogeneous nuclear ribonucleoprotein D-like | 10 |
| sp\|Q9UBS4\|DJB11_HU | DNAJB11 | DJB11_HUMAN DnaJ homolog subfamily B member 11 | 10 |
| sp\|P02533\|K1C14_HU | KRT14 | K1C14_HUMAN_contaminant Keratin, type I cytoskeletal 14 | 10 |
| sp\|Q9UQ80\|PA2G4_H | PA2G4 | PA2G4_HUMAN Proliferation-associated protein 2G4 | 10 |
| sp\|Q92734\|TFG_HUM | TFG | TFG_HUMAN Protein TFG | 10 |
| sp\|P0CW22\|RS17L_HU | RPS17L | RS17L_HUMAN 40S ribosomal protein S17-like | 10 |
| sp\|P23528\|COF1_HUM | CFL1 | COF1_HUMAN Cofilin-1 | 10 |
| sp\|Q92552\|RT27_HUM | MRPS27 | RT27_HUMAN 28S ribosomal protein S27, mitochondrial | 10 |
| sp\|P62937\|PPIA_HUM | PPIA | PPIA_HUMAN Peptidyl-prolyl cis-trans isomerase A | 10 |
| sp\|Q04837\|SSBP_HUM | SSBP1 | SSBP_HUMAN Single-stranded DNA-binding protein, mitochondrial | 10 |
| sp\|Q13601\|KRR1_HUM | KRR1 | KRR1_HUMAN KRR1 small subunit processome component homolo | 10 |
| sp\|Q15233\|NONO_HU | NONO | NONO_HUMAN Non-POU domain-containing octamer-binding pro | 10 |
| sp\|Q15084-2\|PDIA6_H | PDIA6 | PDIA6_HUMAN Isoform 2 of Protein disulfide-isomerase A6 | 10 |
| sp\|Q9BU76\|MMTA2_H | MMTAG2 | MMTA2_HUMAN Multiple myeloma tumor-associated protein 2 | 10 |
| sp\|O43290\|SNUT1_HU | SART1 | SNUT1_HUMAN U4/U6.U5 tri-snRNP-associated protein 1 | 10 |
| sp\|Q9NTK5\|OLA1_HU | OLA1 | OLA1_HUMAN Obg-like ATPase 1 | 10 |
| sp\|P46779\|RL28_HUM | RPL28 | RL28_HUMAN 60S ribosomal protein L28 | 9 |
| sp\|P62917\|RL8_HUMA | RPL8 | RL8_HUMAN 60S ribosomal protein L8 | 9 |
| sp\|P0CG48\|UBC_HUM | UBC | UBC_HUMAN Polyubiquitin-C | 11 |
| sp\|P30050\|RL12_HUM | RPL12 | RL12_HUMAN 60S ribosomal protein L12 | 9 |
| sp\|P63241-2\|IF5A1_HU | EIF5A | IF5A1_HUMAN Isoform 2 of Eukaryotic translation initiation factor | 9 |
| sp\|P24534\|EF1B_HUM | EEF1B2 | EF1B_HUMAN Elongation factor 1-beta | 9 |
| sp\|Q8N9T8\|KRI1_HUM | KRI1 | KRI1_HUMAN Protein KRI1 homolog | 9 |
| sp\|P62841\|RS15_HUM | RPS15 | RS15_HUMAN 40S ribosomal protein S15 | 9 |
| sp\|P62851\|RS25_HUM | RPS25 | RS25_HUMAN 40S ribosomal protein S25 | 9 |
| sp\|P38159\|RBMX_HU | RBMX | RBMX_HUMAN RNA-binding motif protein, X chromosome | 9 |
| sp\|P12956\|XRCC6_HU | XRCC6 | XRCC6_HUMAN X-ray repair cross-complementing protein 6 | 9 |

| sp\|Q5QNW6-2\|H2B2F | HIST2H2BF | H2B2F_HUMAN Isoform 2 of Histone H2B type 2-F | 9 |
| --- | --- | --- | --- |
| sp\|Q14697-2\|GANAB_ | GANAB | GANAB_HUMAN Isoform 2 of Neutral alpha-glucosidase AB | 9 |
| sp\|O75534-3\|CSDE1_H | CSDE1 | CSDE1_HUMAN Isoform 3 of Cold shock domain-containing protei | 9 |
| sp\|Q9UKM9\|RALY_HU | RALY | RALY_HUMAN RNA-binding protein Raly | 9 |
| sp\|P62244\|RS15A_HU | RPS15A | RS15A_HUMAN 40S ribosomal protein S15a | 9 |
| sp\|O60832\|DKC1_HUM | DKC1 | DKC1_HUMAN H/ACA ribonucleoprotein complex subunit 4 | 9 |
| sp\|P55209\|NP1L1_HU | NAP1L1 | NP1L1_HUMAN Nucleosome assembly protein 1-like 1 | 9 |
| sp\|Q14011\|CIRBP_HU | CIRBP | CIRBP_HUMAN Cold-inducible RNA-binding protein | 9 |
| sp\|Q14498\|RBM39_HU | RBM39 | RBM39_HUMAN RNA-binding protein 39 | 9 |
| sp\|Q92900\|RENT1_HU | UPF1 | RENT1_HUMAN Regulator of nonsense transcripts 1 | 9 |
| sp\|P62263\|RS14_HUM | RPS14 | RS14_HUMAN 40S ribosomal protein S14 | 9 |
| sp\|P62847-4\|RS24_HU | RPS24 | RS24_HUMAN Isoform 4 of 40S ribosomal protein S24 | 8 |
| sp\|P46778\|RL21_HUM | RPL21 | RL21_HUMAN 60S ribosomal protein L21 | 8 |
| sp\|P32969\|RL9_HUMA | RPL9 | RL9_HUMAN 60S ribosomal protein L9 | 8 |
| sp\|P62277\|RS13_HUM | RPS13 | RS13_HUMAN 40S ribosomal protein S13 | 8 |
| sp\|P62269\|RS18_HUM | RPS18 | RS18_HUMAN 40S ribosomal protein S18 | 8 |
| sp\|P61313\|RL15_HUM | RPL15 | RL15_HUMAN 60S ribosomal protein L15 | 8 |
| sp\|P18621-3\|RL17_HU | RPL17 | RL17_HUMAN Isoform 3 of 60S ribosomal protein L17 | 8 |
| sp\|P62424\|RL7A_HUM | RPL7A | RL7A_HUMAN 60S ribosomal protein L7a | 8 |
| sp\|P62280\|RS11_HUM | RPS11 | RS11_HUMAN 40S ribosomal protein S11 | 8 |
| sp\|P08238\|HS90B_HU | HSP90AB1 | HS90B_HUMAN Heat shock protein HSP 90-beta | 8 |
| sp\|P04406\|G3P_HUMA | GAPDH | G3P_HUMAN Glyceraldehyde-3-phosphate dehydrogenase | 8 |
| sp\|P35579\|MYH9_HU | MYH9 | MYH9_HUMAN Myosin-9 | 8 |
| sp\|Q9BUJ2\|HNRL1_HU | HNRNPUL1 | HNRL1_HUMAN Heterogeneous nuclear ribonucleoprotein U-like p | 8 |
| sp\|Q06787-7\|FMR1_H | FMR1 | FMR1_HUMAN Isoform 7 of Fragile X mental retardation protein 1 | 8 |
| sp\|Q86UE4\|LYRIC_HU | MTDH | LYRIC_HUMAN Protein LYRIC | 8 |
| sp\|Q13185\|CBX3_HUM | CBX3 | CBX3_HUMAN Chromobox protein homolog 3 | 8 |
| sp\|P48634\|PRC2A_HU | PRRC2A | PRC2A_HUMAN Protein PRRC2A | 8 |
| sp\|Q13895\|BYST_HUM | BYSL | BYST_HUMAN Bystin | 8 |
| sp\|Q15366-2\|PCBP2_H | PCBP2 | PCBP2_HUMAN Isoform 2 of Poly(rC)-binding protein 2 | 8 |

| sp\|Q2NL82\|TSR1_HUM | TSR1 | TSR1_HUMAN Pre-rRNA-processing protein TSR1 homolog | 8 |
| --- | --- | --- | --- |
| sp\|P08579\|RU2B_HUM | SNRPB2 | RU2B_HUMAN U2 small nuclear ribonucleoprotein B'' | 8 |
| sp\|P62829\|RL23_HUM | RPL23 | RL23_HUMAN 60S ribosomal protein L23 | 7 |
| sp\|P83731\|RL24_HUM | RPL24 | RL24_HUMAN 60S ribosomal protein L24 | 7 |
| sp\|P04259\|K2C6B_HU | KRT6B | K2C6B_HUMAN_contaminant Keratin, type II cytoskeletal 6B | 7 |
| sp\|P07737\|PROF1_HU | PFN1 | PROF1_HUMAN Profilin-1 | 7 |
| sp\|P40429\|RL13A_HU | RPL13A | RL13A_HUMAN 60S ribosomal protein L13a | 7 |
| sp\|Q9UKD2\|MRT4_HU | MRTO4 | MRT4_HUMAN mRNA turnover protein 4 homolog | 7 |
| sp\|Q9BZI7\|REN3B_HU | UPF3B | REN3B_HUMAN Regulator of nonsense transcripts 3B | 7 |
| sp\|Q5VWQ0\|RSBN1_H | RSBN1 | RSBN1_HUMAN Round spermatid basic protein 1 | 7 |
| sp\|P35637\|FUS_HUMA | FUS | FUS_HUMAN RNA-binding protein FUS | 7 |
| sp\|A8MWD9\|RUXGL_HUMAN | | RUXGL_HUMAN Small nuclear ribonucleoprotein G-like protein | 7 |
| sp\|Q99575\|POP1_HUM | POP1 | POP1_HUMAN Ribonucleases P/MRP protein subunit POP1 | 7 |
| sp\|Q13243\|SRSF5_HU | SRSF5 | SRSF5_HUMAN Serine/arginine-rich splicing factor 5 | 7 |
| sp\|P51114\|FXR1_HUM | FXR1 | FXR1_HUMAN Fragile X mental retardation syndrome-related prote | 7 |
| sp\|Q8WXX5\|DNJC9_H | DNAJC9 | DNJC9_HUMAN DnaJ homolog subfamily C member 9 | 7 |
| sp\|Q00059\|TFAM_HU | TFAM | TFAM_HUMAN Transcription factor A, mitochondrial | 7 |
| sp\|Q13242\|SRSF9_HU | SRSF9 | SRSF9_HUMAN Serine/arginine-rich splicing factor 9 | 7 |
| sp\|Q15046\|SYK_HUMA | KARS | SYK_HUMAN Lysine--tRNA ligase | 7 |
| sp\|Q9Y224\|CN166_HU | C14orf166 | CN166_HUMAN UPF0568 protein C14orf166 | 7 |
| sp\|Q13247\|SRSF6_HU | SRSF6 | SRSF6_HUMAN Serine/arginine-rich splicing factor 6 | 7 |
| sp\|P83916\|CBX1_HUM | CBX1 | CBX1_HUMAN Chromobox protein homolog 1 | 7 |
| sp\|Q13263\|TIF1B_HUM | TRIM28 | TIF1B_HUMAN Transcription intermediary factor 1-beta | 7 |
| sp\|O15446-2\|RPA34_H | CD3EAP | RPA34_HUMAN Isoform 2 of DNA-directed RNA polymerase I sub | 7 |
| sp\|Q9H814\|PHAX_HU | PHAX | PHAX_HUMAN Phosphorylated adapter RNA export protein | 7 |
| sp\|Q9UN81\|LORF1_HU | L1RE1 | LORF1_HUMAN LINE-1 retrotransposable element ORF1 protein | 7 |
| sp\|P31943\|HNRH1_HU | HNRNPH1 | HNRH1_HUMAN Heterogeneous nuclear ribonucleoprotein H | 7 |
| sp\|P53999\|TCP4_HUM | SUB1 | TCP4_HUMAN Activated RNA polymerase II transcriptional coactiva | 7 |
| sp\|P78371\|TCPB_HUM | CCT2 | TCPB_HUMAN T-complex protein 1 subunit beta | 7 |
| sp\|P46782\|RS5_HUMA | RPS5 | RS5_HUMAN 40S ribosomal protein S5 | 7 |

| sp\|Q9H2W6\|RM46_HU | MRPL46 | RM46_HUMAN 39S ribosomal protein L46, mitochondrial | 7 |
| --- | --- | --- | --- |
| sp\|O75683\|SURF6_HU | SURF6 | SURF6_HUMAN Surfeit locus protein 6 | 7 |
| sp\|Q9BTD8\|RBM42_H | RBM42 | RBM42_HUMAN RNA-binding protein 42 | 7 |
| sp\|Q8WXF1\|PSPC1_HU | PSPC1 | PSPC1_HUMAN Paraspeckle component 1 | 7 |
| sp\|P52815\|RM12_HUM | MRPL12 | RM12_HUMAN 39S ribosomal protein L12, mitochondrial | 7 |
| sp\|P06576\|ATPB_HUM | ATP5B | ATPB_HUMAN ATP synthase subunit beta, mitochondrial | 7 |
| sp\|P98175\|RBM10_HU | RBM10 | RBM10_HUMAN RNA-binding protein 10 | 7 |
| sp\|Q15365\|PCBP1_HU | PCBP1 | PCBP1_HUMAN Poly(rC)-binding protein 1 | 7 |
| sp\|Q96CT7\|CC124_HU | CCDC124 | CC124_HUMAN Coiled-coil domain-containing protein 124 | 7 |
| sp\|P12277\|KCRB_HUM | CKB | KCRB_HUMAN Creatine kinase B-type | 7 |
| sp\|P84103\|SRSF3_HU | SRSF3 | SRSF3_HUMAN Serine/arginine-rich splicing factor 3 | 7 |
| sp\|Q02543\|RL18A_HU | RPL18A | RL18A_HUMAN 60S ribosomal protein L18a | 6 |
| sp\|Q9BQG0-2\|MBB1A | MYBBP1A | MBB1A_HUMAN Isoform 2 of Myb-binding protein 1A | 6 |
| sp\|P37108\|SRP14_HU | SRP14 | SRP14_HUMAN Signal recognition particle 14 kDa protein | 6 |
| sp\|Q9NZI8\|IF2B1_HUM | IGF2BP1 | IF2B1_HUMAN Insulin-like growth factor 2 mRNA-binding protein | 6 |
| sp\|O43143\|DHX15_HU | DHX15 | DHX15_HUMAN Putative pre-mRNA-splicing factor ATP-dependent | 6 |
| sp\|P17987\|TCPA_HUM | TCP1 | TCPA_HUMAN T-complex protein 1 subunit alpha | 6 |
| sp\|P22087\|FBRL_HUM | FBL | FBRL_HUMAN rRNA 2'-O-methyltransferase fibrillarin | 6 |
| sp\|P10412\|H14_HUMA | HIST1H1E | H14_HUMAN Histone H1.4 | 6 |
| sp\|Q99848\|EBP2_HUM | EBNA1BP2 | EBP2_HUMAN Probable rRNA-processing protein EBP2 | 6 |
| sp\|Q14684\|RRP1B_HU | RRP1B | RRP1B_HUMAN Ribosomal RNA processing protein 1 homolog B | 6 |
| sp\|P35580-4\|MYH10_ | MYH10 | MYH10_HUMAN Isoform 4 of Myosin-10 | 6 |
| sp\|P38919\|IF4A3_HUM | EIF4A3 | IF4A3_HUMAN Eukaryotic initiation factor 4A-III | 6 |
| sp\|P47914\|RL29_HUM | RPL29 | RL29_HUMAN 60S ribosomal protein L29 | 6 |
| sp\|Q02809\|PLOD1_HU | PLOD1 | PLOD1_HUMAN Procollagen-lysine,2-oxoglutarate 5-dioxygenase 1 | 6 |
| sp\|Q9P015\|RM15_HU | MRPL15 | RM15_HUMAN 39S ribosomal protein L15, mitochondrial | 6 |
| sp\|Q9NYF8\|BCLF1_HU | BCLAF1 | BCLF1_HUMAN Bcl-2-associated transcription factor 1 | 6 |
| sp\|Q9BVP2\|GNL3_HU | GNL3 | GNL3_HUMAN Guanine nucleotide-binding protein-like 3 | 6 |
| sp\|Q07666\|KHDR1_HU | KHDRBS1 | KHDR1_HUMAN KH domain-containing, RNA-binding, signal transd | 6 |
| sp\|Q09028\|RBBP4_HU | RBBP4 | RBBP4_HUMAN Histone-binding protein RBBP4 | 6 |

| sp\|P50991\|TCPD_HUM | CCT4 | TCPD_HUMAN T-complex protein 1 subunit delta | 6 |
| --- | --- | --- | --- |
| sp\|P26368\|U2AF2_HU | U2AF2 | U2AF2_HUMAN Splicing factor U2AF 65 kDa subunit | 6 |
| sp\|Q969S3\|ZN622_HU | ZNF622 | ZN622_HUMAN Zinc finger protein 622 | 6 |
| sp\|P62861\|RS30_HUM | FAU | RS30_HUMAN 40S ribosomal protein S30 | 6 |
| sp\|Q15717\|ELAV1_HU | ELAVL1 | ELAV1_HUMAN ELAV-like protein 1 | 6 |
| sp\|P43487\|RANG_HU | RANBP1 | RANG_HUMAN Ran-specific GTPase-activating protein | 6 |
| sp\|P09211\|GSTP1_HU | GSTP1 | GSTP1_HUMAN Glutathione S-transferase P | 6 |
| sp\|O96019\|ACL6A_HU | ACTL6A | ACL6A_HUMAN Actin-like protein 6A | 6 |
| sp\|Q5T3I0-3\|GPTC4_H | GPATCH4 | GPTC4_HUMAN Isoform 3 of G patch domain-containing protein 4 | 6 |
| sp\|Q9NQ55-3\|SSF1_H | PPAN | SSF1_HUMAN Isoform 3 of Suppressor of SWI4 1 homolog | 6 |
| sp\|Q9BYN8\|RT26_HU | MRPS26 | RT26_HUMAN 28S ribosomal protein S26, mitochondrial | 6 |
| sp\|O75400\|PR40A_HU | PRPF40A | PR40A_HUMAN Pre-mRNA-processing factor 40 homolog A | 6 |
| sp\|P23284\|PPIB_HUM | PPIB | PPIB_HUMAN Peptidyl-prolyl cis-trans isomerase B | 6 |
| sp\|P04075-2\|ALDOA_ | ALDOA | ALDOA_HUMAN Isoform 2 of Fructose-bisphosphate aldolase A | 6 |
| sp\|Q9BRP8\|WIBG_HU | WIBG | WIBG_HUMAN Partner of Y14 and mago | 6 |
| sp\|O75494\|SRS10_HU | SRSF10 | SRS10_HUMAN Serine/arginine-rich splicing factor 10 | 6 |
| sp\|P09234\|RU1C_HUM | SNRPC | RU1C_HUMAN U1 small nuclear ribonucleoprotein C | 6 |
| sp\|Q00688\|FKBP3_HU | FKBP3 | FKBP3_HUMAN Peptidyl-prolyl cis-trans isomerase FKBP3 | 6 |
| sp\|Q13765-2\|NACA_H | NACA | NACA_HUMAN Isoform 2 of Nascent polypeptide-associated comp | 6 |
| sp\|Q5BKZ1\|ZN326_HU | ZNF326 | ZN326_HUMAN DBIRD complex subunit ZNF326 | 6 |
| sp\|P60660-2\|MYL6_HU | MYL6 | MYL6_HUMAN Isoform Smooth muscle of Myosin light polypeptid | 6 |
| sp\|Q8N5L8\|RP25L_HU | RPP25L | RP25L_HUMAN Ribonuclease P protein subunit p25-like protein | 6 |
| sp\|O15347\|HMGB3_H | HMGB3 | HMGB3_HUMAN High mobility group protein B3 | 6 |
| sp\|P06733\|ENOA_HU | ENO1 | ENOA_HUMAN Alpha-enolase | 6 |
| sp\|Q07020\|RL18_HUM | RPL18 | RL18_HUMAN 60S ribosomal protein L18 | 5 |
| sp\|P14866\|HNRPL_HU | HNRNPL | HNRPL_HUMAN Heterogeneous nuclear ribonucleoprotein L | 5 |
| sp\|Q9BZE4\|NOG1_HU | GTPBP4 | NOG1_HUMAN Nucleolar GTP-binding protein 1 | 5 |
| sp\|P62249\|RS16_HUM | RPS16 | RS16_HUMAN 40S ribosomal protein S16 | 5 |
| sp\|Q04695\|K1C17_HU | KRT17 | K1C17_HUMAN_contaminant Keratin, type I cytoskeletal 17 | 5 |
| sp\|Q01844-5\|EWS_HU | EWSR1 | EWS_HUMAN Isoform 5 of RNA-binding protein EWS | 5 |

| sp\|Q9Y3D9\|RT23_HUM | MRPS23 | RT23_HUMAN 28S ribosomal protein S23, mitochondrial | 5 |
| --- | --- | --- | --- |
| tr\|F8W7C6\|F8W7C6_H | RPL10 | F8W7C6_HUMAN 60S ribosomal protein L10 | 5 |
| sp\|P49207\|RL34_HUM | RPL34 | RL34_HUMAN 60S ribosomal protein L34 | 5 |
| sp\|P62241\|RS8_HUMA | RPS8 | RS8_HUMAN 40S ribosomal protein S8 | 5 |
| sp\|Q96GA3\|LTV1_HUM | LTV1 | LTV1_HUMAN Protein LTV1 homolog | 5 |
| sp\|P61927\|RL37_HUM | RPL37 | RL37_HUMAN 60S ribosomal protein L37 | 5 |
| sp\|P16104\|H2AX_HUM | H2AFX | H2AX_HUMAN Histone H2AX | 5 |
| sp\|Q15029\|U5S1_HUM | EFTUD2 | U5S1_HUMAN 116 kDa U5 small nuclear ribonucleoprotein compo | 5 |
| sp\|Q9Y265\|RUVB1_HU | RUVBL1 | RUVB1_HUMAN RuvB-like 1 | 5 |
| sp\|P15924\|DESP_HUM | DSP | DESP_HUMAN_contaminant Desmoplakin | 5 |
| sp\|P49411\|EFTU_HUM | TUFM | EFTU_HUMAN Elongation factor Tu, mitochondrial | 5 |
| sp\|Q8TDN6\|BRX1_HU | BRIX1 | BRX1_HUMAN Ribosome biogenesis protein BRX1 homolog | 5 |
| sp\|Q9UMS4\|PRP19_H | PRPF19 | PRP19_HUMAN Pre-mRNA-processing factor 19 | 5 |
| sp\|Q15014\|MO4L2_HU | MORF4L2 | MO4L2_HUMAN Mortality factor 4-like protein 2 | 5 |
| sp\|P62995\|TRA2B_HU | TRA2B | TRA2B_HUMAN Transformer-2 protein homolog beta | 5 |
| sp\|Q99497\|PARK7_HU | PARK7 | PARK7_HUMAN Protein DJ-1 | 5 |
| sp\|P31942\|HNRH3_HU | HNRNPH3 | HNRH3_HUMAN Heterogeneous nuclear ribonucleoprotein H3 | 5 |
| sp\|Q13823\|NOG2_HU | GNL2 | NOG2_HUMAN Nucleolar GTP-binding protein 2 | 5 |
| sp\|P32119\|PRDX2_HU | PRDX2 | PRDX2_HUMAN Peroxiredoxin-2 | 5 |
| sp\|Q9Y3U8\|RL36_HUM | RPL36 | RL36_HUMAN 60S ribosomal protein L36 | 5 |
| sp\|Q12873-3\|CHD3_H | CHD3 | CHD3_HUMAN Isoform 3 of Chromodomain-helicase-DNA-binding | 5 |
| sp\|P23526\|SAHH_HUM | AHCY | SAHH_HUMAN Adenosylhomocysteinase | 5 |
| sp\|Q14694-2\|UBP10_H | USP10 | UBP10_HUMAN Isoform 2 of Ubiquitin carboxyl-terminal hydrolase | 4 |
| sp\|P07195\|LDHB_HUM | LDHB | LDHB_HUMAN L-lactate dehydrogenase B chain | 5 |
| sp\|Q15691\|MARE1_HU | MAPRE1 | MARE1_HUMAN Microtubule-associated protein RP/EB family mem | 5 |
| sp\|O15234\|CASC3_HU | CASC3 | CASC3_HUMAN Protein CASC3 | 5 |
| sp\|P13010\|XRCC5_HU | XRCC5 | XRCC5_HUMAN X-ray repair cross-complementing protein 5 | 5 |
| sp\|Q6UN15\|FIP1_HUM | FIP1L1 | FIP1_HUMAN Pre-mRNA 3'-end-processing factor FIP1 | 5 |
| sp\|P02538\|K2C6A_HU | KRT6A | K2C6A_HUMAN_contaminant Keratin, type II cytoskeletal 6A | 5 |
| sp\|P13667\|PDIA4_HU | PDIA4 | PDIA4_HUMAN Protein disulfide-isomerase A4 | 5 |

| sp\|P09496-2\|CLCA_HU | CLTA | CLCA_HUMAN Isoform Non-brain of Clathrin light chain A | 5 |
| --- | --- | --- | --- |
| sp\|Q92843-2\|B2CL2_H | BCL2L2 | B2CL2_HUMAN Isoform 3 of Bcl-2-like protein 2 | 5 |
| sp\|O75940\|SPF30_HU | SMNDC1 | SPF30_HUMAN Survival of motor neuron-related-splicing factor 30 | 5 |
| sp\|P62304\|RUXE_HUM | SNRPE | RUXE_HUMAN Small nuclear ribonucleoprotein E | 5 |
| sp\|O60869\|EDF1_HUM | EDF1 | EDF1_HUMAN Endothelial differentiation-related factor 1 | 5 |
| sp\|P61604\|CH10_HUM | HSPE1 | CH10_HUMAN 10 kDa heat shock protein, mitochondrial | 5 |
| sp\|P26599-3\|PTBP1_H | PTBP1 | PTBP1_HUMAN Isoform 3 of Polypyrimidine tract-binding protein 1 | 5 |
| sp\|Q15056\|IF4H_HUM | EIF4H | IF4H_HUMAN Eukaryotic translation initiation factor 4H | 5 |
| sp\|P22392-2\|NDKB_H | NME2 | NDKB_HUMAN Isoform 3 of Nucleoside diphosphate kinase B | 5 |
| sp\|Q13151\|ROA0_HU | HNRNPA0 | ROA0_HUMAN Heterogeneous nuclear ribonucleoprotein A0 | 5 |
| sp\|P18583-9\|SON_HU | SON | SON_HUMAN Isoform I of Protein SON | 5 |
| sp\|P62805\|H4_HUMA | HIST1H4A | H4_HUMAN Histone H4 | 4 |
| sp\|P18077\|RL35A_HU | RPL35A | RL35A_HUMAN 60S ribosomal protein L35a | 4 |
| sp\|P62854\|RS26_HUM | RPS26 | RS26_HUMAN 40S ribosomal protein S26 | 4 |
| sp\|Q92499\|DDX1_HU | DDX1 | DDX1_HUMAN ATP-dependent RNA helicase DDX1 | 4 |
| sp\|O43395\|PRPF3_HU | PRPF3 | PRPF3_HUMAN U4/U6 small nuclear ribonucleoprotein Prp3 | 4 |
| sp\|P60842\|IF4A1_HUM | EIF4A1 | IF4A1_HUMAN Eukaryotic initiation factor 4A-I | 4 |
| sp\|P16403\|H12_HUMA | HIST1H1C | H12_HUMAN Histone H1.2 | 4 |
| sp\|O43818\|U3IP2_HU | RRP9 | U3IP2_HUMAN U3 small nucleolar RNA-interacting protein 2 | 4 |
| sp\|Q96GC5\|RM48_HU | MRPL48 | RM48_HUMAN 39S ribosomal protein L48, mitochondrial | 4 |
| sp\|Q9Y6M1\|IF2B2_HU | IGF2BP2 | IF2B2_HUMAN Insulin-like growth factor 2 mRNA-binding protein | 4 |
| sp\|Q9H5H4\|ZN768_H | ZNF768 | ZN768_HUMAN Zinc finger protein 768 | 4 |
| sp\|P10155\|RO60_HUM | TROVE2 | RO60_HUMAN 60 kDa SS-A/Ro ribonucleoprotein | 4 |
| sp\|Q9HCE1\|MOV10_H | MOV10 | MOV10_HUMAN Putative helicase MOV-10 | 4 |
| sp\|P78345\|RPP38_HU | RPP38 | RPP38_HUMAN Ribonuclease P protein subunit p38 | 4 |
| sp\|Q9GZT3\|SLIRP_HU | SLIRP | SLIRP_HUMAN SRA stem-loop-interacting RNA-binding protein, mi | 4 |
| sp\|P26641\|EF1G_HUM | EEF1G | EF1G_HUMAN Elongation factor 1-gamma | 4 |
| sp\|Q96EY4\|TMA16_HU | TMA16 | TMA16_HUMAN Translation machinery-associated protein 16 | 4 |
| sp\|Q9Y383\|LC7L2_HU | LUC7L2 | LC7L2_HUMAN Putative RNA-binding protein Luc7-like 2 | 4 |
| sp\|Q16531\|DDB1_HU | DDB1 | DDB1_HUMAN DNA damage-binding protein 1 | 4 |

| sp\|Q5SSJ5\|HP1B3_HU | HP1BP3 | HP1B3_HUMAN Heterochromatin protein 1-binding protein 3 | 4 |
| --- | --- | --- | --- |
| sp\|Q4VC05\|BCL7A_HU | BCL7A | BCL7A_HUMAN B-cell CLL/lymphoma 7 protein family member A | 4 |
| sp\|Q01130\|SRSF2_HU | SRSF2 | SRSF2_HUMAN Serine/arginine-rich splicing factor 2 | 4 |
| sp\|Q9Y230\|RUVB2_HU | RUVBL2 | RUVB2_HUMAN RuvB-like 2 | 4 |
| sp\|Q15424-3\|SAFB1_H | SAFB | SAFB1_HUMAN Isoform 3 of Scaffold attachment factor B1 | 4 |
| sp\|Q12849\|GRSF1_HU | GRSF1 | GRSF1_HUMAN G-rich sequence factor 1 | 4 |
| sp\|P60709\|ACTB_HUM | ACTB | ACTB_HUMAN Actin, cytoplasmic 1 | 4 |
| sp\|P62314\|SMD1_HU | SNRPD1 | SMD1_HUMAN Small nuclear ribonucleoprotein Sm D1 | 4 |
| sp\|P27797\|CALR_HUM | CALR | CALR_HUMAN Calreticulin | 4 |
| sp\|Q9H078-2\|CLPB_H | CLPB | CLPB_HUMAN Isoform 2 of Caseinolytic peptidase B protein homo | 4 |
| sp\|P23368\|MAOM_HU | ME2 | MAOM_HUMAN NAD-dependent malic enzyme, mitochondrial | 4 |
| sp\|P09497-2\|CLCB_HU | CLTB | CLCB_HUMAN Isoform Non-brain of Clathrin light chain B | 4 |
| sp\|Q9BS26\|ERP44_HU | ERP44 | ERP44_HUMAN Endoplasmic reticulum resident protein 44 | 4 |
| sp\|P31948\|STIP1_HUM | STIP1 | STIP1_HUMAN Stress-induced-phosphoprotein 1 | 4 |
| sp\|P30405\|PPIF_HUMA | PPIF | PPIF_HUMAN Peptidyl-prolyl cis-trans isomerase F, mitochondrial | 4 |
| sp\|P31946\|1433B_HU | YWHAB | 1433B_HUMAN 14-3-3 protein beta/alpha | 4 |
| sp\|P54886\|P5CS_HUM | ALDH18A1 | P5CS_HUMAN Delta-1-pyrroline-5-carboxylate synthase | 4 |
| sp\|P20290\|BTF3_HUM | BTF3 | BTF3_HUMAN Transcription factor BTF3 | 4 |
| sp\|P35241-5\|RADI_HU | RDX | RADI_HUMAN Isoform 5 of Radixin | 4 |
| sp\|O75937\|DNJC8_HU | DNAJC8 | DNJC8_HUMAN DnaJ homolog subfamily C member 8 | 4 |
| sp\|P60174\|TPIS_HUMA | TPI1 | TPIS_HUMAN Triosephosphate isomerase | 4 |
| sp\|P40926\|MDHM_HU | MDH2 | MDHM_HUMAN Malate dehydrogenase, mitochondrial | 4 |
| sp\|P16989-2\|YBOX3_H | YBX3 | YBOX3_HUMAN Isoform 2 of Y-box-binding protein 3 | 4 |
| sp\|P62266\|RS23_HUM | RPS23 | RS23_HUMAN 40S ribosomal protein S23 | 3 |
| sp\|P31689\|DNJA1_HU | DNAJA1 | DNJA1_HUMAN DnaJ homolog subfamily A member 1 | 3 |
| sp\|P83881\|RL36A_HU | RPL36A | RL36A_HUMAN 60S ribosomal protein L36a | 3 |
| sp\|P62910\|RL32_HUM | RPL32 | RL32_HUMAN 60S ribosomal protein L32 | 3 |
| sp\|Q9H0S4\|DDX47_HU | DDX47 | DDX47_HUMAN Probable ATP-dependent RNA helicase DDX47 | 3 |
| sp\|P62888\|RL30_HUM | RPL30 | RL30_HUMAN 60S ribosomal protein L30 | 3 |
| sp\|Q9NX58\|LYAR_HU | LYAR | LYAR_HUMAN Cell growth-regulating nucleolar protein | 3 |

| sp\|Q9NP64\|NO40_HU | ZCCHC17 | NO40_HUMAN Nucleolar protein of 40 kDa | 3 |
| --- | --- | --- | --- |
| sp\|P68431\|H31_HUMA | HIST1H3A | H31_HUMAN Histone H3.1 | 3 |
| sp\|Q8IYB3\|SRRM1_HU | SRRM1 | SRRM1_HUMAN Serine/arginine repetitive matrix protein 1 | 3 |
| sp\|O14818\|PSA7_HUM | PSMA7 | PSA7_HUMAN Proteasome subunit alpha type-7 | 3 |
| sp\|P62318\|SMD3_HU | SNRPD3 | SMD3_HUMAN Small nuclear ribonucleoprotein Sm D3 | 3 |
| sp\|P60866\|RS20_HUM | RPS20 | RS20_HUMAN 40S ribosomal protein S20 | 3 |
| sp\|P14923\|PLAK_HUM | JUP | PLAK_HUMAN_contaminant Junction plakoglobin | 3 |
| sp\|P04792\|HSPB1_HU | HSPB1 | HSPB1_HUMAN Heat shock protein beta-1 | 3 |
| sp\|P39748\|FEN1_HUM | FEN1 | FEN1_HUMAN Flap endonuclease 1 | 3 |
| sp\|Q96BK5\|PINX1_HU | PINX1 | PINX1_HUMAN PIN2/TERF1-interacting telomerase inhibitor 1 | 3 |
| sp\|P05141\|ADT2_HUM | SLC25A5 | ADT2_HUMAN ADP/ATP translocase 2 | 3 |
| sp\|Q15393\|SF3B3_HU | SF3B3 | SF3B3_HUMAN Splicing factor 3B subunit 3 | 3 |
| sp\|Q9Y2Q9\|RT28_HUM | MRPS28 | RT28_HUMAN 28S ribosomal protein S28, mitochondrial | 3 |
| sp\|P84090\|ERH_HUMA | ERH | ERH_HUMAN Enhancer of rudimentary homolog | 3 |
| sp\|P45973\|CBX5_HUM | CBX5 | CBX5_HUMAN Chromobox protein homolog 5 | 3 |
| sp\|P46087-4\|NOP2_H | NOP2 | NOP2_HUMAN Isoform 4 of Putative ribosomal RNA methyltransfe | 3 |
| sp\|Q9UHX1\|PUF60_HU | PUF60 | PUF60_HUMAN Poly(U)-binding-splicing factor PUF60 | 3 |
| sp\|P48643\|TCPE_HUM | CCT5 | TCPE_HUMAN T-complex protein 1 subunit epsilon | 3 |
| sp\|Q9Y5B9\|SP16H_HU | SUPT16H | SP16H_HUMAN FACT complex subunit SPT16 | 3 |
| sp\|P82663\|RT25_HUM | MRPS25 | RT25_HUMAN 28S ribosomal protein S25, mitochondrial | 3 |
| sp\|P43246\|MSH2_HU | MSH2 | MSH2_HUMAN DNA mismatch repair protein Msh2 | 3 |
| sp\|Q86TN4-4\|TRPT1_H | TRPT1 | TRPT1_HUMAN Isoform 4 of tRNA 2'-phosphotransferase 1 | 3 |
| sp\|Q9H9J2\|RM44_HU | MRPL44 | RM44_HUMAN 39S ribosomal protein L44, mitochondrial | 3 |
| sp\|P54819\|KAD2_HUM | AK2 | KAD2_HUMAN Adenylate kinase 2, mitochondrial | 3 |
| sp\|Q96QR8\|PURB_HU | PURB | PURB_HUMAN Transcriptional activator protein Pur-beta | 3 |
| sp\|Q8N5N7\|RM50_HU | MRPL50 | RM50_HUMAN 39S ribosomal protein L50, mitochondrial | 3 |
| sp\|P82933\|RT09_HUM | MRPS9 | RT09_HUMAN 28S ribosomal protein S9, mitochondrial | 3 |
| sp\|Q9Y5A9\|YTHD2_HU | YTHDF2 | YTHD2_HUMAN YTH domain family protein 2 | 3 |
| sp\|O43175\|SERA_HUM | PHGDH | SERA_HUMAN D-3-phosphoglycerate dehydrogenase | 3 |
| sp\|Q96T88-2\|UHRF1_H | UHRF1 | UHRF1_HUMAN Isoform 2 of E3 ubiquitin-protein ligase UHRF1 | 3 |

| sp\|Q9HC36\|RMTL1_H | RNMTL1 | RMTL1_HUMAN RNA methyltransferase-like protein 1 | 3 |
| --- | --- | --- | --- |
| sp\|Q6P2Q9\|PRP8_HU | PRPF8 | PRP8_HUMAN Pre-mRNA-processing-splicing factor 8 | 3 |
| sp\|P61981\|1433G_HU | YWHAG | 1433G_HUMAN 14-3-3 protein gamma | 3 |
| sp\|O43707\|ACTN4_HU | ACTN4 | ACTN4_HUMAN Alpha-actinin-4 | 3 |
| sp\|P29692-2\|EF1D_HU | EEF1D | EF1D_HUMAN Isoform 2 of Elongation factor 1-delta | 3 |
| sp\|P62495\|ERF1_HUM | ETF1 | ERF1_HUMAN Eukaryotic peptide chain release factor subunit 1 | 3 |
| sp\|P60900\|PSA6_HUM | PSMA6 | PSA6_HUMAN Proteasome subunit alpha type-6 | 3 |
| sp\|P46776\|RL27A_HU | RPL27A | RL27A_HUMAN 60S ribosomal protein L27a | 3 |
| sp\|Q8NBJ5\|GT251_HU | COLGALT1 | GT251_HUMAN Procollagen galactosyltransferase 1 | 3 |
| sp\|P82912\|RT11_HUM | MRPS11 | RT11_HUMAN 28S ribosomal protein S11, mitochondrial | 3 |
| sp\|P25705\|ATPA_HUM | ATP5A1 | ATPA_HUMAN ATP synthase subunit alpha, mitochondrial | 3 |
| sp\|P55769\|NH2L1_HU | NHP2L1 | NH2L1_HUMAN NHP2-like protein 1 | 3 |
| sp\|Q13428-4\|TCOF_H | TCOF1 | TCOF_HUMAN Isoform 4 of Treacle protein | 3 |
| sp\|P09429\|HMGB1_HU | HMGB1 | HMGB1_HUMAN High mobility group protein B1 | 3 |
| sp\|Q02790\|FKBP4_HU | FKBP4 | FKBP4_HUMAN Peptidyl-prolyl cis-trans isomerase FKBP4 | 3 |
| sp\|P25205\|MCM3_HU | MCM3 | MCM3_HUMAN DNA replication licensing factor MCM3 | 3 |
| sp\|O60524\|NEMF_HU | NEMF | NEMF_HUMAN Nuclear export mediator factor NEMF | 3 |
| sp\|Q8WUZ0-2\|BCL7C_ | BCL7C | BCL7C_HUMAN Isoform 2 of B-cell CLL/lymphoma 7 protein family | 3 |
| sp\|P50990\|TCPQ_HUM | CCT8 | TCPQ_HUMAN T-complex protein 1 subunit theta | 3 |
| sp\|P68400\|CSK21_HU | CSNK2A1 | CSK21_HUMAN Casein kinase II subunit alpha | 3 |
| sp\|P35268\|RL22_HUM | RPL22 | RL22_HUMAN 60S ribosomal protein L22 | 3 |
| sp\|Q8N6U8-6\|GP161_ | GPR161 | GP161_HUMAN Isoform 6 of G-protein coupled receptor 161 | 3 |
| sp\|Q13610\|PWP1_HU | PWP1 | PWP1_HUMAN Periodic tryptophan protein 1 homolog | 3 |
| sp\|P30048\|PRDX3_HU | PRDX3 | PRDX3_HUMAN Thioredoxin-dependent peroxide reductase, mitoc | 3 |
| sp\|Q96DI7\|SNR40_HU | SNRNP40 | SNR40_HUMAN U5 small nuclear ribonucleoprotein 40 kDa protein | 3 |
| sp\|P49321-3\|NASP_HU | NASP | NASP_HUMAN Isoform 3 of Nuclear autoantigenic sperm protein | 3 |
| sp\|O00571\|DDX3X_HU | DDX3X | DDX3X_HUMAN ATP-dependent RNA helicase DDX3X | 3 |
| sp\|P18847\|ATF3_HUM | ATF3 | ATF3_HUMAN Cyclic AMP-dependent transcription factor ATF-3 | 3 |
| sp\|P07437\|TBB5_HUM | TUBB | TBB5_HUMAN Tubulin beta chain | 3 |
| sp\|Q08945\|SSRP1_HU | SSRP1 | SSRP1_HUMAN FACT complex subunit SSRP1 | 3 |

| sp\|P14649\|MYL6B_HU | MYL6B | MYL6B_HUMAN Myosin light chain 6B | 3 |
| --- | --- | --- | --- |
| sp\|Q9H0U6\|RM18_HU | MRPL18 | RM18_HUMAN 39S ribosomal protein L18, mitochondrial | 3 |
| sp\|Q16576\|RBBP7_HU | RBBP7 | RBBP7_HUMAN Histone-binding protein RBBP7 | 3 |
| sp\|O00488\|ZN593_HU | ZNF593 | ZN593_HUMAN Zinc finger protein 593 | 3 |
| sp\|Q06830\|PRDX1_HU | PRDX1 | PRDX1_HUMAN Peroxiredoxin-1 | 3 |
| sp\|Q04917\|1433F_HU | YWHAH | 1433F_HUMAN 14-3-3 protein eta | 3 |
| sp\|Q9UQ35\|SRRM2_H | SRRM2 | SRRM2_HUMAN Serine/arginine repetitive matrix protein 2 | 3 |
| sp\|Q92598\|HS105_HU | HSPH1 | HS105_HUMAN Heat shock protein 105 kDa | 3 |
| sp\|P50454\|SERPH_HU | SERPINH1 | SERPH_HUMAN Serpin H1 | 3 |
| sp\|P40227\|TCPZ_HUM | CCT6A | TCPZ_HUMAN T-complex protein 1 subunit zeta | 3 |
| sp\|Q01085-2\|TIAR_HU | TIAL1 | TIAR_HUMAN Isoform 2 of Nucleolysin TIAR | 3 |
| sp\|Q15050\|RRS1_HUM | RRS1 | RRS1_HUMAN Ribosome biogenesis regulatory protein homolog | 3 |
| sp\|P63104\|1433Z_HU | YWHAZ | 1433Z_HUMAN 14-3-3 protein zeta/delta | 3 |
| sp\|Q14157-5\|UBP2L_H | UBAP2L | UBP2L_HUMAN Isoform 5 of Ubiquitin-associated protein 2-like | 3 |
| sp\|Q9H0D6\|XRN2_HU | XRN2 | XRN2_HUMAN 5'-3' exoribonuclease 2 | 3 |
| sp\|Q96A72\|MGN2_HU | MAGOHB | MGN2_HUMAN Protein mago nashi homolog 2 | 3 |
| sp\|O95433\|AHSA1_HU | AHSA1 | AHSA1_HUMAN Activator of 90 kDa heat shock protein ATPase ho | 3 |
| sp\|Q16629\|SRSF7_HU | SRSF7 | SRSF7_HUMAN Serine/arginine-rich splicing factor 7 | 3 |
| sp\|Q9UI30\|TR112_HU | TRMT112 | TR112_HUMAN tRNA methyltransferase 112 homolog | 3 |
| sp\|Q8TDI0\|CHD5_HUM | CHD5 | CHD5_HUMAN Chromodomain-helicase-DNA-binding protein 5 | 2 |
| sp\|Q9Y2R5\|RT17_HUM | MRPS17 | RT17_HUMAN 28S ribosomal protein S17, mitochondrial | 2 |
| sp\|O75643\|U520_HUM | SNRNP200 | U520_HUMAN U5 small nuclear ribonucleoprotein 200 kDa helicas | 2 |
| sp\|Q99832\|TCPH_HUM | CCT7 | TCPH_HUMAN T-complex protein 1 subunit eta | 2 |
| sp\|P50914\|RL14_HUM | RPL14 | RL14_HUMAN 60S ribosomal protein L14 | 2 |
| sp\|Q9UG63\|ABCF2_HU | ABCF2 | ABCF2_HUMAN ATP-binding cassette sub-family F member 2 | 2 |
| sp\|P55265-4\|DSRAD_H | ADAR | DSRAD_HUMAN Isoform 4 of Double-stranded RNA-specific adeno | 2 |
| sp\|P82932\|RT06_HUM | MRPS6 | RT06_HUMAN 28S ribosomal protein S6, mitochondrial | 2 |
| sp\|P05386\|RLA1_HUM | RPLP1 | RLA1_HUMAN 60S acidic ribosomal protein P1 | 2 |
| sp\|P82675\|RT05_HUM | MRPS5 | RT05_HUMAN 28S ribosomal protein S5, mitochondrial | 2 |
| sp\|P26358-2\|DNMT1_ | DNMT1 | DNMT1_HUMAN Isoform 2 of DNA (cytosine-5)-methyltransferase | 2 |

| sp\|P28072\|PSB6_HUM | PSMB6 | PSB6_HUMAN Proteasome subunit beta type-6 | 2 |
| --- | --- | --- | --- |
| sp\|Q8WUA2\|PPIL4_HU | PPIL4 | PPIL4_HUMAN Peptidyl-prolyl cis-trans isomerase-like 4 | 2 |
| sp\|Q9Y3D3\|RT16_HUM | MRPS16 | RT16_HUMAN 28S ribosomal protein S16, mitochondrial | 2 |
| sp\|Q7RTV0\|PHF5A_HU | PHF5A | PHF5A_HUMAN PHD finger-like domain-containing protein 5A | 2 |
| sp\|Q6DKI1\|RL7L_HUM | RPL7L1 | RL7L_HUMAN 60S ribosomal protein L7-like 1 | 2 |
| sp\|Q15388\|TOM20_H | TOMM20 | TOM20_HUMAN Mitochondrial import receptor subunit TOM20 ho | 2 |
| sp\|P25786-2\|PSA1_HU | PSMA1 | PSA1_HUMAN Isoform Long of Proteasome subunit alpha type-1 | 2 |
| sp\|Q15427\|SF3B4_HU | SF3B4 | SF3B4_HUMAN Splicing factor 3B subunit 4 | 2 |
| sp\|Q15185\|TEBP_HUM | PTGES3 | TEBP_HUMAN Prostaglandin E synthase 3 | 2 |
| sp\|Q9NX24\|NHP2_HU | NHP2 | NHP2_HUMAN H/ACA ribonucleoprotein complex subunit 2 | 2 |
| sp\|P28070\|PSB4_HUM | PSMB4 | PSB4_HUMAN Proteasome subunit beta type-4 | 2 |
| sp\|Q8IXM3\|RM41_HU | MRPL41 | RM41_HUMAN 39S ribosomal protein L41, mitochondrial | 2 |
| sp\|Q8IY81\|SPB1_HUM | FTSJ3 | SPB1_HUMAN pre-rRNA processing protein FTSJ3 | 2 |
| sp\|Q13595\|TRA2A_HU | TRA2A | TRA2A_HUMAN Transformer-2 protein homolog alpha | 2 |
| sp\|P02666\|CASB_BOVI | CSN2 | CASB_BOVIN_contaminant Beta-casein | 2 |
| sp\|P28066\|PSA5_HUM | PSMA5 | PSA5_HUMAN Proteasome subunit alpha type-5 | 2 |
| sp\|Q6PK04\|CC137_HU | CCDC137 | CC137_HUMAN Coiled-coil domain-containing protein 137 | 2 |
| sp\|P49458\|SRP09_HU | SRP9 | SRP09_HUMAN Signal recognition particle 9 kDa protein | 2 |
| sp\|Q96DV4\|RM38_HU | MRPL38 | RM38_HUMAN 39S ribosomal protein L38, mitochondrial | 2 |
| sp\|Q9Y2W1\|TR150_HU | THRAP3 | TR150_HUMAN Thyroid hormone receptor-associated protein 3 | 2 |
| sp\|Q8TBF4\|ZCRB1_HU | ZCRB1 | ZCRB1_HUMAN Zinc finger CCHC-type and RNA-binding motif-con | 2 |
| sp\|P00558\|PGK1_HUM | PGK1 | PGK1_HUMAN Phosphoglycerate kinase 1 | 2 |
| sp\|O95707\|RPP29_HU | POP4 | RPP29_HUMAN Ribonuclease P protein subunit p29 | 2 |
| sp\|Q86YQ8\|CPNE8_HU | CPNE8 | CPNE8_HUMAN Copine-8 | 2 |
| sp\|Q9UNZ5\|L10K_HU | C19orf53 | L10K_HUMAN Leydig cell tumor 10 kDa protein homolog | 2 |
| sp\|P53621-2\|COPA_H | COPA | COPA_HUMAN Isoform 2 of Coatomer subunit alpha | 2 |
| sp\|P41091\|IF2G_HUM | EIF2S3 | IF2G_HUMAN Eukaryotic translation initiation factor 2 subunit 3 | 2 |
| sp\|Q9BXS6\|NUSAP_HU | NUSAP1 | NUSAP_HUMAN Nucleolar and spindle-associated protein 1 | 2 |
| sp\|Q96PK6\|RBM14_HU | RBM14 | RBM14_HUMAN RNA-binding protein 14 | 2 |
| sp\|Q6FI13\|H2A2A_HU | HIST2H2AA3 | H2A2A_HUMAN Histone H2A type 2-A | 2 |

| sp\|Q9P2R3-4\|ANFY1_ | ANKFY1 | ANFY1_HUMAN Isoform 4 of Ankyrin repeat and FYVE domain-con | 2 |
| --- | --- | --- | --- |
| sp\|Q9Y6A4\|CP080_HU | C16orf80 | CP080_HUMAN UPF0468 protein C16orf80 | 2 |
| sp\|Q6DD87\|ZN787_H | ZNF787 | ZN787_HUMAN Zinc finger protein 787 | 2 |
| sp\|O15226-2\|NKRF_H | NKRF | NKRF_HUMAN Isoform 2 of NF-kappa-B-repressing factor | 2 |
| sp\|Q7Z739\|YTHD3_HU | YTHDF3 | YTHD3_HUMAN YTH domain family protein 3 | 2 |
| sp\|P07305\|H10_HUMA | H1F0 | H10_HUMAN Histone H1.0 | 2 |
| sp\|Q96SB4-3\|SRPK1_H | SRPK1 | SRPK1_HUMAN Isoform 1 of SRSF protein kinase 1 | 2 |
| tr\|K7EJB5\|K7EJB5_HUM | SNRPD2 | K7EJB5_HUMAN Small nuclear ribonucleoprotein Sm D2 | 2 |
| sp\|Q9BRT6\|LLPH_HUM | LLPH | LLPH_HUMAN Protein LLP homolog | 2 |
| sp\|Q9H2H8-2\|PPIL3_H | PPIL3 | PPIL3_HUMAN Isoform 2 of Peptidyl-prolyl cis-trans isomerase-like | 2 |
| sp\|Q13084\|RM28_HU | MRPL28 | RM28_HUMAN 39S ribosomal protein L28, mitochondrial | 2 |
| sp\|O76094\|SRP72_HU | SRP72 | SRP72_HUMAN Signal recognition particle subunit SRP72 | 2 |
| sp\|Q9UII2\|ATIF1_HUM | ATPIF1 | ATIF1_HUMAN ATPase inhibitor, mitochondrial | 2 |
| sp\|Q14151\|SAFB2_HU | SAFB2 | SAFB2_HUMAN Scaffold attachment factor B2 | 2 |
| sp\|O43660\|PLRG1_HU | PLRG1 | PLRG1_HUMAN Pleiotropic regulator 1 | 2 |
| sp\|Q02413\|DSG1_HUM | DSG1 | DSG1_HUMAN_contaminant Desmoglein-1 | 2 |
| sp\|P49327\|FAS_HUMA | FASN | FAS_HUMAN Fatty acid synthase | 2 |
| sp\|Q9HAV7\|GRPE1_HU | GRPEL1 | GRPE1_HUMAN GrpE protein homolog 1, mitochondrial | 2 |
| sp\|Q9Y5S9\|RBM8A_H | RBM8A | RBM8A_HUMAN RNA-binding protein 8A | 2 |
| sp\|O15371\|EIF3D_HU | EIF3D | EIF3D_HUMAN Eukaryotic translation initiation factor 3 subunit D | 2 |
| sp\|O75817\|POP7_HUM | POP7 | POP7_HUMAN Ribonuclease P protein subunit p20 | 2 |
| sp\|Q00325\|MPCP_HU | SLC25A3 | MPCP_HUMAN Phosphate carrier protein, mitochondrial | 2 |
| sp\|Q9H501\|ESF1_HUM | ESF1 | ESF1_HUMAN ESF1 homolog | 2 |
| sp\|Q9UGY1\|NOL12_H | NOL12 | NOL12_HUMAN Nucleolar protein 12 | 2 |
| sp\|O95983\|MBD3_HU | MBD3 | MBD3_HUMAN Methyl-CpG-binding domain protein 3 | 2 |
| sp\|P10599\|THIO_HUM | TXN | THIO_HUMAN Thioredoxin | 2 |
| sp\|O76003\|GLRX3_HU | GLRX3 | GLRX3_HUMAN Glutaredoxin-3 | 2 |
| sp\|P39687\|AN32A_HU | ANP32A | AN32A_HUMAN Acidic leucine-rich nuclear phosphoprotein 32 fam | 2 |
| sp\|Q9NW13\|RBM28_H | RBM28 | RBM28_HUMAN RNA-binding protein 28 | 2 |
| sp\|Q9BYD2\|RM09_HU | MRPL9 | RM09_HUMAN 39S ribosomal protein L9, mitochondrial | 2 |

| sp\|Q9H7B2\|RPF2_HUM | RPF2 | RPF2_HUMAN Ribosome production factor 2 homolog | 2 |
| --- | --- | --- | --- |
| sp\|Q9H7H0-3\|MET17_ | METTL17 | MET17_HUMAN Isoform 3 of Methyltransferase-like protein 17, mi | 2 |
| sp\|Q13561-2\|DCTN2_ | DCTN2 | DCTN2_HUMAN Isoform 2 of Dynactin subunit 2 | 2 |
| sp\|Q9Y5J9\|TIM8B_HU | TIMM8B | TIM8B_HUMAN Mitochondrial import inner membrane translocase | 2 |
| sp\|O60220\|TIM8A_HU | TIMM8A | TIM8A_HUMAN Mitochondrial import inner membrane translocase | 2 |
| sp\|Q9HCD5\|NCOA5_H | NCOA5 | NCOA5_HUMAN Nuclear receptor coactivator 5 | 2 |
| sp\|Q9UMY1\|NOL7_HU | NOL7 | NOL7_HUMAN Nucleolar protein 7 | 2 |
| sp\|Q6P5R6\|RL22L_HU | RPL22L1 | RL22L_HUMAN 60S ribosomal protein L22-like 1 | 2 |
| sp\|P30101\|PDIA3_HU | PDIA3 | PDIA3_HUMAN Protein disulfide-isomerase A3 | 2 |
| sp\|Q9BYD3\|RM04_HU | MRPL4 | RM04_HUMAN 39S ribosomal protein L4, mitochondrial | 2 |
| sp\|Q7Z417\|NUFP2_HU | NUFIP2 | NUFP2_HUMAN Nuclear fragile X mental retardation-interacting pr | 2 |
| sp\|Q9BUL9\|RPP25_HU | RPP25 | RPP25_HUMAN Ribonuclease P protein subunit p25 | 2 |
| sp\|P62191\|PRS4_HUM | PSMC1 | PRS4_HUMAN 26S protease regulatory subunit 4 | 2 |
| sp\|P00338-3\|LDHA_H | LDHA | LDHA_HUMAN Isoform 3 of L-lactate dehydrogenase A chain | 2 |
| sp\|P41227\|NAA10_HU | NAA10 | NAA10_HUMAN N-alpha-acetyltransferase 10 | 2 |
| sp\|Q9NY12\|GAR1_HU | GAR1 | GAR1_HUMAN H/ACA ribonucleoprotein complex subunit 1 | 2 |
| sp\|Q8TDD1-2\|DDX54_ | DDX54 | DDX54_HUMAN Isoform 2 of ATP-dependent RNA helicase DDX54 | 2 |
| sp\|Q14137\|BOP1_HUM | BOP1 | BOP1_HUMAN Ribosome biogenesis protein BOP1 | 2 |
| sp\|P49368\|TCPG_HUM | CCT3 | TCPG_HUMAN T-complex protein 1 subunit gamma | 2 |
| sp\|Q04760\|LGUL_HUM | GLO1 | LGUL_HUMAN Lactoylglutathione lyase | 2 |
| sp\|P82930\|RT34_HUM | MRPS34 | RT34_HUMAN 28S ribosomal protein S34, mitochondrial | 2 |
| sp\|Q8N983\|RM43_HU | MRPL43 | RM43_HUMAN 39S ribosomal protein L43, mitochondrial | 2 |
| sp\|O43172\|PRP4_HUM | PRPF4 | PRP4_HUMAN U4/U6 small nuclear ribonucleoprotein Prp4 | 2 |
| sp\|Q92769\|HDAC2_HU | HDAC2 | HDAC2_HUMAN Histone deacetylase 2 | 2 |
| sp\|Q9NVP1\|DDX18_H | DDX18 | DDX18_HUMAN ATP-dependent RNA helicase DDX18 | 2 |
| sp\|Q8WUA4\|TF3C2_H | GTF3C2 | TF3C2_HUMAN General transcription factor 3C polypeptide 2 | 2 |
| sp\|Q16557\|PSG3_HUM | PSG3 | PSG3_HUMAN Pregnancy-specific beta-1-glycoprotein 3 | 2 |
| sp\|P50502\|F10A1_HU | ST13 | F10A1_HUMAN Hsc70-interacting protein | 2 |
| sp\|Q8WWM7-3\|ATX2L | ATXN2L | ATX2L_HUMAN Isoform 3 of Ataxin-2-like protein | 2 |
| sp\|Q15459\|SF3A1_HU | SF3A1 | SF3A1_HUMAN Splicing factor 3A subunit 1 | 2 |

| sp\|P06753\|TPM3_HUM | TPM3 | TPM3_HUMAN Tropomyosin alpha-3 chain | 2 |
| --- | --- | --- | --- |
| sp\|P06280\|AGAL_HUM | GLA | AGAL_HUMAN Alpha-galactosidase A | 2 |
| sp\|P14854\|CX6B1_HU | COX6B1 | CX6B1_HUMAN Cytochrome c oxidase subunit 6B1 | 2 |
| sp\|Q9HB71\|CYBP_HU | CACYBP | CYBP_HUMAN Calcyclin-binding protein | 2 |
| sp\|Q9Y4C8\|RBM19_HU | RBM19 | RBM19_HUMAN Probable RNA-binding protein 19 | 2 |
| sp\|Q9NWK9\|BCD1_HU | ZNHIT6 | BCD1_HUMAN Box C/D snoRNA protein 1 | 2 |
| sp\|P26196\|DDX6_HUM | DDX6 | DDX6_HUMAN Probable ATP-dependent RNA helicase DDX6 | 2 |
| sp\|P17096-3\|HMGA1_ | HMGA1 | HMGA1_HUMAN Isoform HMG-R of High mobility group protein H | 2 |
| sp\|Q9Y295\|DRG1_HU | DRG1 | DRG1_HUMAN Developmentally-regulated GTP-binding protein 1 | 2 |
| sp\|Q93009\|UBP7_HUM | USP7 | UBP7_HUMAN Ubiquitin carboxyl-terminal hydrolase 7 | 2 |
| sp\|Q99873\|ANM1_HU | PRMT1 | ANM1_HUMAN Protein arginine N-methyltransferase 1 | 2 |
| sp\|Q99543\|DNJC2_HU | DNAJC2 | DNJC2_HUMAN DnaJ homolog subfamily C member 2 | 2 |
| sp\|P98179\|RBM3_HUM | RBM3 | RBM3_HUMAN Putative RNA-binding protein 3 | 2 |
| sp\|Q9NQ50\|RM40_HU | MRPL40 | RM40_HUMAN 39S ribosomal protein L40, mitochondrial | 2 |
| sp\|P24752\|THIL_HUM | ACAT1 | THIL_HUMAN Acetyl-CoA acetyltransferase, mitochondrial | 2 |
| sp\|P22314\|UBA1_HUM | UBA1 | UBA1_HUMAN Ubiquitin-like modifier-activating enzyme 1 | 2 |
| sp\|P27824\|CALX_HUM | CANX | CALX_HUMAN Calnexin | 2 |
| tr\|M0R2L9\|M0R2L9_H | RPS19 | M0R2L9_HUMAN 40S ribosomal protein S19 (Fragment) | 2 |
| sp\|Q9Y676\|RT18B_HU | MRPS18B | RT18B_HUMAN 28S ribosomal protein S18b, mitochondrial | 2 |
| sp\|P52209\|6PGD_HUM | PGD | 6PGD_HUMAN 6-phosphogluconate dehydrogenase, decarboxylati | 2 |
| sp\|Q96AE4-2\|FUBP1_H | FUBP1 | FUBP1_HUMAN Isoform 2 of Far upstream element-binding protei | 2 |
| sp\|Q5T653\|RM02_HU | MRPL2 | RM02_HUMAN 39S ribosomal protein L2, mitochondrial | 2 |
| sp\|Q8WUD4\|CCD12_H | CCDC12 | CCD12_HUMAN Coiled-coil domain-containing protein 12 | 2 |
| sp\|Q9Y3Y2-3\|CHTOP_ | CHTOP | CHTOP_HUMAN Isoform 2 of Chromatin target of PRMT1 protein | 2 |
| sp\|P14314\|GLU2B_HU | PRKCSH | GLU2B_HUMAN Glucosidase 2 subunit beta | 2 |
| sp\|P05114\|HMGN1_H | HMGN1 | HMGN1_HUMAN Non-histone chromosomal protein HMG-14 | 2 |
| sp\|Q9BQA1\|MEP50_H | WDR77 | MEP50_HUMAN Methylosome protein 50 | 2 |
| sp\|P02769\|ALBU_BOVI | ALB | ALBU_BOVIN_contaminant Serum albumin | 2 |
| sp\|Q9NWU5\|RM22_H | MRPL22 | RM22_HUMAN 39S ribosomal protein L22, mitochondrial | 2 |
| sp\|P16949-2\|STMN1_ | STMN1 | STMN1_HUMAN Isoform 2 of Stathmin | 2 |

| sp\|Q13162\|PRDX4_HU | PRDX4 | PRDX4_HUMAN Peroxiredoxin-4 | 2 |
| --- | --- | --- | --- |
| sp\|Q16630-2\|CPSF6_H | CPSF6 | CPSF6_HUMAN Isoform 2 of Cleavage and polyadenylation specific | 2 |
| sp\|P26583\|HMGB2_HU | HMGB2 | HMGB2_HUMAN High mobility group protein B2 | 2 |
| sp\|P50579\|MAP2_HUM | METAP2 | MAP2_HUMAN Methionine aminopeptidase 2 | 2 |
| sp\|Q96SB3\|NEB2_HUM | PPP1R9B | NEB2_HUMAN Neurabin-2 | 2 |
| sp\|P51398\|RT29_HUM | DAP3 | RT29_HUMAN 28S ribosomal protein S29, mitochondrial | 2 |
| sp\|Q9Y608\|LRRF2_HU | LRRFIP2 | LRRF2_HUMAN Leucine-rich repeat flightless-interacting protein 2 | 2 |
| tr\|Q5TBP9\|Q5TBP9_HU | LSM14B | Q5TBP9_HUMAN Protein LSM14 homolog B (Fragment) | 2 |
| sp\|O60884\|DNJA2_HU | DNAJA2 | DNJA2_HUMAN DnaJ homolog subfamily A member 2 | 1 |
| sp\|Q8WU90\|ZC3HF_H | ZC3H15 | ZC3HF_HUMAN Zinc finger CCCH domain-containing protein 15 | 1 |
| sp\|P81605\|DCD_HUM | DCD | DCD_HUMAN_contaminant Dermcidin | 1 |
| sp\|P61513\|RL37A_HU | RPL37A | RL37A_HUMAN 60S ribosomal protein L37a | 1 |
| sp\|P22061-2\|PIMT_HU | PCMT1 | PIMT_HUMAN Isoform 2 of Protein-L-isoaspartate(D-aspartate) O- | 1 |
| sp\|Q9UJV9\|DDX41_HU | DDX41 | DDX41_HUMAN Probable ATP-dependent RNA helicase DDX41 | 1 |
| sp\|P17980\|PRS6A_HU | PSMC3 | PRS6A_HUMAN 26S protease regulatory subunit 6A | 1 |
| sp\|Q86WG5\|MTMRD_ | SBF2 | MTMRD_HUMAN Myotubularin-related protein 13 | 1 |
| sp\|Q9NUL3\|STAU2_HU | STAU2 | STAU2_HUMAN Double-stranded RNA-binding protein Staufen ho | 1 |
| sp\|Q96EU6\|RRP36_HU | RRP36 | RRP36_HUMAN Ribosomal RNA processing protein 36 homolog | 1 |
| sp\|O75475\|PSIP1_HU | PSIP1 | PSIP1_HUMAN PC4 and SFRS1-interacting protein | 1 |
| sp\|Q03701\|CEBPZ_HU | CEBPZ | CEBPZ_HUMAN CCAAT/enhancer-binding protein zeta | 1 |
| sp\|Q9Y399\|RT02_HUM | MRPS2 | RT02_HUMAN 28S ribosomal protein S2, mitochondrial | 1 |
| sp\|Q9HCN8\|SDF2L_HU | SDF2L1 | SDF2L_HUMAN Stromal cell-derived factor 2-like protein 1 | 1 |
| sp\|P42766\|RL35_HUM | RPL35 | RL35_HUMAN 60S ribosomal protein L35 | 1 |
| sp\|P26639-2\|SYTC_HU | TARS | SYTC_HUMAN Isoform 2 of Threonine--tRNA ligase, cytoplasmic | 1 |
| sp\|P82914\|RT15_HUM | MRPS15 | RT15_HUMAN 28S ribosomal protein S15, mitochondrial | 1 |
| sp\|Q5SZK8\|FREM2_HU | FREM2 | FREM2_HUMAN FRAS1-related extracellular matrix protein 2 | 1 |
| sp\|P55081\|MFAP1_HU | MFAP1 | MFAP1_HUMAN Microfibrillar-associated protein 1 | 1 |
| sp\|Q9HD67\|MYO10_H | MYO10 | MYO10_HUMAN Unconventional myosin-X | 1 |
| sp\|P25787\|PSA2_HUM | PSMA2 | PSA2_HUMAN Proteasome subunit alpha type-2 | 1 |
| sp\|Q9H892-2\|TTC12_H | TTC12 | TTC12_HUMAN Isoform 2 of Tetratricopeptide repeat protein 12 | 1 |

| sp\|Q7Z2W9\|RM21_HU | MRPL21 | RM21_HUMAN 39S ribosomal protein L21, mitochondrial | 1 |
| --- | --- | --- | --- |
| sp\|P61353\|RL27_HUM | RPL27 | RL27_HUMAN 60S ribosomal protein L27 | 1 |
| tr\|Q5T5E6\|Q5T5E6_HU | KCNK1 | Q5T5E6_HUMAN Potassium channel subfamily K member 1 (Fragm | 1 |
| sp\|P06748-2\|NPM_HU | NPM1 | NPM_HUMAN Isoform 2 of Nucleophosmin | 1 |
| sp\|P49137\|MAPK2_HU | MAPKAPK2 | MAPK2_HUMAN MAP kinase-activated protein kinase 2 | 1 |
| sp\|Q01081\|U2AF1_HU | U2AF1 | U2AF1_HUMAN Splicing factor U2AF 35 kDa subunit | 1 |
| sp\|P53396\|ACLY_HUM | ACLY | ACLY_HUMAN ATP-citrate synthase | 1 |
| sp\|Q9BYC9\|RM20_HU | MRPL20 | RM20_HUMAN 39S ribosomal protein L20, mitochondrial | 1 |
| sp\|Q6P161\|RM54_HU | MRPL54 | RM54_HUMAN 39S ribosomal protein L54, mitochondrial | 1 |
| sp\|P60891\|PRPS1_HU | PRPS1 | PRPS1_HUMAN Ribose-phosphate pyrophosphokinase 1 | 1 |
| sp\|P11586\|C1TC_HUM | MTHFD1 | C1TC_HUMAN C-1-tetrahydrofolate synthase, cytoplasmic | 1 |
| sp\|Q06055-2\|AT5G2_H | ATP5G2 | AT5G2_HUMAN Isoform 2 of ATP synthase F(0) complex subunit C | 1 |
| sp\|O00541\|PESC_HUM | PES1 | PESC_HUMAN Pescadillo homolog | 1 |
| sp\|Q9BV87\|CNPD1_H | CNPPD1 | CNPD1_HUMAN Protein CNPPD1 | 1 |
| sp\|Q9H0A0\|NAT10_H | NAT10 | NAT10_HUMAN N-acetyltransferase 10 | 1 |
| sp\|Q9UNX3\|RL26L_HU | RPL26L1 | RL26L_HUMAN 60S ribosomal protein L26-like 1 | 1 |
| sp\|Q08J23\|NSUN2_HU | NSUN2 | NSUN2_HUMAN tRNA (cytosine(34)-C(5))-methyltransferase | 1 |
| sp\|P49720\|PSB3_HUM | PSMB3 | PSB3_HUMAN Proteasome subunit beta type-3 | 1 |
| sp\|Q9Y3C1-2\|NOP16_ | NOP16 | NOP16_HUMAN Isoform 2 of Nucleolar protein 16 | 1 |
| sp\|Q3MHD2-2\|LSM12 | LSM12 | LSM12_HUMAN Isoform 2 of Protein LSM12 homolog | 1 |
| sp\|P30086\|PEBP1_HU | PEBP1 | PEBP1_HUMAN Phosphatidylethanolamine-binding protein 1 | 1 |
| sp\|P30084\|ECHM_HU | ECHS1 | ECHM_HUMAN Enoyl-CoA hydratase, mitochondrial | 1 |
| sp\|P63173\|RL38_HUM | RPL38 | RL38_HUMAN 60S ribosomal protein L38 | 1 |
| sp\|P20042\|IF2B_HUMA | EIF2S2 | IF2B_HUMAN Eukaryotic translation initiation factor 2 subunit 2 | 1 |
| sp\|Q8N608-3\|DPP10_ | DPP10 | DPP10_HUMAN Isoform 3 of Inactive dipeptidyl peptidase 10 | 1 |
| sp\|P62891\|RL39_HUM | RPL39 | RL39_HUMAN 60S ribosomal protein L39 | 1 |
| sp\|O95232\|LC7L3_HU | LUC7L3 | LC7L3_HUMAN Luc7-like protein 3 | 1 |
| sp\|P47756-2\|CAPZB_H | CAPZB | CAPZB_HUMAN Isoform 2 of F-actin-capping protein subunit beta | 1 |
| sp\|Q5HYI8\|RABL3_HU | RABL3 | RABL3_HUMAN Rab-like protein 3 | 1 |
| sp\|P82673\|RT35_HUM | MRPS35 | RT35_HUMAN 28S ribosomal protein S35, mitochondrial | 1 |

| sp\|Q8WTT2\|NOC3L_H | NOC3L | NOC3L_HUMAN Nucleolar complex protein 3 homolog | 1 |
| --- | --- | --- | --- |
| sp\|Q8IWS0\|PHF6_HUM | PHF6 | PHF6_HUMAN PHD finger protein 6 | 1 |
| sp\|Q8WVM0\|TFB1M_H | TFB1M | TFB1M_HUMAN Dimethyladenosine transferase 1, mitochondrial | 1 |
| sp\|Q9GZS1\|RPA49_HU | POLR1E | RPA49_HUMAN DNA-directed RNA polymerase I subunit RPA49 | 1 |
| sp\|Q9Y421\|FA32A_HU | FAM32A | FA32A_HUMAN Protein FAM32A | 1 |
| sp\|Q4U2R6\|RM51_HU | MRPL51 | RM51_HUMAN 39S ribosomal protein L51, mitochondrial | 1 |
| sp\|Q8N4Q1-2\|MIA40_ | CHCHD4 | MIA40_HUMAN Isoform 2 of Mitochondrial intermembrane space i | 1 |
| sp\|P49736\|MCM2_HU | MCM2 | MCM2_HUMAN DNA replication licensing factor MCM2 | 1 |
| tr\|H0YDD8\|H0YDD8_H | RPLP2 | H0YDD8_HUMAN 60S acidic ribosomal protein P2 (Fragment) | 1 |
| sp\|Q7Z333-4\|SETX_HU | SETX | SETX_HUMAN Isoform 4 of Probable helicase senataxin | 1 |
| sp\|Q14571\|ITPR2_HU | ITPR2 | ITPR2_HUMAN Inositol 1,4,5-trisphosphate receptor type 2 | 1 |
| sp\|Q99547\|MPH6_HU | MPHOSPH6 | MPH6_HUMAN M-phase phosphoprotein 6 | 1 |
| sp\|Q9Y239\|NOD1_HU | NOD1 | NOD1_HUMAN Nucleotide-binding oligomerization domain-contai | 1 |
| tr\|H7C1R8\|H7C1R8_H | SLC22A5 | H7C1R8_HUMAN Solute carrier family 22 member 5 (Fragment) | 1 |
| sp\|P25789\|PSA4_HUM | PSMA4 | PSA4_HUMAN Proteasome subunit alpha type-4 | 1 |
| sp\|Q05519\|SRS11_HU | SRSF11 | SRS11_HUMAN Serine/arginine-rich splicing factor 11 | 1 |
| sp\|Q9BV90\|SNR25_HU | SNRNP25 | SNR25_HUMAN U11/U12 small nuclear ribonucleoprotein 25 kDa p | 1 |
| sp\|P25398\|RS12_HUM | RPS12 | RS12_HUMAN 40S ribosomal protein S12 | 1 |
| sp\|Q13017\|RHG05_HU | ARHGAP5 | RHG05_HUMAN Rho GTPase-activating protein 5 | 1 |
| sp\|Q86U86\|PB1_HUM | PBRM1 | PB1_HUMAN Protein polybromo-1 | 1 |
| sp\|P78406\|RAE1L_HU | RAE1 | RAE1L_HUMAN mRNA export factor | 1 |
| sp\|Q9Y3F4\|STRAP_HU | STRAP | STRAP_HUMAN Serine-threonine kinase receptor-associated protei | 1 |
| sp\|Q8NE71\|ABCF1_HU | ABCF1 | ABCF1_HUMAN ATP-binding cassette sub-family F member 1 | 1 |
| sp\|Q14004\|CDK13_HU | CDK13 | CDK13_HUMAN Cyclin-dependent kinase 13 | 1 |
| sp\|Q16658\|FSCN1_HU | FSCN1 | FSCN1_HUMAN Fascin | 1 |
| sp\|P54105\|ICLN_HUM | CLNS1A | ICLN_HUMAN Methylosome subunit pICln | 1 |
| sp\|Q9GZR7\|DDX24_H | DDX24 | DDX24_HUMAN ATP-dependent RNA helicase DDX24 | 1 |
| sp\|Q9Y262\|EIF3L_HUM | EIF3L | EIF3L_HUMAN Eukaryotic translation initiation factor 3 subunit L | 1 |
| sp\|Q8N100\|ATOH7_H | ATOH7 | ATOH7_HUMAN Protein atonal homolog 7 | 1 |
| sp\|O60783\|RT14_HUM | MRPS14 | RT14_HUMAN 28S ribosomal protein S14, mitochondrial | 1 |

| sp\|O75340\|PDCD6_HU | PDCD6 | PDCD6_HUMAN Programmed cell death protein 6 | 1 |
| --- | --- | --- | --- |
| sp\|Q16740\|CLPP_HUM | CLPP | CLPP_HUMAN Putative ATP-dependent Clp protease proteolytic su | 1 |
| sp\|Q9Y2R9\|RT07_HUM | MRPS7 | RT07_HUMAN 28S ribosomal protein S7, mitochondrial | 1 |
| sp\|Q8WZ64\|ARAP2_H | ARAP2 | ARAP2_HUMAN Arf-GAP with Rho-GAP domain, ANK repeat and P | 1 |
| sp\|Q1ED39\|KNOP1_H | KNOP1 | KNOP1_HUMAN Lysine-rich nucleolar protein 1 | 1 |
| sp\|P20618\|PSB1_HUM | PSMB1 | PSB1_HUMAN Proteasome subunit beta type-1 | 1 |
| sp\|Q68CQ4\|DIEXF_HU | DIEXF | DIEXF_HUMAN Digestive organ expansion factor homolog | 1 |
| sp\|P17480\|UBF1_HUM | UBTF | UBF1_HUMAN Nucleolar transcription factor 1 | 1 |
| sp\|Q8TF09\|DLRB2_HU | DYNLRB2 | DLRB2_HUMAN Dynein light chain roadblock-type 2 | 1 |
| sp\|Q9H6Y2\|WDR55_H | WDR55 | WDR55_HUMAN WD repeat-containing protein 55 | 1 |
| sp\|Q13642\|FHL1_HUM | FHL1 | FHL1_HUMAN Four and a half LIM domains protein 1 | 1 |
| sp\|P05412\|JUN_HUMA | JUN | JUN_HUMAN Transcription factor AP-1 | 1 |
| sp\|P56537\|IF6_HUMA | EIF6 | IF6_HUMAN Eukaryotic translation initiation factor 6 | 1 |
| sp\|P19174-2\|PLCG1_H | PLCG1 | PLCG1_HUMAN Isoform 2 of 1-phosphatidylinositol 4,5-bisphosph | 1 |
| sp\|O95218\|ZRAB2_HU | ZRANB2 | ZRAB2_HUMAN Zinc finger Ran-binding domain-containing protein | 1 |
| tr\|E9PRG8\|E9PRG8_HU | C11orf48 | E9PRG8_HUMAN Uncharacterized protein C11orf48 | 1 |
| sp\|Q8IYS0\|GRM1C_HU | GRAMD1C | GRM1C_HUMAN GRAM domain-containing protein 1C | 1 |
| sp\|Q15435\|PP1R7_HU | PPP1R7 | PP1R7_HUMAN Protein phosphatase 1 regulatory subunit 7 | 1 |
| sp\|Q9BYG3\|MK67I_HU | NIFK | MK67I_HUMAN MKI67 FHA domain-interacting nucleolar phospho | 1 |
| sp\|P61204\|ARF3_HUM | ARF3 | ARF3_HUMAN ADP-ribosylation factor 3 | 1 |
| sp\|Q9P035\|HACD3_HU | PTPLAD1 | HACD3_HUMAN Very-long-chain (3R)-3-hydroxyacyl-[acyl-carrier p | 1 |
| sp\|Q9ULX3\|NOB1_HU | NOB1 | NOB1_HUMAN RNA-binding protein NOB1 | 1 |
| sp\|Q96MG7\|MAGG1_ | NDNL2 | MAGG1_HUMAN Melanoma-associated antigen G1 | 1 |
| sp\|Q00839-2\|HNRPU_ | HNRNPU | HNRPU_HUMAN Isoform Short of Heterogeneous nuclear ribonucl | 1 |
| sp\|Q96L73\|NSD1_HU | NSD1 | NSD1_HUMAN Histone-lysine N-methyltransferase, H3 lysine-36 an | 1 |
| sp\|P23193\|TCEA1_HU | TCEA1 | TCEA1_HUMAN Transcription elongation factor A protein 1 | 1 |
| sp\|P52597\|HNRPF_HU | HNRNPF | HNRPF_HUMAN Heterogeneous nuclear ribonucleoprotein F | 1 |
| sp\|P18754-2\|RCC1_HU | RCC1 | RCC1_HUMAN Isoform 2 of Regulator of chromosome condensatio | 1 |
| sp\|Q9HCK1\|ZDBF2_HU | ZDBF2 | ZDBF2_HUMAN DBF4-type zinc finger-containing protein 2 | 1 |
| sp\|Q99819\|GDIR3_HU | ARHGDIG | GDIR3_HUMAN Rho GDP-dissociation inhibitor 3 | 1 |

| sp\|Q9NYK5-2\|RM39_H | MRPL39 | RM39_HUMAN Isoform 2 of 39S ribosomal protein L39, mitochond | 1 |
| --- | --- | --- | --- |
| sp\|P62273-2\|RS29_HU | RPS29 | RS29_HUMAN Isoform 2 of 40S ribosomal protein S29 | 1 |
| sp\|Q9GZL7\|WDR12_H | WDR12 | WDR12_HUMAN Ribosome biogenesis protein WDR12 | 1 |
| sp\|P10515\|ODP2_HUM | DLAT | ODP2_HUMAN Dihydrolipoyllysine-residue acetyltransferase compo | 1 |
| sp\|Q9Y5L4\|TIM13_HU | TIMM13 | TIM13_HUMAN Mitochondrial import inner membrane translocase | 1 |
| sp\|Q9GZS3\|WDR61_H | WDR61 | WDR61_HUMAN WD repeat-containing protein 61 | 1 |
| sp\|Q08378\|GOGA3_H | GOLGA3 | GOGA3_HUMAN Golgin subfamily A member 3 | 1 |
| sp\|P60228\|EIF3E_HUM | EIF3E | EIF3E_HUMAN Eukaryotic translation initiation factor 3 subunit E | 1 |
| sp\|Q8NEM8-2\|CBPC3_ | AGBL3 | CBPC3_HUMAN Isoform 2 of Cytosolic carboxypeptidase 3 | 1 |
| sp\|A8MT70\|ZBBX_HU | ZBBX | ZBBX_HUMAN Zinc finger B-box domain-containing protein 1 | 1 |
| sp\|P84243\|H33_HUMA | H3F3A | H33_HUMAN Histone H3.3 | 1 |
| sp\|P47813\|IF1AX_HUM | EIF1AX | IF1AX_HUMAN Eukaryotic translation initiation factor 1A, X-chromo | 1 |
| sp\|Q13123\|RED_HUM | IK | RED_HUMAN Protein Red | 1 |
| sp\|Q7Z2Z2\|ETUD1_HU | EFTUD1 | ETUD1_HUMAN Elongation factor Tu GTP-binding domain-containi | 1 |
| sp\|Q99961\|SH3G1_HU | SH3GL1 | SH3G1_HUMAN Endophilin-A2 | 1 |
| sp\|Q9P0W2\|HM20B_H | HMG20B | HM20B_HUMAN SWI/SNF-related matrix-associated actin-depende | 1 |
| sp\|Q9P258\|RCC2_HUM | RCC2 | RCC2_HUMAN Protein RCC2 | 1 |
| sp\|P83876\|TXN4A_HU | TXNL4A | TXN4A_HUMAN Thioredoxin-like protein 4A | 1 |
| sp\|P49721\|PSB2_HUM | PSMB2 | PSB2_HUMAN Proteasome subunit beta type-2 | 1 |
| sp\|Q04323-2\|UBXN1_ | UBXN1 | UBXN1_HUMAN Isoform 2 of UBX domain-containing protein 1 | 1 |
| sp\|P41218\|MNDA_HU | MNDA | MNDA_HUMAN Myeloid cell nuclear differentiation antigen | 1 |
| sp\|P30044\|PRDX5_HU | PRDX5 | PRDX5_HUMAN Peroxiredoxin-5, mitochondrial | 1 |
| sp\|Q16186\|ADRM1_H | ADRM1 | ADRM1_HUMAN Proteasomal ubiquitin receptor ADRM1 | 1 |
| sp\|P82979\|SARNP_HU | SARNP | SARNP_HUMAN SAP domain-containing ribonucleoprotein | 1 |
| sp\|P21127\|CD11B_HU | CDK11B | CD11B_HUMAN Cyclin-dependent kinase 11B | 1 |
| sp\|P12268\|IMDH2_HU | IMPDH2 | IMDH2_HUMAN Inosine-5'-monophosphate dehydrogenase 2 | 1 |
| tr\|Q5STZ8\|Q5STZ8_HU | ABCF1 | Q5STZ8_HUMAN ATP-binding cassette sub-family F member 1 (Fra | 1 |
| sp\|Q14331\|FRG1_HUM | FRG1 | FRG1_HUMAN Protein FRG1 | 1 |
| sp\|Q9UN86-2\|G3BP2_ | G3BP2 | G3BP2_HUMAN Isoform B of Ras GTPase-activating protein-bindin | 1 |
| sp\|Q12874\|SF3A3_HU | SF3A3 | SF3A3_HUMAN Splicing factor 3A subunit 3 | 1 |

| sp\|P49406\|RM19_HUM | MRPL19 | RM19_HUMAN 39S ribosomal protein L19, mitochondrial | 1 |
| --- | --- | --- | --- |
| sp\|O15078\|CE290_HU | CEP290 | CE290_HUMAN Centrosomal protein of 290 kDa | 1 |
| sp\|P67870\|CSK2B_HU | CSNK2B | CSK2B_HUMAN Casein kinase II subunit beta | 1 |
| sp\|Q8ND82\|Z280C_HU | ZNF280C | Z280C_HUMAN Zinc finger protein 280C | 1 |
| sp\|P61457\|PHS_HUMA | PCBD1 | PHS_HUMAN Pterin-4-alpha-carbinolamine dehydratase | 1 |
| sp\|Q96SI9\|STRBP_HU | STRBP | STRBP_HUMAN Spermatid perinuclear RNA-binding protein | 1 |
| sp\|Q9UJZ1\|STML2_HU | STOML2 | STML2_HUMAN Stomatin-like protein 2, mitochondrial | 1 |
| sp\|Q9NRX4\|PHP14_HU | PHPT1 | PHP14_HUMAN 14 kDa phosphohistidine phosphatase | 1 |
| sp\|Q15572\|TAF1C_HU | TAF1C | TAF1C_HUMAN TATA box-binding protein-associated factor RNA p | 1 |
| sp\|P62158\|CALM_HUM | CALM1 | CALM_HUMAN Calmodulin | 1 |
| sp\|Q13370\|PDE3B_HU | PDE3B | PDE3B_HUMAN cGMP-inhibited 3',5'-cyclic phosphodiesterase B | 1 |
| sp\|P60983\|GMFB_HUM | GMFB | GMFB_HUMAN Glia maturation factor beta | 1 |
| sp\|Q7L014\|DDX46_HU | DDX46 | DDX46_HUMAN Probable ATP-dependent RNA helicase DDX46 | 1 |
| sp\|P62306\|RUXF_HUM | SNRPF | RUXF_HUMAN Small nuclear ribonucleoprotein F | 1 |
| sp\|P51659\|DHB4_HUM | HSD17B4 | DHB4_HUMAN Peroxisomal multifunctional enzyme type 2 | 1 |
| sp\|Q9H8G2\|CAAP1_H | CAAP1 | CAAP1_HUMAN Caspase activity and apoptosis inhibitor 1 | 1 |
| sp\|O15372\|EIF3H_HU | EIF3H | EIF3H_HUMAN Eukaryotic translation initiation factor 3 subunit H | 1 |
| sp\|Q14152\|EIF3A_HUM | EIF3A | EIF3A_HUMAN Eukaryotic translation initiation factor 3 subunit A | 1 |
| sp\|Q9H7E2-3\|TDRD3_ | TDRD3 | TDRD3_HUMAN Isoform 3 of Tudor domain-containing protein 3 | 1 |
| sp\|Q4V328\|GRAP1_HU | GRIPAP1 | GRAP1_HUMAN GRIP1-associated protein 1 | 1 |
| sp\|P30049\|ATPD_HUM | ATP5D | ATPD_HUMAN ATP synthase subunit delta, mitochondrial | 1 |
| sp\|Q9H0E9\|BRD8_HU | BRD8 | BRD8_HUMAN Bromodomain-containing protein 8 | 1 |
| sp\|P78346-2\|RPP30_H | RPP30 | RPP30_HUMAN Isoform 2 of Ribonuclease P protein subunit p30 | 1 |
| sp\|Q96P63\|SPB12_HU | SERPINB12 | SPB12_HUMAN_contaminant Serpin B12 | 1 |
| sp\|P25788\|PSA3_HUM | PSMA3 | PSA3_HUMAN Proteasome subunit alpha type-3 | 1 |
| sp\|Q15906-2\|VPS72_H | VPS72 | VPS72_HUMAN Isoform 2 of Vacuolar protein sorting-associated p | 1 |
| sp\|P49756\|RBM25_HU | RBM25 | RBM25_HUMAN RNA-binding protein 25 | 1 |
| sp\|Q86WX3\|AROS_HU | RPS19BP1 | AROS_HUMAN Active regulator of SIRT1 | 1 |
| sp\|Q7L3S4\|ZN771_HU | ZNF771 | ZN771_HUMAN Zinc finger protein 771 | 1 |
| sp\|Q5TBB1\|RNH2B_HU | RNASEH2B | RNH2B_HUMAN Ribonuclease H2 subunit B | 1 |

| sp\|P63279\|UBC9_HUM | UBE2I | UBC9_HUMAN SUMO-conjugating enzyme UBC9 | 1 |
| --- | --- | --- | --- |
| sp\|O75436\|VP26A_HU | VPS26A | VP26A_HUMAN Vacuolar protein sorting-associated protein 26A | 1 |
| sp\|Q16864-2\|VATF_HU | ATP6V1F | VATF_HUMAN Isoform 2 of V-type proton ATPase subunit F | 1 |
| sp\|Q66PJ3\|AR6P4_HU | ARL6IP4 | AR6P4_HUMAN ADP-ribosylation factor-like protein 6-interacting p | 1 |
| sp\|Q9NWT8\|AKIP_HU | AURKAIP1 | AKIP_HUMAN Aurora kinase A-interacting protein | 1 |
| sp\|P46013\|KI67_HUM | MKI67 | KI67_HUMAN Antigen KI-67 | 1 |
| sp\|Q92541\|RTF1_HUM | RTF1 | RTF1_HUMAN RNA polymerase-associated protein RTF1 homolog | 1 |
| sp\|P02768\|ALBU_HUM | ALB | ALBU_HUMAN_contaminant Serum albumin | 1 |
| sp\|Q9UKX7\|NUP50_H | NUP50 | NUP50_HUMAN Nuclear pore complex protein Nup50 | 1 |
| sp\|O43809\|CPSF5_HU | NUDT21 | CPSF5_HUMAN Cleavage and polyadenylation specificity factor sub | 1 |
| tr\|M0QZM1\|M0QZM1 | HNRNPM | M0QZM1_HUMAN Heterogeneous nuclear ribonucleoprotein M (Fr | 1 |
| sp\|O00178\|GTPB1_HU | GTPBP1 | GTPB1_HUMAN GTP-binding protein 1 | 1 |
| sp\|Q9NW07\|ZN358_H | ZNF358 | ZN358_HUMAN Zinc finger protein 358 | 1 |
| sp\|O75390\|CISY_HUM | CS | CISY_HUMAN Citrate synthase, mitochondrial | 1 |
| sp\|Q9BRJ7\|SDOS_HUM | NUDT16L1 | SDOS_HUMAN Protein syndesmos | 1 |
| sp\|Q9NVS2-2\|RT18A_ | MRPS18A | RT18A_HUMAN Isoform 2 of 28S ribosomal protein S18a, mitocho | 1 |
| sp\|Q96KQ4\|ASPP1_HU | PPP1R13B | ASPP1_HUMAN Apoptosis-stimulating of p53 protein 1 | 1 |
| sp\|O95400\|CD2B2_HU | CD2BP2 | CD2B2_HUMAN CD2 antigen cytoplasmic tail-binding protein 2 | 1 |
| sp\|Q9BQC6\|RT63_HU | MRP63 | RT63_HUMAN Ribosomal protein 63, mitochondrial | 1 |
| sp\|Q12931\|TRAP1_HU | TRAP1 | TRAP1_HUMAN Heat shock protein 75 kDa, mitochondrial | 1 |
| sp\|Q9BXP5\|SRRT_HUM | SRRT | SRRT_HUMAN Serrate RNA effector molecule homolog | 1 |
| sp\|Q6DN03\|H2B2C_H | HIST2H2BC | H2B2C_HUMAN Putative histone H2B type 2-C | 1 |
| sp\|Q8WW12\|PCNP_H | PCNP | PCNP_HUMAN PEST proteolytic signal-containing nuclear protein | 1 |
| sp\|O00469-2\|PLOD2_ | PLOD2 | PLOD2_HUMAN Isoform 2 of Procollagen-lysine,2-oxoglutarate 5-d | 1 |
| sp\|Q00005-7\|2ABB_H | PPP2R2B | 2ABB_HUMAN Isoform 7 of Serine/threonine-protein phosphatase | 1 |
| sp\|P38117-2\|ETFB_HU | ETFB | ETFB_HUMAN Isoform 2 of Electron transfer flavoprotein subunit b | 1 |
| sp\|Q9Y3I0\|RTCB_HUM | RTCB | RTCB_HUMAN tRNA-splicing ligase RtcB homolog | 1 |
| sp\|Q99733\|NP1L4_HU | NAP1L4 | NP1L4_HUMAN Nucleosome assembly protein 1-like 4 | 1 |
| sp\|Q9HD34\|LYRM4_H | LYRM4 | LYRM4_HUMAN LYR motif-containing protein 4 | 1 |
| sp\|Q9UL40\|ZN346_HU | ZNF346 | ZN346_HUMAN Zinc finger protein 346 | 1 |

| sp\|Q15527\|SURF2_HU | SURF2 | SURF2_HUMAN Surfeit locus protein 2 | 1 |
| --- | --- | --- | --- |
| sp\|Q14980\|NUMA1_H | NUMA1 | NUMA1_HUMAN Nuclear mitotic apparatus protein 1 | 1 |
| sp\|Q96EP5\|DAZP1_HU | DAZAP1 | DAZP1_HUMAN DAZ-associated protein 1 | 1 |
| sp\|Q9UFW8\|CGBP1_H | CGGBP1 | CGBP1_HUMAN CGG triplet repeat-binding protein 1 | 1 |
| sp\|Q8IXB1\|DJC10_HU | DNAJC10 | DJC10_HUMAN DnaJ homolog subfamily C member 10 | 1 |
| sp\|P39656\|OST48_HU | DDOST | OST48_HUMAN Dolichyl-diphosphooligosaccharide--protein glycos | 1 |
| sp\|P68402\|PA1B2_HU | PAFAH1B2 | PA1B2_HUMAN Platelet-activating factor acetylhydrolase IB subuni | 1 |
| sp\|P82664\|RT10_HUM | MRPS10 | RT10_HUMAN 28S ribosomal protein S10, mitochondrial | 1 |
| sp\|Q15370-2\|ELOB_H | TCEB2 | ELOB_HUMAN Isoform 2 of Transcription elongation factor B polyp | 1 |
| sp\|Q9BQ75\|CMS1_HU | CMSS1 | CMS1_HUMAN Protein CMSS1 | 1 |
| sp\|Q92945\|FUBP2_HU | KHSRP | FUBP2_HUMAN Far upstream element-binding protein 2 | 1 |
| sp\|Q9UKV8\|AGO2_HU | AGO2 | AGO2_HUMAN Protein argonaute-2 | 1 |
| sp\|Q9HBI5\|CC014_HU | C3orf14 | CC014_HUMAN Uncharacterized protein C3orf14 | 1 |
| sp\|Q9Y266\|NUDC_HU | NUDC | NUDC_HUMAN Nuclear migration protein nudC | 1 |
| sp\|P09936\|UCHL1_HU | UCHL1 | UCHL1_HUMAN Ubiquitin carboxyl-terminal hydrolase isozyme L1 | 1 |
| sp\|P40925-3\|MDHC_H | MDH1 | MDHC_HUMAN Isoform 3 of Malate dehydrogenase, cytoplasmic | 1 |
| sp\|O15479\|MAGB2_H | MAGEB2 | MAGB2_HUMAN Melanoma-associated antigen B2 | 1 |
| sp\|P02662\|CASA1_BO | CSN1S1 | CASA1_BOVIN_contaminant Alpha-S1-casein | 1 |
| sp\|Q08380\|LG3BP_HU | LGALS3BP | LG3BP_HUMAN Galectin-3-binding protein | 1 |
| sp\|Q9P016\|THYN1_HU | THYN1 | THYN1_HUMAN Thymocyte nuclear protein 1 | 1 |
| sp\|Q96K17\|BT3L4_HU | BTF3L4 | BT3L4_HUMAN Transcription factor BTF3 homolog 4 | 1 |
| sp\|O00273\|DFFA_HUM | DFFA | DFFA_HUMAN DNA fragmentation factor subunit alpha | 1 |
| sp\|P06493\|CDK1_HUM | CDK1 | CDK1_HUMAN Cyclin-dependent kinase 1 | 1 |
| sp\|Q92665\|RT31_HUM | MRPS31 | RT31_HUMAN 28S ribosomal protein S31, mitochondrial | 1 |
| sp\|Q9BXV9\|CN142_HU | C14orf142 | CN142_HUMAN Uncharacterized protein C14orf142 | 1 |
| sp\|Q9NQG5\|RPR1B_H | RPRD1B | RPR1B_HUMAN Regulation of nuclear pre-mRNA domain-containin | 1 |
| sp\|P49006\|MRP_HUM | MARCKSL1 | MRP_HUMAN MARCKS-related protein | 1 |
| sp\|Q15008\|PSMD6_HU | PSMD6 | PSMD6_HUMAN 26S proteasome non-ATPase regulatory subunit 6 | 1 |
| sp\|Q9BYD1\|RM13_HU | MRPL13 | RM13_HUMAN 39S ribosomal protein L13, mitochondrial | 1 |
| sp\|P12763\|FETUA_BOV | AHSG | FETUA_BOVIN_contaminant Alpha-2-HS-glycoprotein | 1 |

| sp\|Q9H4W6\|COE3_HU | EBF3 | COE3_HUMAN Transcription factor COE3 | 1 |
| --- | --- | --- | --- |
| sp\|Q8TBZ6\|TM10A_HU | TRMT10A | TM10A_HUMAN tRNA methyltransferase 10 homolog A | 1 |
| sp\|O14654\|IRS4_HUM | IRS4 | IRS4_HUMAN Insulin receptor substrate 4 | 1 |
| sp\|P02663\|CASA2_BO | CSN1S2 | CASA2_BOVIN_contaminant Alpha-S2-casein | 1 |
| sp\|P26038\|MOES_HU | MSN | MOES_HUMAN Moesin | 1 |
| sp\|Q9NTJ3\|SMC4_HU | SMC4 | SMC4_HUMAN Structural maintenance of chromosomes protein 4 | 1 |
| sp\|Q9P2H3\|IFT80_HU | IFT80 | IFT80_HUMAN Intraflagellar transport protein 80 homolog | 1 |
| sp\|Q9BVJ6\|UT14A_HU | UTP14A | UT14A_HUMAN U3 small nucleolar RNA-associated protein 14 hom | 1 |
| sp\|Q8NEF9\|SRFB1_HU | SRFBP1 | SRFB1_HUMAN Serum response factor-binding protein 1 | 1 |
| sp\|P55884-2\|EIF3B_HU | EIF3B | EIF3B_HUMAN Isoform 2 of Eukaryotic translation initiation factor | 1 |
| sp\|P35030\|TRY3_HUM | PRSS3 | TRY3_HUMAN Trypsin-3 | 1 |
| sp\|P55036\|PSMD4_HU | PSMD4 | PSMD4_HUMAN 26S proteasome non-ATPase regulatory subunit 4 | 1 |
| sp\|Q13813-2\|SPTN1_H | SPTAN1 | SPTN1_HUMAN Isoform 2 of Spectrin alpha chain, non-erythrocytic | 1 |
| sp\|Q9Y2S7\|PDIP2_HU | POLDIP2 | PDIP2_HUMAN Polymerase delta-interacting protein 2 | 1 |
| sp\|Q9UHB9\|SRP68_HU | SRP68 | SRP68_HUMAN Signal recognition particle subunit SRP68 | 1 |
| sp\|Q01082\|SPTB2_HU | SPTBN1 | SPTB2_HUMAN Spectrin beta chain, non-erythrocytic 1 | 1 |
| sp\|Q15369\|ELOC_HUM | TCEB1 | ELOC_HUMAN Transcription elongation factor B polypeptide 1 | 1 |
| sp\|Q9Y3B7\|RM11_HU | MRPL11 | RM11_HUMAN 39S ribosomal protein L11, mitochondrial | 1 |
| sp\|Q16540\|RM23_HU | MRPL23 | RM23_HUMAN 39S ribosomal protein L23, mitochondrial | 1 |
| sp\|Q9UJU6-3\|DBNL_H | DBNL | DBNL_HUMAN Isoform 3 of Drebrin-like protein | 1 |
| sp\|O75525\|KHDR3_HU | KHDRBS3 | KHDR3_HUMAN KH domain-containing, RNA-binding, signal transd | 1 |
| sp\|Q01105\|SET_HUMA | SET | SET_HUMAN Protein SET | 1 |
| sp\|Q7Z7F7-2\|RM55_H | MRPL55 | RM55_HUMAN Isoform 2 of 39S ribosomal protein L55, mitochond | 1 |
| sp\|Q1KMD3\|HNRL2_H | HNRNPUL2 | HNRL2_HUMAN Heterogeneous nuclear ribonucleoprotein U-like p | 1 |
| sp\|Q13442\|HAP28_HU | PDAP1 | HAP28_HUMAN 28 kDa heat- and acid-stable phosphoprotein | 1 |
| sp\|P27816\|MAP4_HUM | MAP4 | MAP4_HUMAN Microtubule-associated protein 4 | 1 |
| sp\|Q13405\|RM49_HU | MRPL49 | RM49_HUMAN 39S ribosomal protein L49, mitochondrial | 1 |

## Figure S1


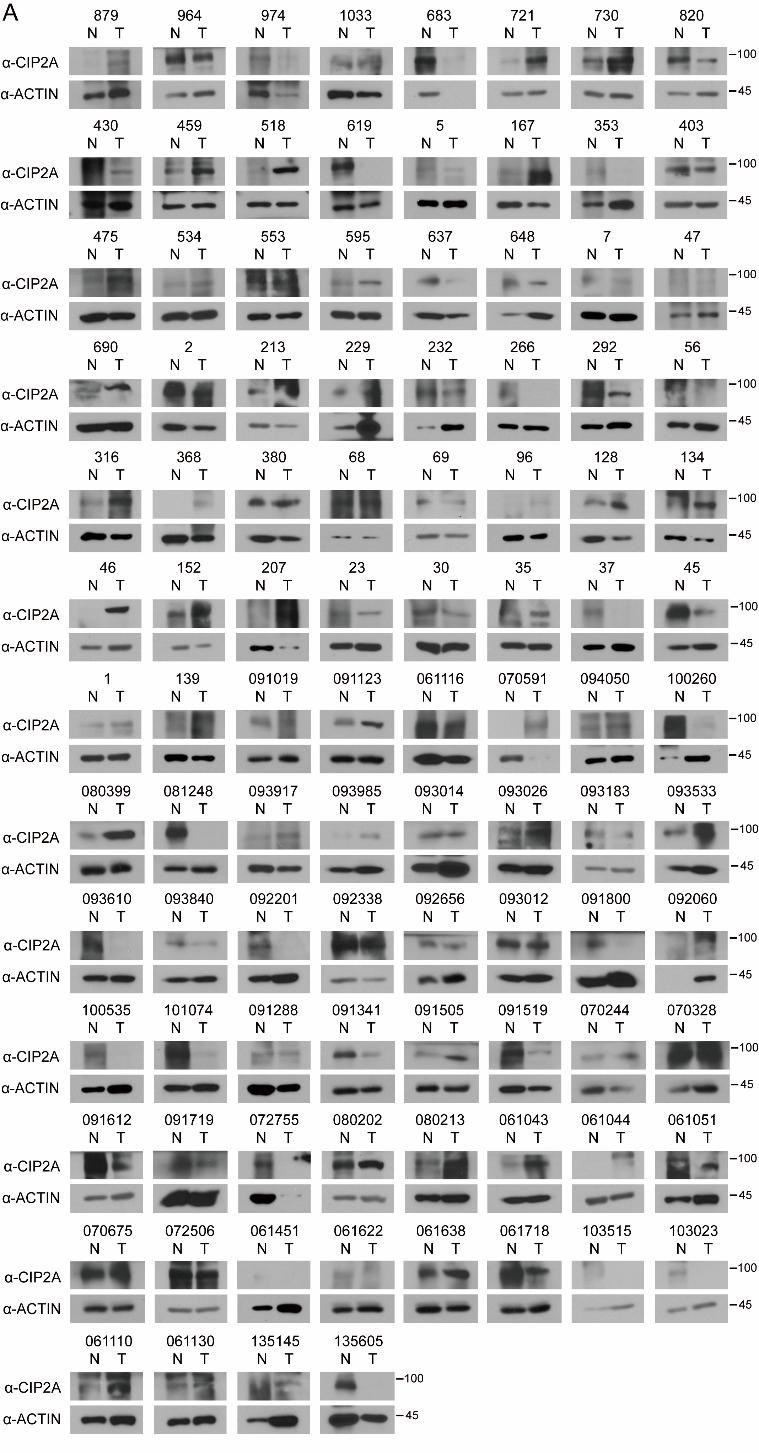


### Supplementary Figure 1. The protein levels of CIP2A in lung cancer patients’ normal and tumor tissues.

1. Western blot results in 100 paired adjacent normal lung tissues (N) and tumor tissues (T). The numbers on each blot are the serial number for patient identification.

## Figure S2


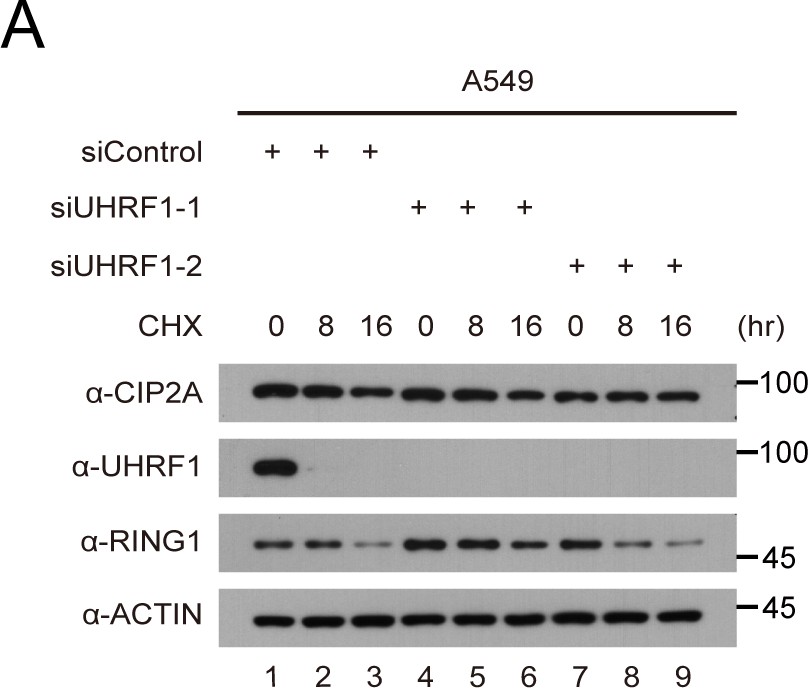


### Supplementary Figure 2. The effect of UHRF1 knockdown on the half-life of CIP2A protein.

A549 cells were transfected with mixtures of plasmids or siRNA and incubated for 72 h followed by cycloheximide

treatment. Cells were harvested at indicated period, and then subjected to the Western blot analysis.

## Figure S3


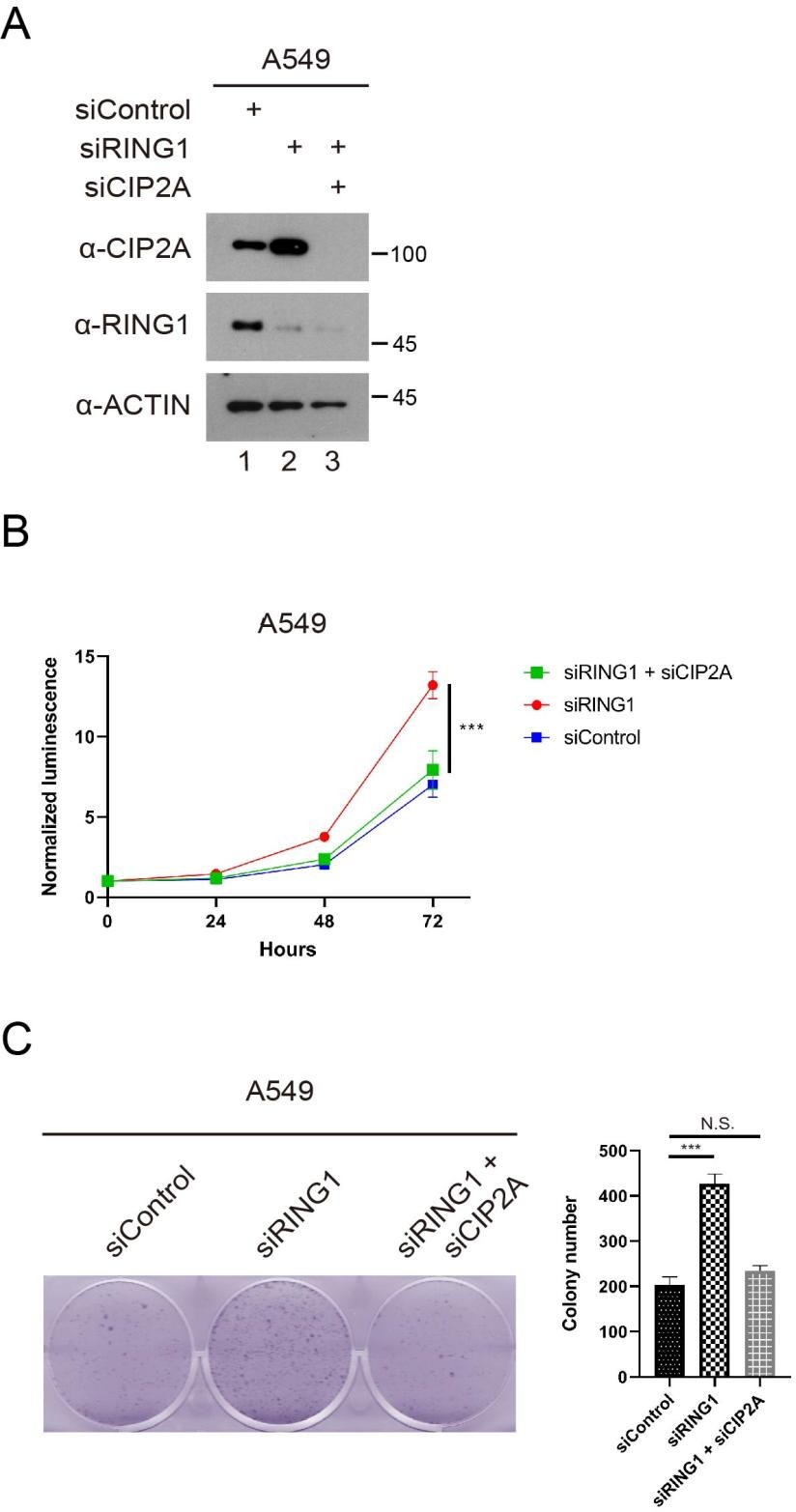


### Supplementary Figure 3. The effect of RING1 and CIP2A knockdown on lung cancer cell growth.

**(A)** A549 cells were seeded in 6-well plates and transfected with siRING1 and siCIP2A. After incubation, cells were harvested and immunoblotted for the indicated antibodies. **(B)** A549 cells were seeded in 96 well-white bottom dishes and monitored over 3 days using the CellTiter-Glo luminescence assay (n=5, with normalization at day 0). **(C)** A549 cells were seeded in 6-well plates and incubated for 7 days. The cells were then stained with crystal violet dye and counted. Data are expressed as mean ± SD from three independent experiments.

## Figure S4


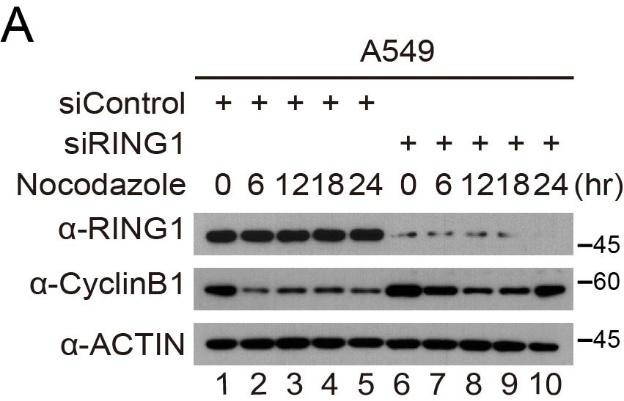


### Supplementary Figure 4. The effect of RING1 knockdown on the cell cycle progression

**(A)** A549 cells were transfected with siRNA against RING1 and then nocodazole was treated. After incubation, the media was replaced with fresh complete media for releasing cell cycle progression. Then cells were incubated for indicated periods and harvested for following Western blot analysis.

## Figure S5


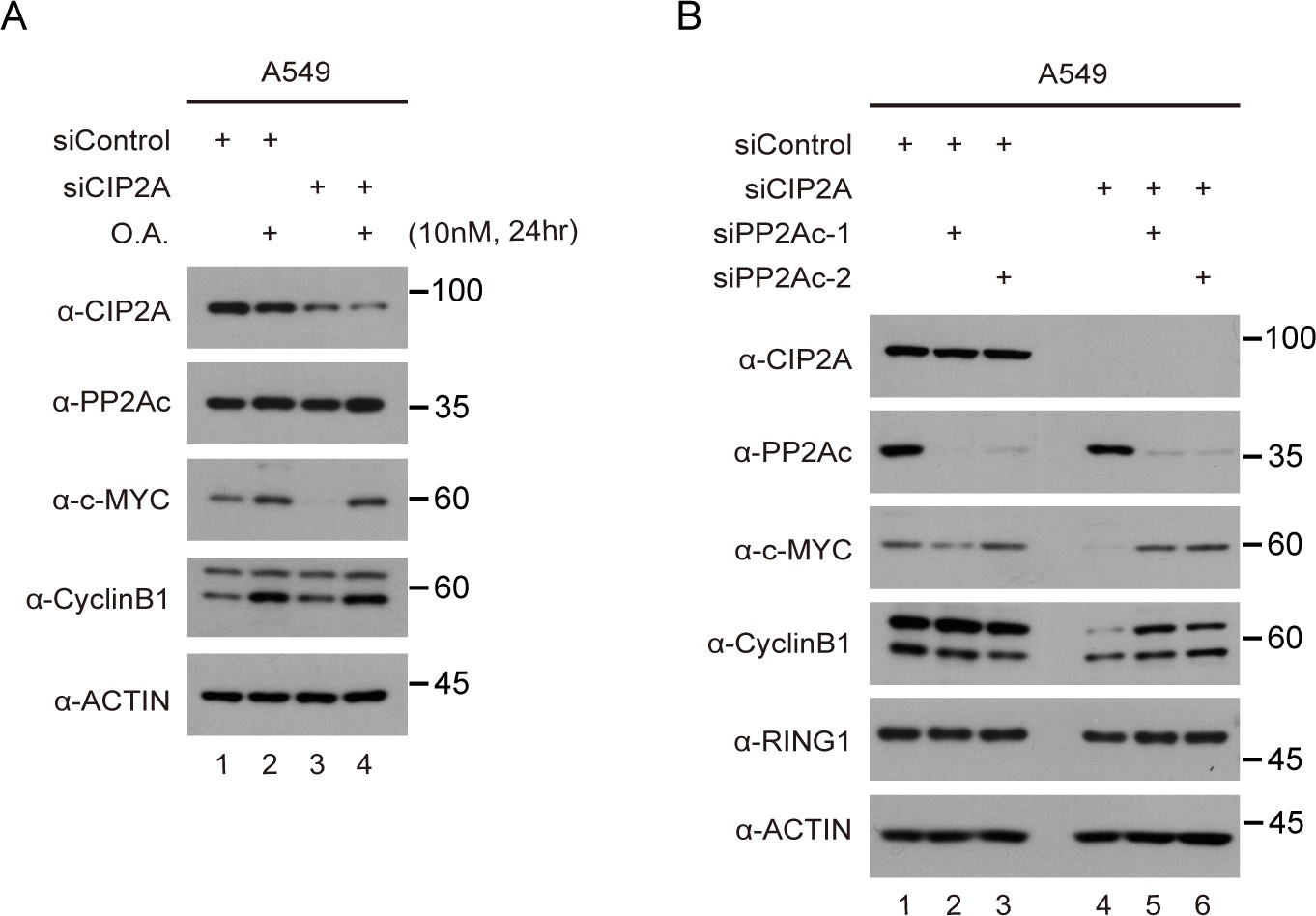


### Supplementary Figure 5. The effect of CIP2A knockdown and PP2A actitvity on c-Myc protein level.

**(A)** A549 cells were transfected with siRNA for CIP2A and then 10 nM of okadaic acid was treated and incubated for 24 h. Cells were harvested and subjected to immunoblotting for the c-Myc and cyclin B1 levels. **(B)** A549 cells were transfected with siRNA for CIP2A and PP2Ac. Cells were harvested and subjected to immunoblotting for the

c-Myc and cyclin B1 levels.

## Figure S6


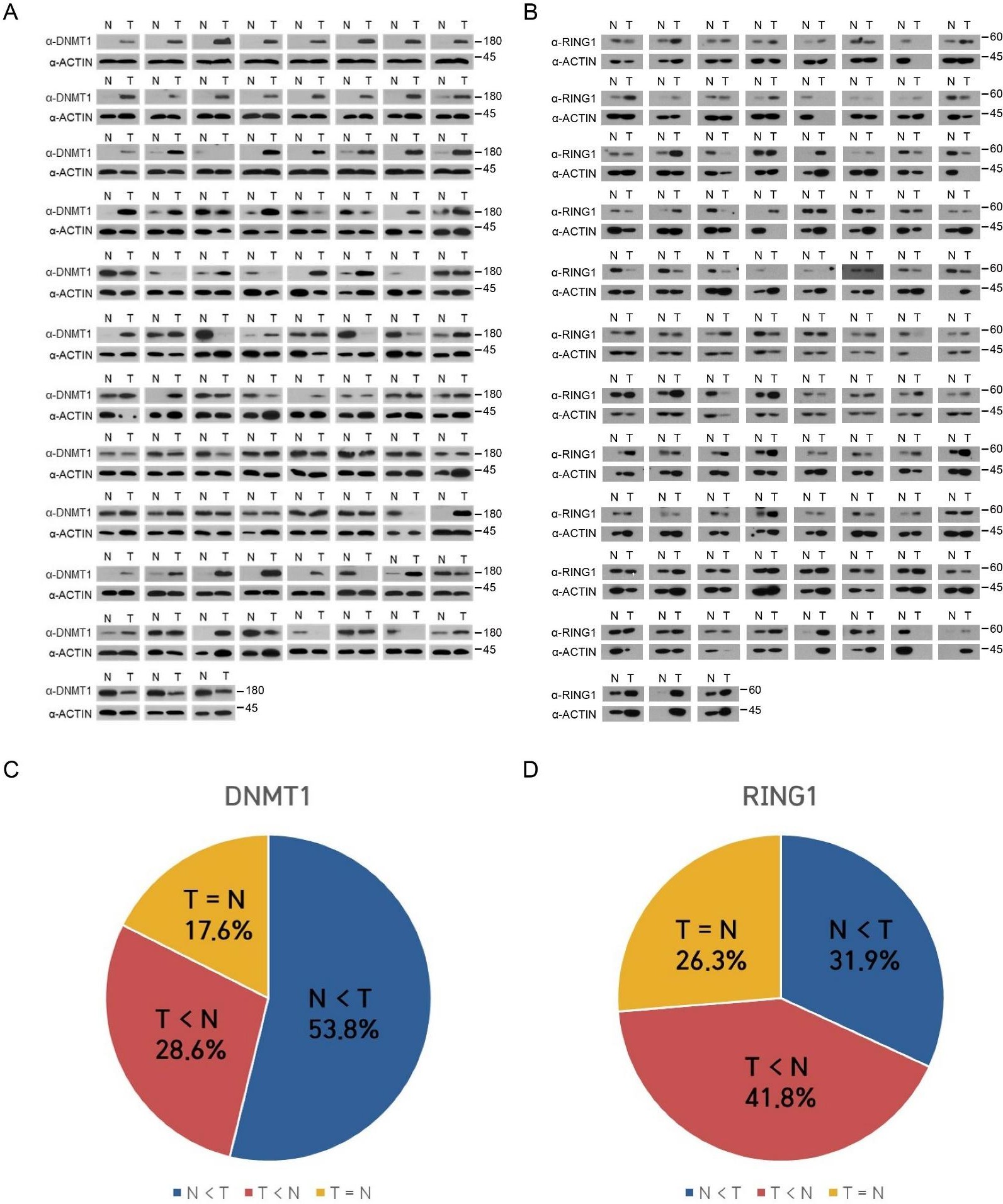


### Supplementary Figure 6. DNMT1 and RING1 protein level in tissues of lung cancer patients.

**(A-B)** Western blot results in 91 paired adjacent normal lung tissues (N) and tumor tissues (T). Each tissue was lysed by PRO-PREPTM solutions and immunoblotted with the indicated antibodies. **(C-D)** Chart illustrates the percentage distribution of DNMT1 and RING1 protein expression levels between tumor tissues and adjacent normal tissues.

## Figure S7


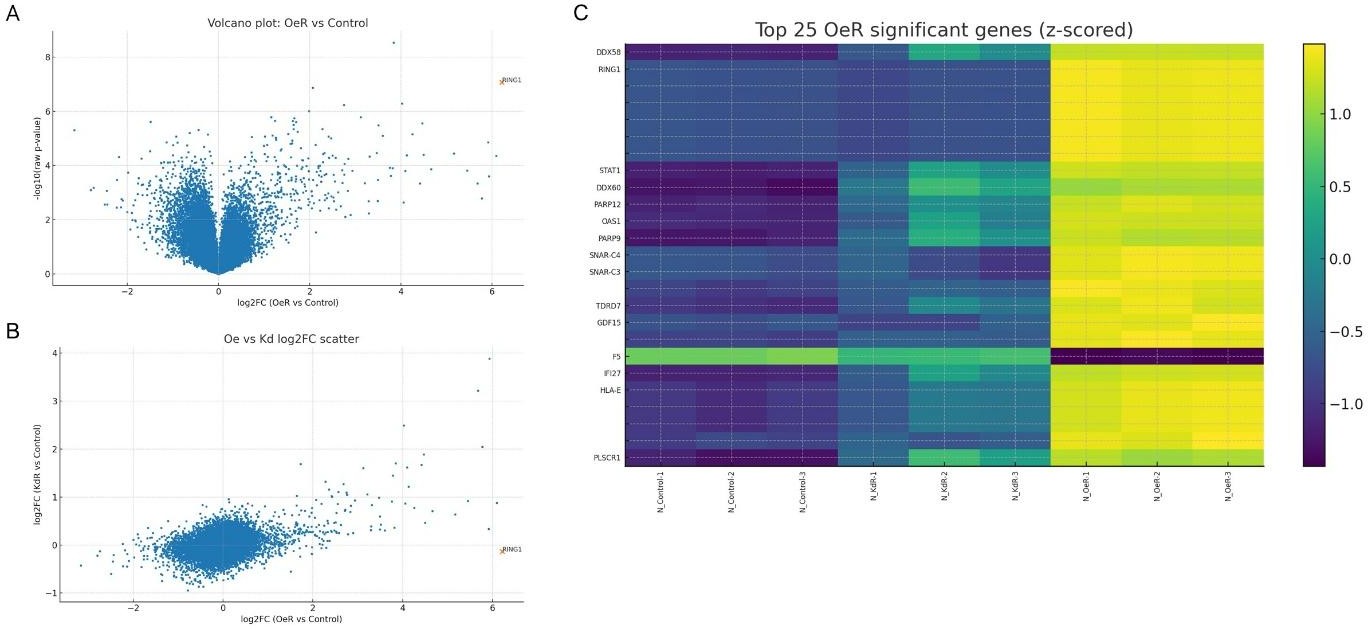


### Supplementary Figure 7. Transcriptomic profiling of RING1-overexpressing (OeR) and RING1-knockdown

**(KdR) A549 cells.**

we performed transcriptomic analysis using Affymetrix Human Gene ST 2.0 arrays in A549 cells with RING1 overexpression (OeR) and siRNA-mediated knockdown (KdR). **(A)** Volcano plot of OeR vs Control showing robust induction of interferon/antiviral genes, with RING1 highlighted. **(B)** Scatter plot of comparing OeR vs KdR. RING1 fold changes; overexpression showed significant effects, whereas knockdown showed more subtle changes.

**(C)** Heatmap of the top 25 significant genes in OeR (FDR ≤ 0.05), revealing strong enrichment of immune/antiviral pathways.
